# BioisoIdentifier: an online free tool to investigate local structural replacements from PDB

**DOI:** 10.1186/s13321-024-00801-8

**Published:** 2024-01-13

**Authors:** Tinghao Zhang, Shaohua Sun, Runzhou Wang, Ting Li, Bicheng Gan, Yuezhou Zhang

**Affiliations:** 1 Xi’an Institute of Flexible Electronics (IFE) and Xi’an Institute of Biomedical Materials & Engineering (IBME), Northwestern Polytechnical University, 127 West Youyi Road, Xi’an, 710072 China; 2 School of Management, Xi’an University of Architecture and Technology, Xi’an, 710055 China; 3 College of Petroleum Engineering, Northeast Petroleum University, Daqing, 163318 Heilongjiang China; 4 Ningbo Institute of Northwestern Polytechnical University, Frontiers Science Center for Flexible Electronics (FSCFE), Key Laboratory of Flexible Electronics of Zhejiang Province, Ningbo Institute of Northwestern Polytechnical University, 218 Qingyi Road, Ningbo, 315103 China

## Abstract

Within the realm of contemporary medicinal chemistry, bioisosteres are empirically used to enhance potency and selectivity, improve adsorption, distribution, metabolism, excretion and toxicity profiles of drug candidates. It is believed that bioisosteric know-how may help bypass granted patents or generate novel intellectual property for commercialization. Beside the synthetic expertise, the drug discovery process also depends on efficient in silico tools. We hereby present BioisoIdentifier (BII), a web server aiming to uncover bioisosteric information for specific fragment. Using the Protein Data Bank as source, and specific substructures that the user attempt to surrogate as input, BII tries to find suitable fragments that fit well within the local protein active site. BII is a powerful computational tool that offers the ligand design ideas for bioisosteric replacing. For the validation of BII, catechol is conceived as model fragment attempted to be replaced, and many ideas are successfully offered. These outputs are hierarchically grouped according to structural similarity, and clustered based on unsupervised machine learning algorithms. In summary, we constructed a user-friendly interface to enable the viewing of top-ranking molecules for further experimental exploration. This makes BII a highly valuable tool for drug discovery. The BII web server is freely available to researchers and can be accessed at http://www.aifordrugs.cn/index/. Scientific Contribution: By designing a more optimal computational process for mining bioisosteric replacements from the publicly accessible PDB database, then deployed on a web server for throughly free access for researchers. Additionally, machine learning methods are applied to cluster the bioisosteric replacements searched by the platform, making a scientific contribution to facilitate chemists’ selection of appropriate bioisosteric replacements. The number of bioisosteric replacements obtained using BII is significantly larger than the currently available platforms, which expanding the search space for effective local structural replacements.

**Graphical Abstract:**

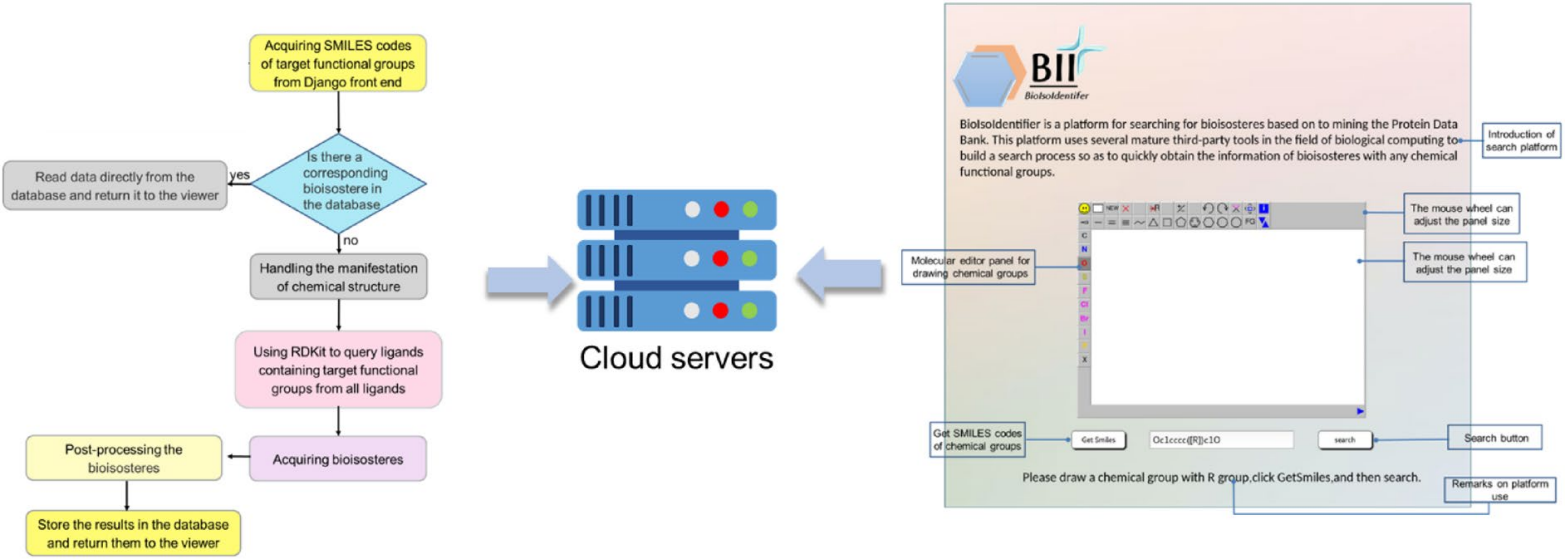

**Supplementary Information:**

The online version contains supplementary material available at 10.1186/s13321-024-00801-8.

## Introduction

It is essential to view databases not only as repositories of experimental results but also as valuable resources for data exploration and exploitation, particularly when mining data from publicly accessible databases. Among these, the Protein Data Bank (PDB), Cambridge Structural Database (CSD), and ChEMBL all contain rich implicit information that can be leveraged for drug discovery. ChEMBL, which aggregates chemical, bioactivity, and genomic data, is a meticulously curated database of bioactive molecules with drug-like properties [1]. EMBL-EBI recently released ChEMBL 30, which includes approximately 2.2 million compounds, 1.5 million assays, and 43,000 indications, all deposited and well-archived. Both CSD and PDB consist of ASCII files containing three-dimensional (3D) atomic coordinates of molecules, although they differ in terms of molecule size. Established in 1965, CSD serves as the global repository for organic crystal structures of small molecules, managed by the Cambridge Crystallographic Data Centre and updated thrice annually. As part of this commercialized project, several tools, including the CSD System, DASH, Mercury Menu, GOLD, and SuperStar, have been developed to provide comprehensive knowledge derived from CSD, making it widely utilized by the research and industrial communities.

Established in 1971 by the structural biology community as a central repository for macromolecular structure data, the PDB has consistently upheld a culture of open access and is now widely employed in fundamental biology, with millions of users leveraging its data to advance biomedical research [2]. Structural biology and structural bioinformatics have profoundly influenced our understanding of the mechanisms and functions of biological macromolecules. The PDB serves as a custodian for all this data, representing the repository for the vast majority of accomplishments and milestones in the structural biology community. It also offers numerous additional sequence and structural annotations, along with tools for pairwise and multiple structure comparisons, including those for the analysis of ligands and their interactions. Therefore, PDB has the potential to be further utilized for specific applications. The cheminformatics and bioinformatics knowledge within PDB can be extracted through in-silico parsing of textual files. For instance Borrel et al. characterized the frequency, type, and density of the salt bridges during the ligand-receptor recognition [3], which can greatly benefit drug design. However, the development of tools and applications based on PDB data has fallen short of expectations, not to mention commercialized products.

A key challenge for medicinal chemists is to modulate the potency and selectivity of small therapeutics toward their biological targets and some believe that bioisosteric replacement is an effective strategy to expedite the process of identifying analogues with improved potency, intending to bypass existing patents [4]. Bioisosterism, described as functional group exchanges to achieve similar biological outcomes, has garnered significant attention among practitioners. Bioisosteric replaceability relies on broader structural similarities to elicit the desired biological effects, rather than adhering strictly to physical or electronic mimicry. Typically, in medicinal chemistry, one modifies a promising pharmacophore by replacing specific functional groups with the aim of achieving the same biological response. Examples have demonstrated that bioisosterism is a powerful tool for guiding successful drug development projects [5]. The replacement of the amide moiety and benzene ring of the phase II clinical candidate GSK’772 led to the discovery of more potent compounds with EC_50_ values of 2.8 nM toward the target [6]. The surrogation of l-proline in melanostatin with 3-furoic acid has afforded two potent analogues with 2- and 4.3-fold improved EC_50_ to dopamine D_2_ receptors, respectively [7]. Instead of improving the potency of parent ligands by using local structural replacement approach, a brand-new molecule can also be created. Starting with a kinase inhibitor, Grigorii et al. searched for commercially available replacements of the individual building blocks that constitute the parent ligand, then determined which fragments were suitable for merging into new compounds with a high binding affinity [8]. Referring to bioisosteric replacements strategy, Yang et al. developed DrugSpaceX database which dramatically diversified the modifications of the molecular framework thereby extended drug space [9]. Bioisosteric replacement as a tool for either anti-HIV drug design [10] or specific chemical moieties, including amide [11], phenyl [12] has been reviewed.

From a molecular perspective, bioisosteric replacement enable the conservative interactions between a ligand and a target protein [13] and this mutual recognition can be depicted *in silicon*. Nowadays, computational tools have become indispensable in drug discovery process and have emerged to accelerate the acquisition of bioisosteric information from bio- or/and cheminformatic database. Analysing data from the PDB, the investigation into tetrazole-carboxylic acid bioisosterism revealed that protein binding site needs to be flexible enough to establish robust hydrogen bonds with tetrazolate ligands, especially when compared to carboxylate counterparts [14]. In a computational lead optimization process using bioisosterism, structural data of the target protein–ligand complex are leveraged [15] to modify the parent scaffold, following the principle of ensuring a suitable fit and interaction compatibility within the specific subpocket of the target protein [16]. Other than the extraction of bioisosteric information through computational tools, the identification of appropriate bioisosteres heavily relies on the experience of individual practitioners, making it subjective and potentially influenced by personal biases. While these semiempirical methods have been praised for offering alternatives, they frequently fall short in elucidating the underlying interaction mechanisms, particularly in how the bioisostere in question consistently interacts with the receptor in comparison to the reference moiety. Furthermore, having an excessive number of bioisosteres to choose from without proper organization and categorization could lead to the pitfalls of trial-and-error screening, frustrating researchers who prefer a clear ranking of top candidates. As drug development costs rise, there is a growing need for a user-friendly, readily applicable system for bioisosteric information. However, it is currently lacking in this regard.

Due to the discrepancy between the vast, but underused data repository and the increasing demand of medicinal chemists for valuable bioisosteres, especially those with implicit characteristics that are difficult to imagine or have not been previously experienced, there is a pressing need for computational methods that can efficiently traverse the database for such information. SwissBioisostere, hosted by the Swiss Institute of Bioinformatics and being accessible via a web interface [17], uses the ChEMBL database as a primary data source to identify matched molecular pairs by applying the Hussain and Rea algorithm after data curation. sc-PDB-Frag [18], differentiating from ligand based scaffold hopping, searches bioisosteric replacements from the protein–ligand interaction pattern. In contrast, KRIPO [19], quantifies the similarities of binding site subpockets not only intra- but also interprotein family, broadening the application spectrum of bioisosterism. Seddon et al. fragmented the ligands for a given target using the BRICS scheme, then considered a pair of extracted moieties to be bioisosteric if they occupy a similar volume of the protein binding site [20].

A web tool to automate bioisosteric functional groups identification was developed by Novartis through the calculation of electronic, hydrophobic, steric, and hydrogen bonding properties as well as by the drug-likeness index of about 8.5 million unique organic substituents [21]. The web server MolOpt assists in drug design using bioisosteric transformations, with rules derived from data mining, deep generative machine learning, and similarity comparisons [22]. After the input of a protein and a ligand structure and users’ selection of specific substructures which intended to replace, computational tool FragRep [23] tried to find suitable fragments that simultaneously match the geometric requirements of the remaining part of the ligand and well complementary with local protein environments. One crucial aspect of structure-based drug design is the use of GRID software to identify potential chemical modifications that can be made to known ligands. Recently Cross et al. proposed FragExplorer approach aiming to show users which fragments would best match the GRID molecular interaction fields in a protein binding pocket [24]. Craig Plot 2.0 fragmented ChEMBL database bioactive molecules, determined Hammett σ and Hansch-Fujita π values for their substituents, and grouped them by root or atom type, aiding in the selection of bioisosteric analogs [25].

Successful application of bioisosteric transformation hinges upon a thorough understanding of the physicochemical attributes of frequently encountered substituents, which can be accurately represented. For example, R-group descriptors encoding the distribution of atomic properties at increasing distances from a substituent’s point-of-attachment to a central ring scaffold for identifying structurally similar pairs of substituents were reported by Holliday et al. [26] 3D descriptors Flexsim-R were calculated based on docking of small building blocks drug-like molecules into a reference panel of protein binding sites for bioisosteric functional groups [27]. So far, the acquisition of the bioisosteric information depending on (1) the experience of medicinal chemists working many years in the field; (2) mining the medicinal chemistry literature and extracting information by querying an internal library containing bioisosteric families [28]; (3) similarity in molecular physicochemical properties, including size, hydrophobicity, 3D substituents [29] or electron-donating profiles and (4) deep neural network trained on experimentally validated analogues extracted from medicinal chemistry literature [30].

The structural replacement of phosphate [31] and ribose [32] group identification was executed using our previously developed computational workflow, yielding some intriguing results. This protocol can be streamlined and led to the development of a user-friendly web server, BioisoIdentifier (BII), equipped with fragment sketching tools. The process involves drawing the replacement fragment, converting it into Simplified Molecular Input Line Entry System (SMILES) code, and then processing it through the main program (Python and R). The program interfaces with third-party software, including Blastp, US-align, and RDKit, to organize individual PDB files. In this virtual system, spherical probes (2.5 Å radius) are created, targeting atoms within the reference ligand's chemical moiety for replacement as centroids. The sensed atoms serve as structural replacements for the reference fragment. To enhance output visualization, potential bioisosteric moieties are clustered based on structural similarity or unsupervised machine learning.

## Method

### Workflow of BII

**Fig. 1:**
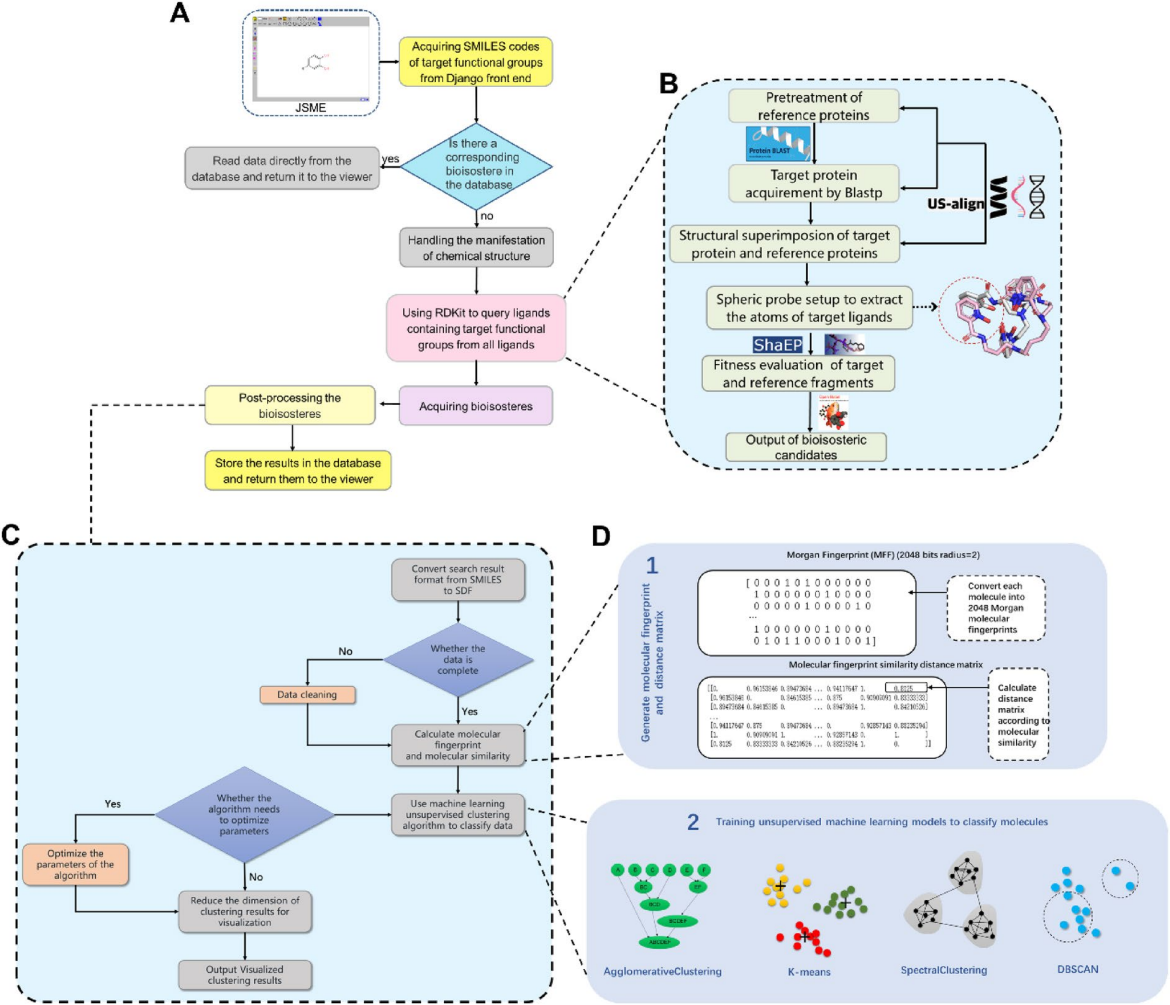
The workflow of BioisoIdentifier (BII) to identify the local structural replacements (LSR). **A** The complete workflow of BII; **B** the calculation process of obtaining LSR; **C** the process of LSR clustering with unsupervised algorithms; **D** calculation of molecular fingerprint, molecular similarity, and conduct unsupervised clustering

BII identifies bioisosteres in six steps, as illustrated in Fig. 1. Users sketch the target functional group using JSME in the Django frontend and obtain the SMILES code, which is transmitted to the backend. The backend searches the database for stored bioisosteres based on the provided SMILES code. If found, results are directly retrieved. If not, further processing occurs, with ligands containing the target functional group queried from the PDB using RDKit's substructure search. These reference ligands undergo a sequential search to obtain and save bioisosteres. The notable benefit of this approach arises from its ability to be explained through a molecular interaction perspective, leveraging information derived from PDB data to uncover details about local structural replacements. Figure 1B illustrates the specific calculation process.

1. PDB download: RCSB PDB provides a shell script, named “batch_download.sh” (in S1), which can download multiple PDB archive files by providing a file containing a comma-separated list of PDB IDs. An essential prerequisite for running this script is to have the ‘curl’ tool installed. However, during our attempts to acquire the PDB archive, we encountered slow download speeds. Therefore, we developed a Python-based web crawler to swiftly retrieve the data.
2. Pretreatment of target protein: The small-molecule ligands with substructures intended to be bioisosterically replaced are selected from the PDB archive, with the macromolecular structures containing these ligands serving as reference proteins. We obtain the FASTA sequences of these proteins and input them into Blastp [33] to compare them with the sequences in the PDB, then output protein homologues with very close or identical structure.
3. Protein structure superimposition: Protein homologues exhibiting remarkably similar or identical structures are meticulously superimposed onto the reference protein using TM-align [34]. Subsequently, these alignments are further refined through the application of US-align [35] to achieve a more precise protein structure alignment.
4. Local structure extraction: Upon the successful alignment of these protein homologues, the atomic coordinates of the reference fragment earmarked for replacement within the reference protein are extracted. Each atom of the fragment functions as the centroid of a sphere with a radius of 2.5 Å. These spheres are employed to explore target ligand fragments, capturing atoms that come into contact, which are subsequently extracted and regarded as potential bioisosteric replacements for the reference substructure.
5. Fitness evaluation of extracted fragment with reference substructure: To assess the extent of overlap between the extracted fragments and the reference moiety, we utilized ShaEP [36], a tool designed for evaluating the similarity of ligand-sized molecules in terms of both shape and electrostatic potential. As per its definition, the fitness of a molecule pair based on ShaEP falls within the range of [0,1], with 1 signifying a perfect match. In this context, we established a threshold of 0.2 based on empirical rules and experience.
6. Output of extracted fragment with SMILES code: While computers are well-suited for processing textual strings, the human brain often finds graphical information more intuitive and comfortable to work with. To address both of these requirements, Open Babel [37], which enables the interconversion of more than 100 formats of chemical structures, was employed to specifically convert the SMILES string into an output fragment graph.

To classify the structural isosteres of the 3-substituted catechol, a clustering post-processing step was employed, utilizing unsupervised machine learning. In this regard, several algorithms were experimented with and underwent parameter adjustments to optimize each one individually. The detailed process is illustrated in Fig. 1C and is described as follows:

1. Search result format conversion: To calculate molecular similarity for the subsequent calculations, the format of all search results was converted from SMILES to SDF format using custom-written code. Converting from SMILES to SDF format can result in potential loss of information. As a precaution, it is necessary to clean the data, which involves removing entries with missing content and eliminating duplicates.
2. Molecular fingerprint and molecular similarity calculation: The molecular Morgan fingerprints were calculated at first, and then the RDKit tool was used to calculate the molecular similarity matrix through Tanimoto distance, as depicted in the zoomed-in view in Fig. 1D1
3. Data classification by using machine learning unsupervised clustering algorithms: we explored the application of various unsupervised clustering algorithms, as illustrated in Fig. 1D2. These algorithms can be broadly categorized into two groups. The first category comprises algorithms like K-means and Dbscan, which necessitate specifying the hyperparameter for the number of clusters. In contrast, the second category includes algorithms such as AgglomerativeClustering and AffinityPropagation, which do not require specifying the number of clusters.
4. Optimization of algorithms parameters: For algorithms that necessitate the specification of additional hyperparameters, including the number of clusters, we employed techniques like the elbow method, silhouette coefficient method, and hyperparameter random search to optimize the clustering results by searching for the best parameters.
5. Dimension reduction of clustering results for visualization: As previously mentioned, data points are stored in the form of 2048-bit MFF, which makes it challenging to effectively visualize clustering results in such high-dimensional space. Therefore, we employ principal component analysis (PCA) to reduce the data dimension from 2048 dimensions to 2D or 3D. We utilize the matplotlib tool to create visual representations and display the clustering results graphically.

### ***Web ***server

#### Interface features and usage

**Fig. 2:**
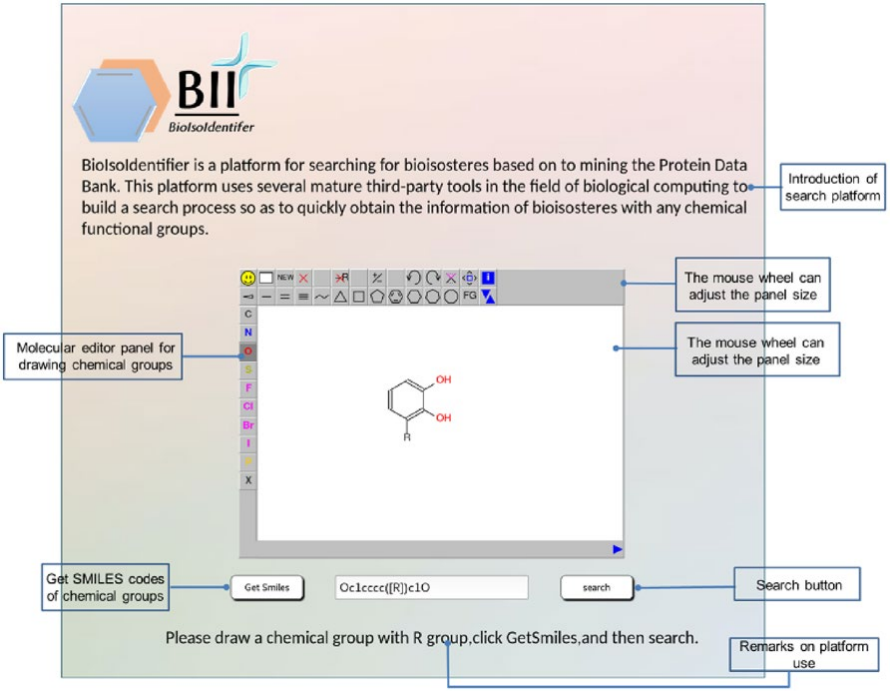
The interface of BII

Figure 2 displays a screenshot of the BII homepage, featuring a concise introduction and a web server input interface. Users can draw the chemical structure of the target functional groups in the molecular editor JSME. The ‘R’ denotes the vertex where the target functional group bifurcates, indicating that only the sketched core substructure requires replacement. The input fragment is always assumed to be complete. Once the structural construction is complete, users can obtain the SMILES code corresponding to the target functional group by clicking the “Get Smiles” button on the page. Subsequently, they can initiate the LSR search by clicking the “search” button.

#### Implementation

The Django web framework and Python code are employed to develop the interface functionality of the web server and execute MySQL database queries for ligand substructure replacement. RDKit [38] is utilzied to facilitate fragment database construction, calculate molecular descriptors, and depict 2D molecular structures.

#### Case study

**Fig. 3:**
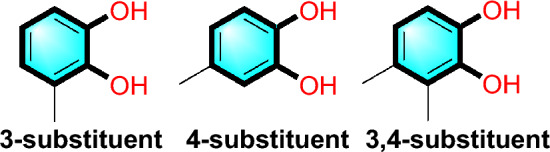
Three possible catechol containing ligands

Catechol, an unsaturated six-carbon ring (phenolic group) with two hydroxyl groups attached to adjacent carbons (dihydroxyphenol), is a widely observed group in neurotransmitters such as dopamine and noradrenaline. The nitrocatechol based compounds tolcapone and entacapone are successfully used as adjuncts to treat Parkinson’s Disease. Meanwhile, bisubstrate and non-nitro hydroxypyridone catechol *O*-methyltransferase (COMT) inhibitors have also been reported for the same disease. However, tolcapone and entacapone mainly act peripherally and poorly penetrate brain as centrally acting drugs. Besides, phenolic compounds are prone to high metabolic clearance due to their acidity and polarity. Therefore, next generation COMT inhibitor prefer replace catechol with corresponding bioisostere [39]. This need has drawn our attention to explore catechol bioisosteres, which we present as a case study. Apart from the two contact points of the hydroxyl group in the benzene ring, four other positions are available for ligand extension, representing three types (Fig. 3) of possible catechol containing ligands.

## Results and discussion

### The LSR of catechol

**Fig. 4:**
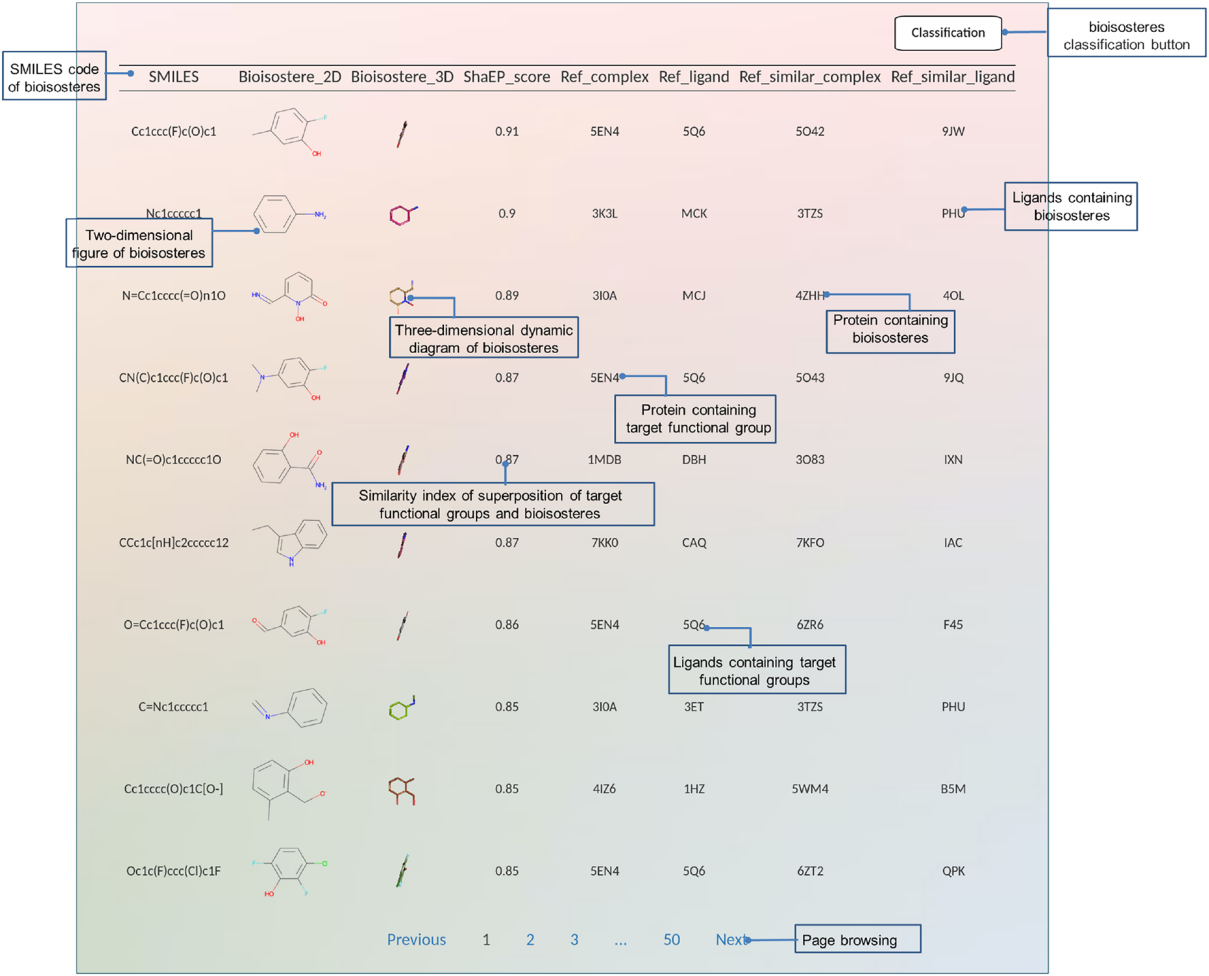
The LSR list of 3-substituent catechol

When inputting a 3-substituted catechol encoded as ***Oc1cccc([R])c1O*** into the server, it suggests over 496 replacement ideas, all of which are displayed in a table, paginated for convenience. Figure 4 provides a snapshot of the first page, showcasing the clustering results represented in both two-dimensional and three-dimensional structures. The remaining replacements are documented in Additional file 1: Figure S2. Each entry in the table includes valuable information such as SMILE codes, 2D and 3D representations, a similarity index, as well as the associated reference protein complex and its corresponding ligand PDB ID, along with details of the target protein complex and its related ligand PDB ID.

**Fig. 5:**
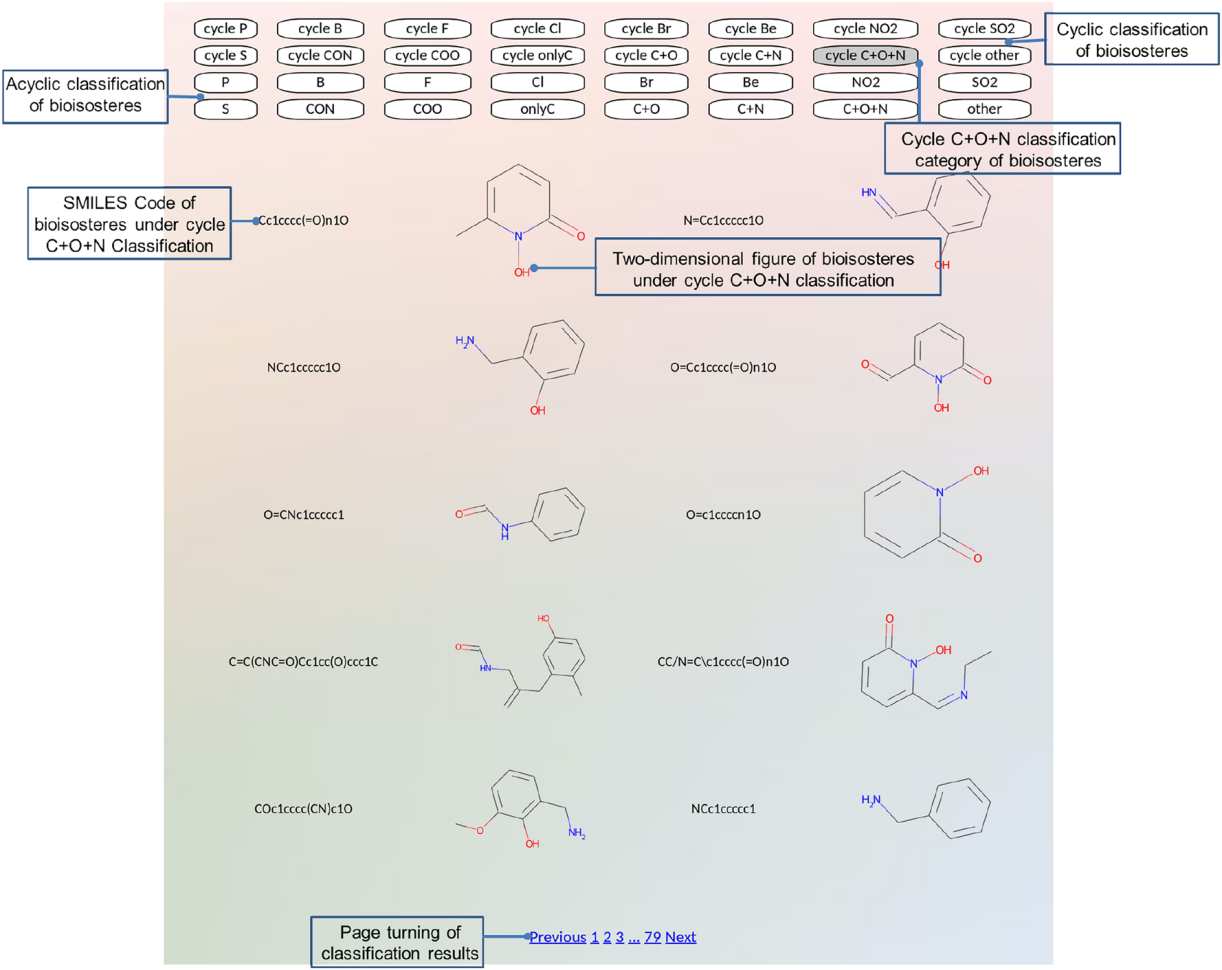
The LSR subgroup of 3-substituent catechol categorized as cycle C+O+N

The LSR of 3-substituented catechol are first sorted according to their ShaEP index and subsequently recorded in a table. Based on their structural similarity, they are then hierarchically classified into 32 distinct groups. Users can easily visualize this classification by clicking on the “Classification” tab. For a more detailed view, specific LSR included in the “C+O+N” group are exemplified in Fig. 5, accessible by clicking the corresponding group name. Moreover, unsupervised learning algorithms have been employed to further refine and narrow down the number of subgroups.

**Fig. 6:**
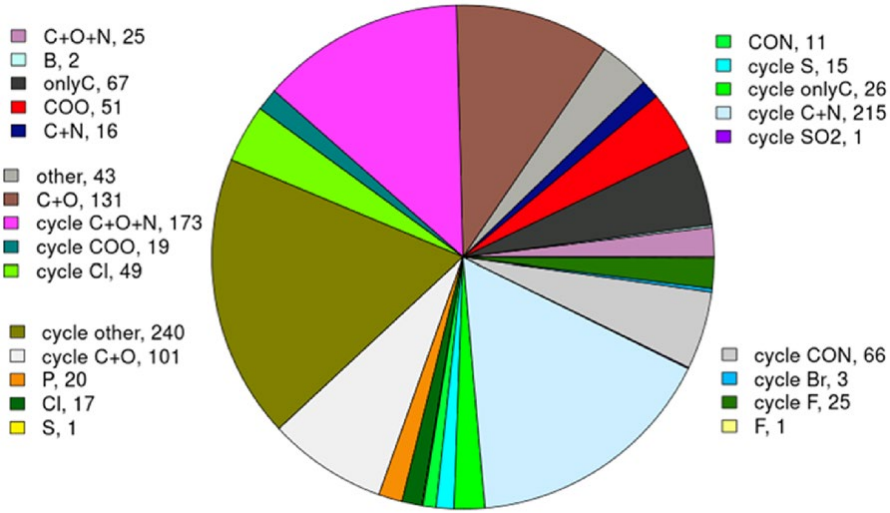
Distribution of the data set into categories assigned based on the SMILES codes of the structural isosteres of 3-substituent catechol

Figure 6 illustrates the categorization of LSR for 3-substitued catechol recognized using BII. They are sorted into 24 categories based on the SMILES code. Among these, 240 bioisosteres, although belonging to cyclic structures, do not fall into any predefined category; therefore, they are grouped under [cycle other], making it the largest family. This is followed by 215 members categorized under [cycle C+N], and there is only one bioisostere in the [F] category. For further insights, bioisosteres of 4-substituted and 3,4-substituted catechol are also presented individually in Additional file 1: Figure S3 and S4. Notably, the primary focus of this work is on the conservativity of interactions between the parent ligand moiety and the protein, without explicitly discriminating between the replacement of the moiety and the generation of entirely new molecules. While BII may suggest local structural replacements for specific moieties in the catechol example, our goal is to identify bioisosteric replacements with greater stringency. Our approach involves superimposing proteins with identical groups but accommodating different ligands. We then concentrate on the space where the intended moiety is to be replaced. The docking of replacement moieties into the original catechol's position may induce a shape change in the binding pocket due to its flexibility. Importantly, our approach can be applied to scaffold hopping and the generation of combinatorial libraries to a certain extent.

**Fig. 7:**
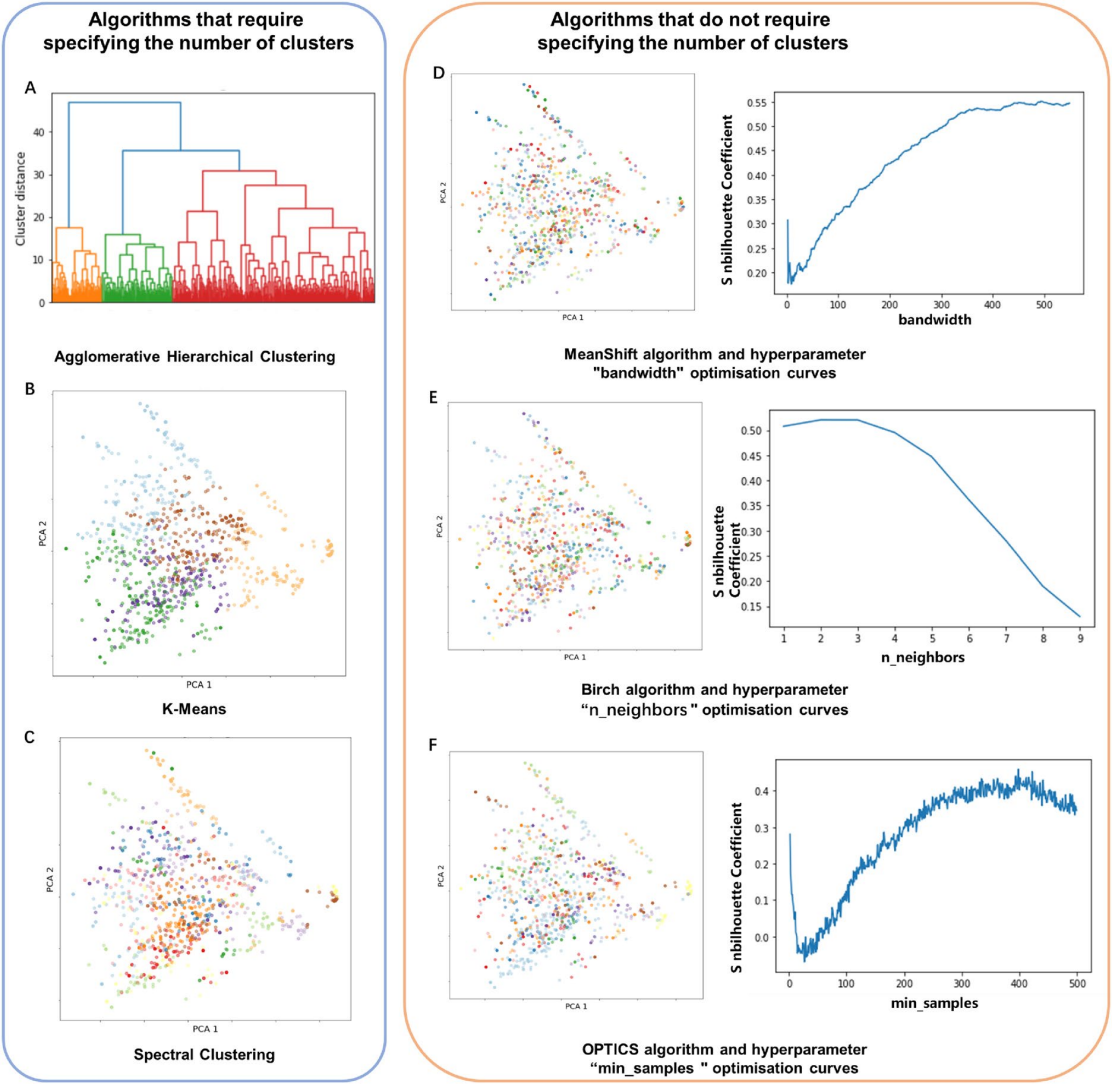
Results using algorithms without hyperparameters and algorithms requiring hyperparameters. **A** Agglomerative Hierarchical Clustering; **B** K-Means Clustering; **C** spectral clustering; **D** MeanShift algorithm and hyperparameter “bandwidth” optimisation curves; **E** Birch algorithm and hyperparameter “n_neighbors " optimisation curves; **F** OPTICS algorithm and hyperparameter “min_samples” optimisation curves

Unsupervised clustering methods are employed to categorize structural replacements of 3-substituent catechol into fewer categories, utilizing the SMILES encoding approach. This unsupervised clustering unveils latent similarities among these structural replacements, thereby simplifying data complexity and enhancing comprehensibility and visualization. This simplification streamlines the selection of representative samples from each cluster, facilitating in-depth research and, consequently, enhancing screening efficiency. In Fig. 7, you can observe the results obtained from the application of various algorithms and their respective optimization techniques. The algorithms are divided into two categories based on the necessity of pre-specifying the number of clusters, each category employing unique hyperparameter optimization strategies. For algorithms where pre-specifying the cluster number is unnecessary, as exemplified by the MeanShift algorithm, we construct an optimization curve that correlates the “bandwidth” hyperparameter with the silhouette coefficient to determine the optimal “bandwidth” value of 446. This corresponds to a cluster count of 47 with an average silhouette coefficient of 0.561. The Birch clustering algorithm employs a similar approach to ascertain the optimal “n_neighbors” hyperparameter value, achieving the highest silhouette coefficient of 0.519 when “n_neighbors” equals 3. In the case of algorithms requiring a predefined number of cluster groups, a more intricate method is employed to determine the optimal cluster count.

**Fig. 8:**
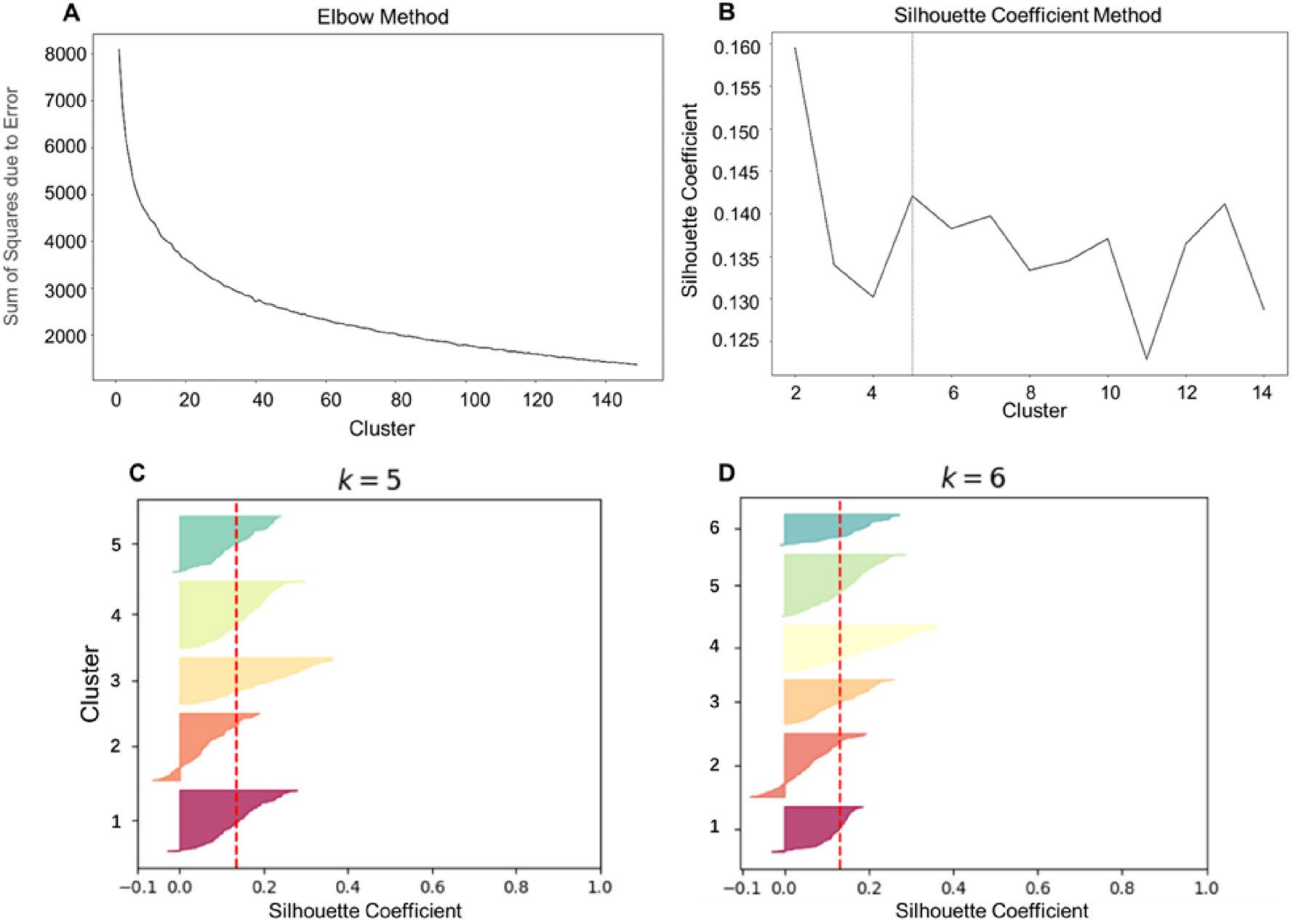
The strategy for determining the optimal number of clusters for the K-Means algorithm. **A** The optimal cluster number determination using elbow rule; **B** the optimal cluster number determination using contour coefficient; **C**, **D** determine the optimal number of clusters by comparing the contour coefficients of different clusters

Figure 8 illustrates the process of determining the optimal number of clusters for the K-Means algorithm. The optimal number of clusters was determined using the elbow rule and the silhouette coefficient method, individually for rational segregation of the structural replacements in the chemical space. The elbow method and silhouette coefficient method are used to determine the optimal number of clusters. Figure 8A shows that the elbow of the sum of squares due to error (SSE) sharply drops when the number of classes is less than 15. It can be observed that the largest value of k for the contour coefficient is 2. However, the elbow diagram of k and SSE reveals that the SSE is still relatively large when k is taken as 2. This is due to that the contour coefficient takes into account the degree of separation, and so it is an irrational number of clusters for k = 2. Therefore, retreating to the second largest value of k for the contour coefficient, we consider the second largest value of k for the contour coefficient. Further analysis of the relationship between the silhouette coefficient and the number of clusters (Fig. 8B) reveals that the best cluster number (the number of clusters with the maximum silhouette coefficient) is 5. To verify this conclusion, silhouette coefficient diagrams for each class were plotted separately for clustering with 5 and 6 classes, and the average silhouette coefficients of the clustering results are indicated by the red dashed line. As shown in Figs. 8C and D, each class was more uniformly distributed when the cluster number was 5, supporting the empirical division of the LSR of 3-substituent catechol into 5 groups accordingly. It should be noted that the presented computational results are illustrative of our computational process using 3-substited catechol as an example, which is why some algorithms may have lower silhouette scores.

**Fig. 9:**
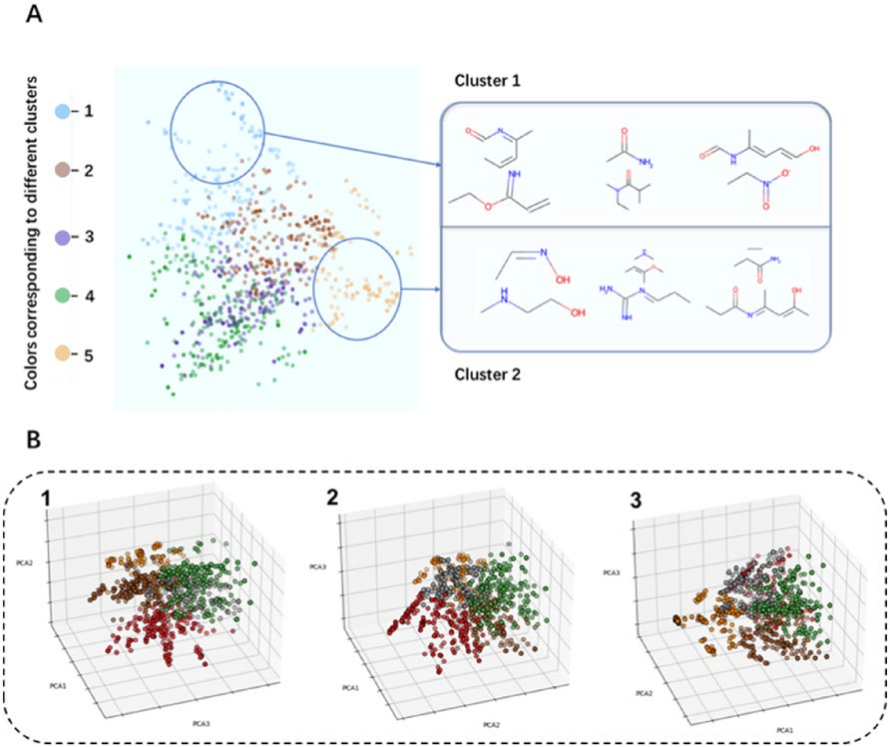
K-Means algorithm clustering results visualised by PCA (principal component analysis) for dimensionality reduction. **A** Two-dimensional visualization clustering space; **B** 3D visualization clustering space; B1 front view; B2 left view; B3 vertical view

To provide a detailed view of the clustering results of 3-substituted catechol LSR, principal component analysis (PCA) was employed to reduce the dimensionality of the 2048-dimensional data to 2D or 3D, as demonstrated in Fig. 9A for 2D visualization and Fig. 9B for additional perspectives on the 2D and 3D visualization, which are summarized in Additional file 1: Figure S5. In Fig. 9, dots of the same color represent a category, and two categories are chosen as examples to present a list of classified molecules. The acidity dissociation constants for catechol are p*K*_a1_ of 9.25 and p*K*_a2_ of 13.0 [40], suggested that the catechol is slightly acidic at biological environment of pH 7.4, it is therefore thought acidic groups are intrinsic biosisosteres of catechol to conserve molecular interactions where possible. However, we envision it is likely that basic groups might be suggested by our BII tool. It is not surprise since our previous investigation revealed that basic –CH_2_NH_3_^+^ replaced acidic phosphate group and a Mg^2+^ concurrently [31]. The metal cations hence may play an important role during local structure replacement of catechol since they can readily coordinate.

**Fig. 10:**
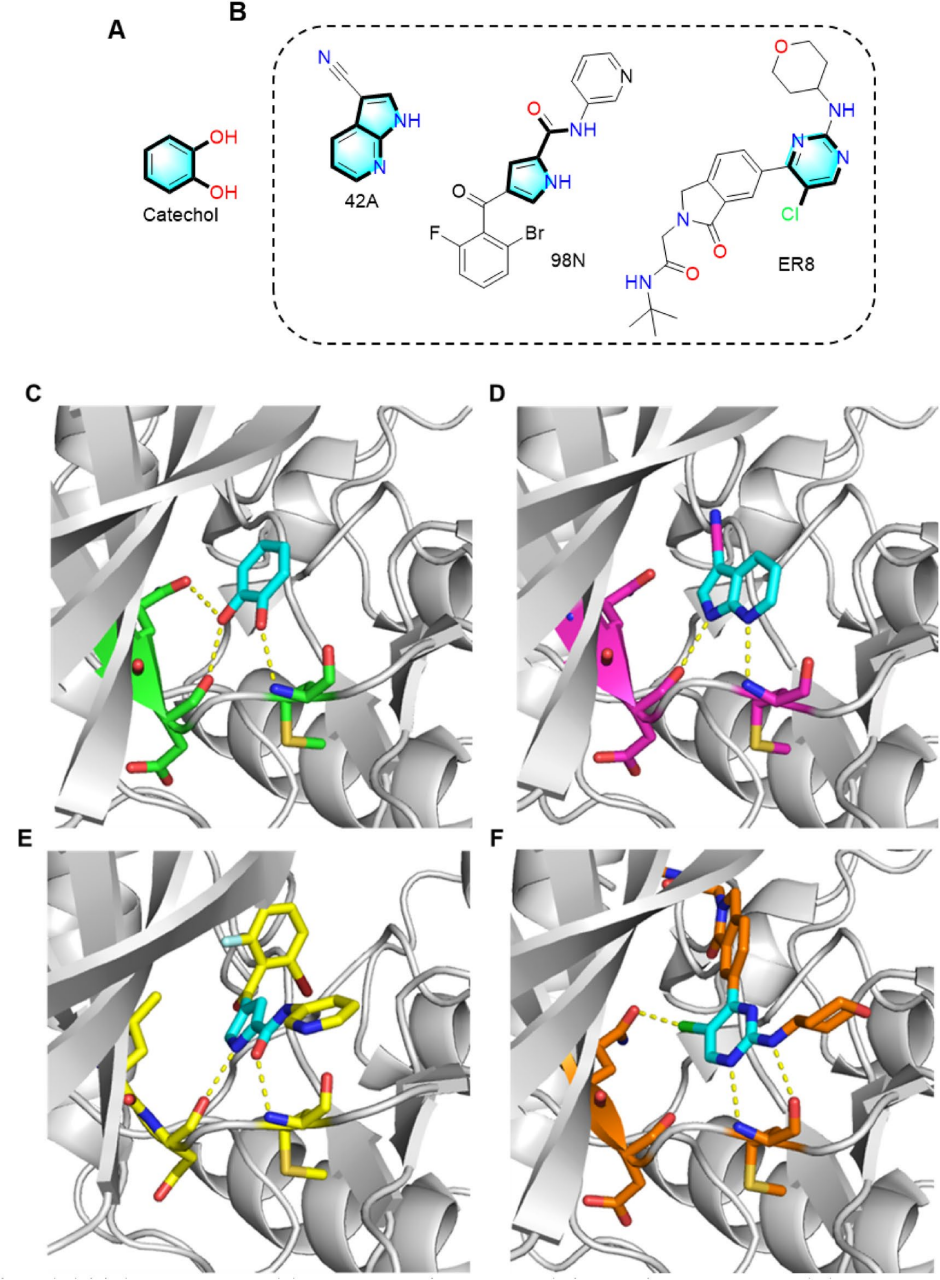
**A** Structure of Catechol. **B** Three active ERK2 inhibitors suggested from a BII search. **C**–**F** Interaction networks between 42A and 98N, ER8, MWL and ERK2/MAPK, respectively. In this figure, the ligands are named according to their PDB 3-lettercodes, and the proteins are named according to PDB4-lettercodes. Putative hydrogen bonds are shown as yellow dotted lines and the distance is labelled. The carbon atoms of structural replacements in the target ligand are highlighted in cyan, while others are shown in green, purple, yellow and brown, respectively

Three optional LSRs of catechol are displayed in Fig. 10, where it can be observed that these newly identified substructures exhibit similarities in shape to catechol. To elucidate structure–activity relationship of catechol and corresponding replacements, the structural and biological data are compiled from reference publications. In addition, we leveraged the structure diversification of identified new chemicals with activity change toward a selected target, discussed how substitutes deletion or protrusion impacts the biological activity of resulting molecules. The therapeutic impact of catechol in lung cancer treatment was achieved by inhibiting the activity of extracellular signal-regulated kinase 2 (ERK2), and its direct binding to the active site of ERK2 (PDB code: 4ZXT) was confirmed through X-ray crystallography [41]. Catechol was anchored to the hinge loop of the ATP-binding site of ERK2, with its hydroxyl groups interacting with the main chain of Asp106, Met108, and the side chain of Gln105, all located on the hinge loop. The azaindole ligand (compound 3 in Ref. [42] PDB code: 42A) occupied the same binding site where catechol was positioned in ERK2. In detail, the pyrrole NH of 7-azaindole formed a strong hydrogen bond (d = 2.8 Å) with the backbone carboxyl oxygen of Asp104, and the pyridine nitrogen served as a hydrogen bond acceptor (d = 3.0 Å) for the Met106 backbone NH. The ligand (compound 46 in Ref. [43] PDB code: 9N8) binds in the ATP-binding site of ERK5.

The pyrrole NH and amide carbonyl formed hydrogen bonds (d = 2.8 Å, d = 2.7 Å) with the backbone carbonyl of Asp138 and amide of Met140 in the ERK5 hinge-region, respectively. Noticeably, the pyrrole-2-carboxamide took the position of catechol. The chloro-substituted aminopyrimidine moiety of ER8 (compound 15 in Ref. [44]) took the space of catechol as so that halogen bond (d = 2.7 Å) between the the chloro atom and amide residue oxygen of gatekeeper Gln105. Hydrogen bonds (d = 3.1 Å, d = 2.9 Å) were observed between the ligand’s pyrimidine N, amino NH and the backbone NH, C=O of hinge residue Met108 respectively. C=O of hinge residue Met108 respectively. The p38αMAPK inhibitor hit (compound 3 in Ref. [45] PDB code: MWL) occupied the active site space of p38αMAPK.

The pyridine ring nitrogen allowed for hydrogen bonding (d = 2.8 Å) with the peptide backbone of Met109 from the hinge region. In this context, the pyridine moiety can be considered a structural replacement for the C=O of hinge residue Met108, effectively taking the place of catechol. The idea bioisosteres by definition, entails both steric and but electronic conservatism. However, achieving a perfect match for both criteria simultaneously can be challenging and may require some degree of compromise. It's conceivable that an imperfect match in electronic conservativity could be compensated for by a precise steric fit, thereby maintaining overall binding affinity. It should be acknowledged that the inability of BII to distinguish between hydrogen bond donors and acceptors, as it primarily focuses on the conservativity of the interaction itself. For instance, the hydroxyl group in catechol serves as a hydrogen bond receptor in the reference, whereas the –C=O group of the carboxamide in ligand 9N8 can only function as a hydrogen bond (HD) acceptor due to its electron-rich nature. The same applies to the cationic –N(CH_3_)– group, which acts as a HD acceptor.

**Fig. 11:**
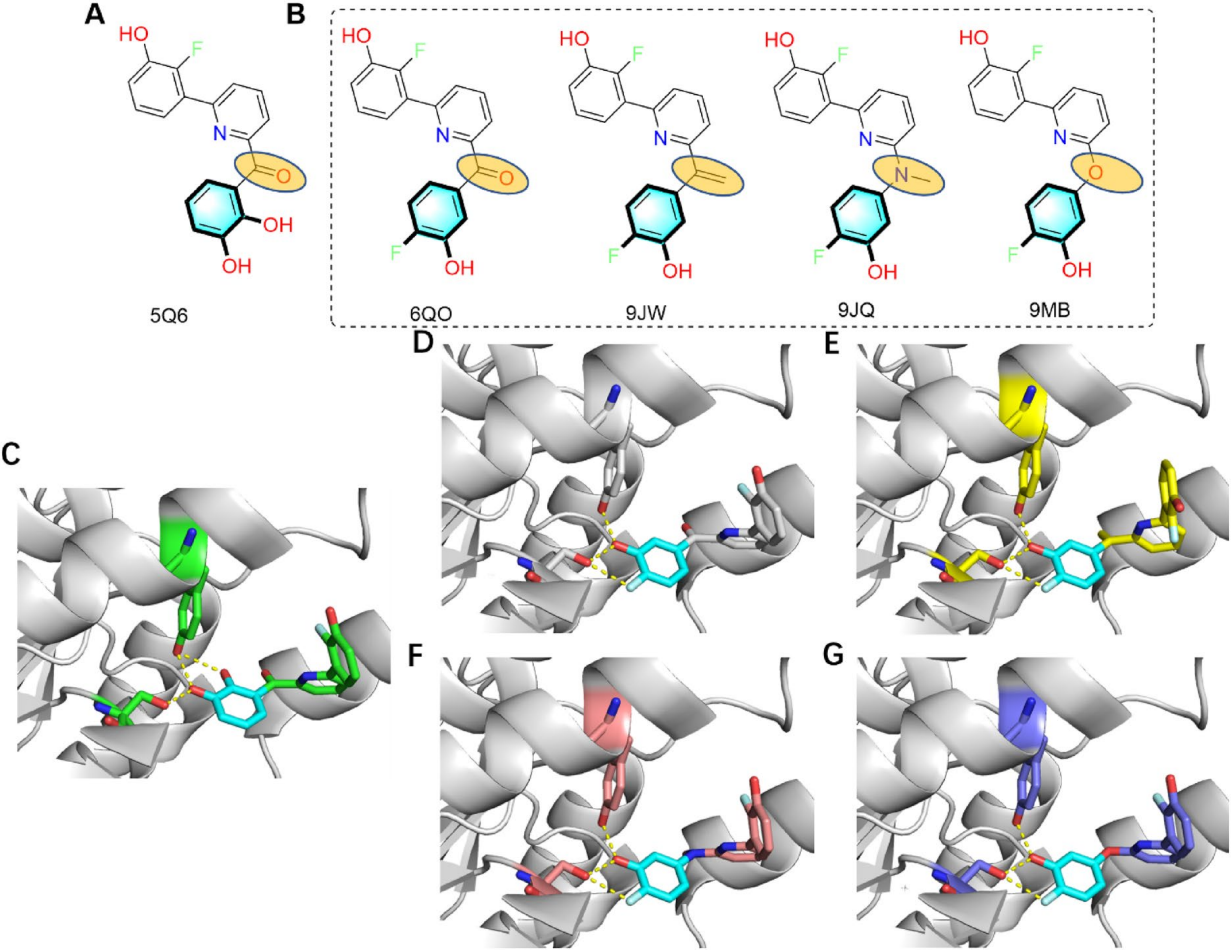
**A** Structure of 5Q6. **B** Four active 17β-HSD14 inhibitors suggested from a BII search. **C**–**G** Interaction networks between 6QO, 9JW, 9JQ and 9 MB and 17β-HSD14, respectively. In this figure, the ligands are named according to their PDB 3-lettercodes, and the proteins are named according to PDB4-lettercodes. Putative hydrogen bonds are shown as yellow dotted lines and the distance is labelled. The carbon atoms of structural replacements in the target ligand are highlighted in cyan, while others are shown in green, white, yellow, bronze and blue respectively

The human enzyme 17β-hydroxysteroid dehydrogenase 14 (17β-HSD14), using NAD^+^ as cofactor, oxidizes estradiol and 5-androstenediol. The human HSD17B14 gene is widely expressed in major organs, such as brain, liver and kidney. It has also been identified in breast cancer tissue, but the physiological function of this enzyme was poorly understood. The use of inhibitors can be important tools to study the physiological role of 17β-HSD14 in vivo. The methanone compound 1 (compound 12 in Ref. [46] PDB code: 5Q6) inhibits the activity of 17β-HSD14 with *K*_i_ of 64 nM. The hydroxyl residue of Tyr154 forms two hydrogen bonds bifurcately (d = 2.5 Å, d = 3.1 Å) with hydroxyl groups of the catechol moiety. Besides, the 4-OH hydrogen bond (d = 2.5 Å) also extends toward Ser141 hydroxyl residue (Fig. 11A). Four of 5Q6’s optional analogues are shown in Fig. 11B and suggested that 4-fluoro-3-phenol is the bioisostere of the 3-substituent catechol, offering a ligand (compound 9 in Ref. [46] PDB code: 6QO) with increased affinity (a Ki of 13 nM). The 3-OH groups at the C-ring of 9 and compound 12 in Ref. [46] interact through remarkably short H-bond interactions with the side chain of Tyr154 (9, d = 2.3 Å, 12, d = 2.5 Å) and the side chain of Ser141 (9, d = 2.5 Å, 12, d = 2.5 Å) from the catalytic triad. The 4-F group at the C-ring of 9 is possibly involved in forming a halogen bond (d = 2.8 Å) with Ser141 hydroxyl side reside. The 3-OH groups at the C-ring of 12 hydrogen bond toward the side chain of Tyr154 (d = 3.1 Å). The replacement of the ketone linker of compound 9 with ethenyl resulted in an eightfold more potent inhibitor (compound 5 in ref. PDB code: 9JW) with a *K*_i_ of 1.5 nM; while methylamine (compound 4 in ref. PDB code: 9JQ) and ether (compound 2 in reference PDB code: 9 MB) surrogate each individually deteriorated the binding affinity to a *K*_i_ of 42 and 58 nM. Keeping the B and C ring of 6QO unchanged, the equipotent quinoline base inhibitor (compound 9 in Ref. [47], PDB code: 9ME), and a two folds more active naphthalene derivative (compound 10 in Ref. [47]) were obtained, but the quinoline analog was found to be four times more soluble than the naphthalene compound. Herein, we rather than concentrate on the structural replacement of catechol, where it is replaced by a 4-fluoro-3-hydroxyphenyl moiety, instead emphasize that the linker connecting replacements to other parts can vary. However, it's crucial to acknowledge that the choice of linker may impact the physicochemical properties of the ligand.

### Comparison with other tools

The fundamental of isostere replacement lies matching of protein moieties, but sometimes this concept of replacement not aligned with the intended objective of functional group/ring/core replacement for a ligand. Therefore, BII was compared with other bioisosteric search tools, such as the SwissBioisostere database and the MolOpt network server. The SwissBioisostere database is a comprehensive resource containing information about molecular substitutions and their performance in biochemical analysis. This data is obtained by matching molecular pairs and mining biological activity data from the ChEMBL database. Notably, SwissBioisostere not only provides information about molecular substitutions but also offers interactive analysis capabilities. On the other hand, the MolOpt network server is constructed through a combination of data mining, chemoinformatics similarity comparison, and machine learning techniques. Users have the flexibility to query for bioisosteres of specific molecular substructures and even generate entirely new molecular alternatives.

**Fig. 12:**
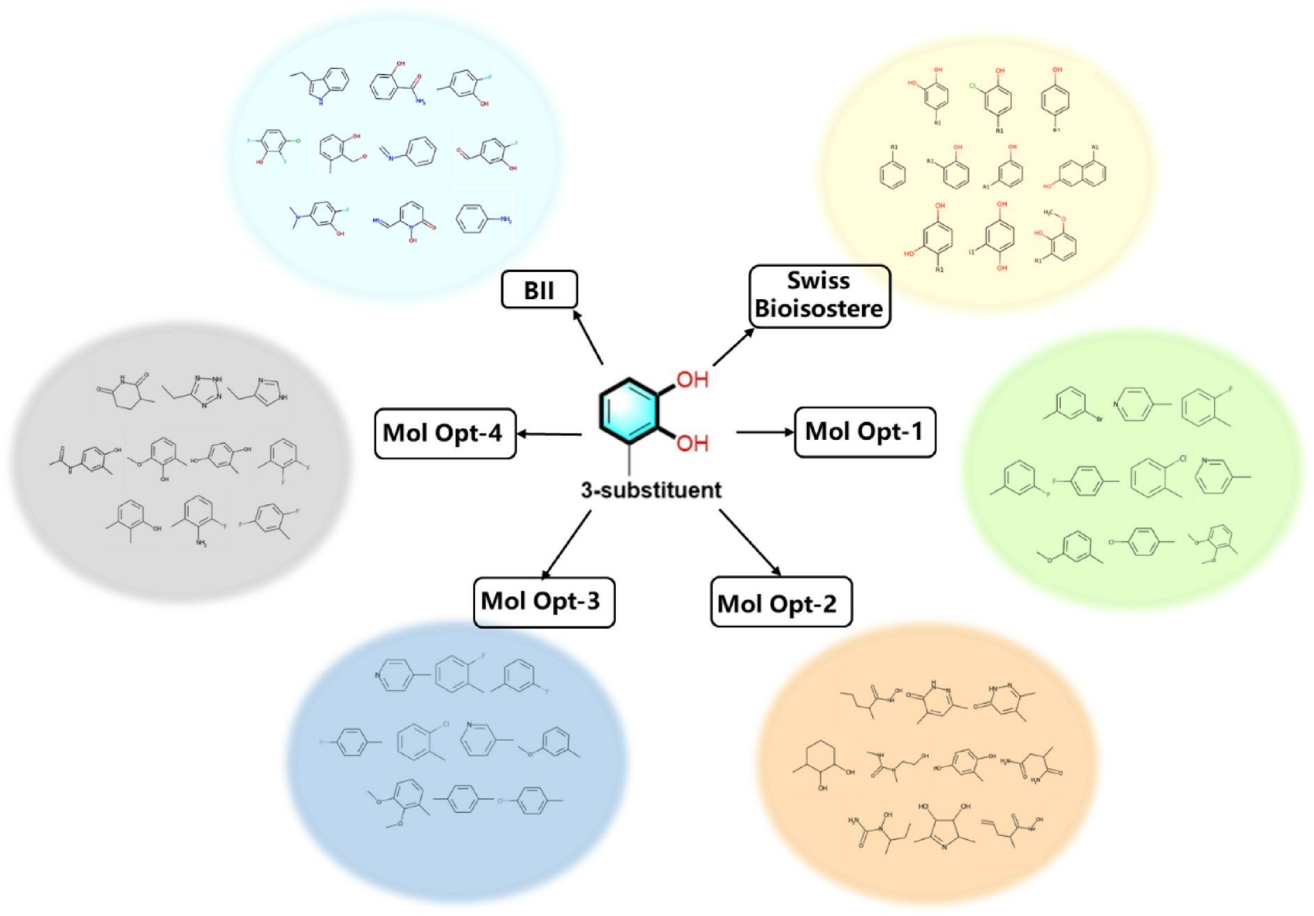
Top-10 ranked bioisosteres of 3-substituent catechol suggested by different tools

To perform a comparative analysis, three distinct substructures, namely the 3-substituent, 4-substituent, and 3,4-substituent, were input into each of the three search tools. Consequently, users can access the corresponding bioisosteric data for their chosen substructures. In Table 1, we have summarized the number of bioisosteres identified by SwissBioisostere, MolOpt, and BII. Additionally, it's important to note that MolOpt offers four distinct bioisosteric replacement rules. MolOpt-1 is based on data mining principles, MolOpt-2 utilizes similarity comparison, MolOpt-3 incorporates data mining techniques, and MolOpt-4 is designed around a deep generative model. It becomes evident that when compared to the SwissBioisostere database and the MolOpt web server, BII excels in providing a more extensive array of bioisosteric ideas, making it a valuable resource for medicinal chemistry research. The bioisosteres with the top-ten rankings from each tool are depicted in Fig. 12, illustrating consistent results. The chemical accessibility represents an important concern indeed for the novel structure generated based on this tool, but we want to emphasize that BII focus on local structural replacements yet did not consider how to incorporate suggested moieties into new ligands, but definitely it will be put into consideration as a filter of replacement moieties in updated BII version. In addition, we recognized that a retrospective validation is not satisfactory to launch BII since experimental validation in any case is a benchmark of computational tool. In fact, we conducted both wet lab synthetic and bioassay experiments in-house. It has been demonstrated that a squaryldiamide or an amide group is the bioisosteric replacement of phosphate moiety [48], NH in the urea serves as isostere of carboxylic acid [49]. After previous computational investigation of phosphate [31], ribose [32] bioisosteric replacement, the bioisosterism of these moieties have been verified. Consequently, we think it is necessitated to develop a generic tool to facilitate bioisostere identification of any chemical fragment, which pillars the basement of our current attempt.

**Table 1:** Comparison of query results of different search platforms

Target functional group	The number of bioisosteres found by different search tools					
	SwissBioisostere	MolOpt-1	MolOpt-2	MolOpt-3	MolOpt-4	BII
3-substituent	161	100	200	121	200	496
4-substituent	631	100	200	200	200	2559
3,4-substituent	56	9	200	9	200	3322

## Conclusions

To optimize the efficiency of BII, we integrated the extended multiprocessing library of Python into the code. BII stands out as a user-friendly and robust tool for generating innovative ligand replacement ideas. The substructure replacement identification process for a specific single task typically takes about two to eleven hours using a machine with a CPU of 24 processors. Notably, the web server is designed to be accessible without the need for computational or programming skills, a feature particularly advantageous for medicinal chemists. These results affirm BII’s capability to identify suitable LSR where the chemical structure differs, yet the interaction patterns with the protein pocket remain conserved. Moreover, our application of BII has led to the rediscovery of scaffold hopping ideas, underscoring the utility of our web server in providing valuable insights for ligand design. In essence, BII serves as a valuable tool to assist medicinal chemists during the hit/lead optimization process, aiding in the search for appropriate molecular fragments. As part of our commitment to ongoing improvement, the BII server will receive regular updates as new data and advancements become available. We are pleased to offer this service freely to the public at http://www.aifordrugs.cn/index/.

### Supplementary Information

**Additional file 1:** **S1**.'batch_download.sh' # Python script to download the PDB database code: **S2**. Taking 3-substituent as the target functional group, the bioelectronic isoplatoon was searched in BII, and the results were as follows, a total of 50 pages of data. **S3**. The LSR subgroup of 4-substituent catechol categorized as cycle C+O+N. **S4** The LSR subgroup of 3,4-substituent catechol categorized as cycle C+O+N. **S5** Visualization of the data clustering.

## Data Availability

The focus of our manuscript is on the online webserver development computational to identify local structural replacements/bioisosteres for drug design. ChemDraw 19.0 was used to sketch the structure of ligands. The PyMoL 1.8.x used in this work to visualize and demonstrate the interactions between ligand and receptor is free and open-source software. All code, data and deployment environments for this work have been uploaded to Zeodo and can be accessed via the following link: https://doi.org/10.5281/zenodo.8215113.
