# Supplementary material for: BioisoIdentifier: an online free tool to investigate local structural replacements from PDB
Source: J Cheminform. 2024 Jan 13;16:7. doi: 10.1186/s13321-024-00801-8 (PMC10788035; doi:10.1186/s13321-024-00801-8)
Supplement: Supplementary file 1 — Additional file 1: S1.'batch_download.sh' # Python script to download the PDB database code: S2. Taking 3-substituent as the target functional group, the bioelectronic isoplatoon was searched in BII, and the results were as follows, a total of 50 pages of data. S3. The LSR subgroup of 4-substituent catechol categorized as cycle C+O+N. S4 The LSR subgroup of 3,4-substituent catechol categorized as cycle C+O+N. S5 Visualization of the data clustering. [file 13321_2024_801_MOESM1_ESM.docx]

BioisoIdentifier: An Online Free Tool to Investigate Local Structural Replacements from PDB

Tinghao Zhang,^a,⊥^ Shaohua Sun,^a,⊥^ Runzhou Wang,^b^ Ting Li,^a^ Bicheng Gan,^c^ and Yuezhou Zhang*^a, d^

1. Xi’an Institute of Flexible Electronics (IFE) and Xi’an Institute of Biomedical Materials & Engineering (IBME), Northwestern Polytechnical University, 127 West Youyi Road, Xi'an, 710072, China.
2. School of Management, Xi’an University of Architecture and Technology, Xi’an 710055, China.
3. College of Petroleum Engineering, Northeast Petroleum University, Heilongjiang, Daqing 163318, China.
4. Ningbo Institute of Northwestern Polytechnical University, Frontiers Science Center for Flexible Electronics (FSCFE), Key laboratory of Flexible Electronics of Zhejiang Province, Ningbo Institute of Northwestern Polytechnical University, 218 Qingyi Road, Ningbo, 315103, China.

^⊥^ These authors are equally contributed to this work.

Email: iamyzzhang@nwpu.edu.cn (Y. Zhang)

S1.batch_download.sh # Python script to download the PDB database

code:

#!/bin/bash

# Set the following PDB_ID_FILE variable to the path to your PDB ID list file

PDB_ID_FILE="pdb_ids.txt"

# Check that curl is installed

if ! command -v curl &> /dev/null; then

echo " The curl tool is not installed. Please install curl to continue."

exit 1

fi

# Reads a comma-separated list of PDB IDs from the file

IFS=',' read -ra PDB_IDS < "$PDB_ID_FILE"

# Download each PDB file

for PDB_ID in "${PDB_IDS[@]}"; do

PDB_ID="$(echo "${PDB_ID}" | tr '[:upper:]' '[:lower:]' | tr -d '[:space:]')"

echo " Downloading PDB ID: ${PDB_ID}"

curl -fsSL "https://files.rcsb.org/download/${PDB_ID}.pdb" -o "${PDB_ID}.pdb"

done

echo " All PDB files are downloaded."

S2. Taking 3-substituent as the target functional group, the bioelectronic isoplatoon was searched in BII, and the results were as follows, a total of 50 pages of data.
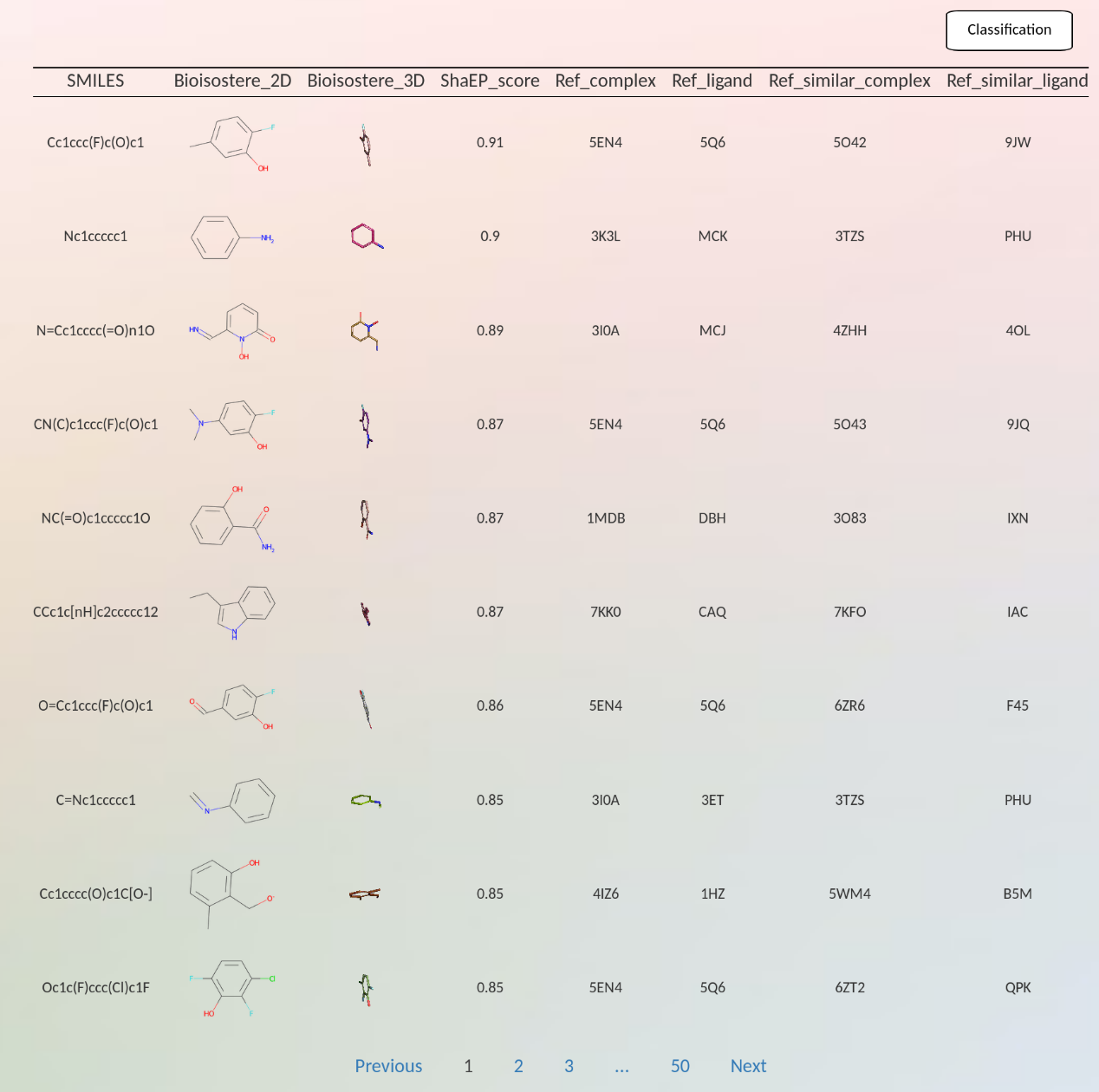

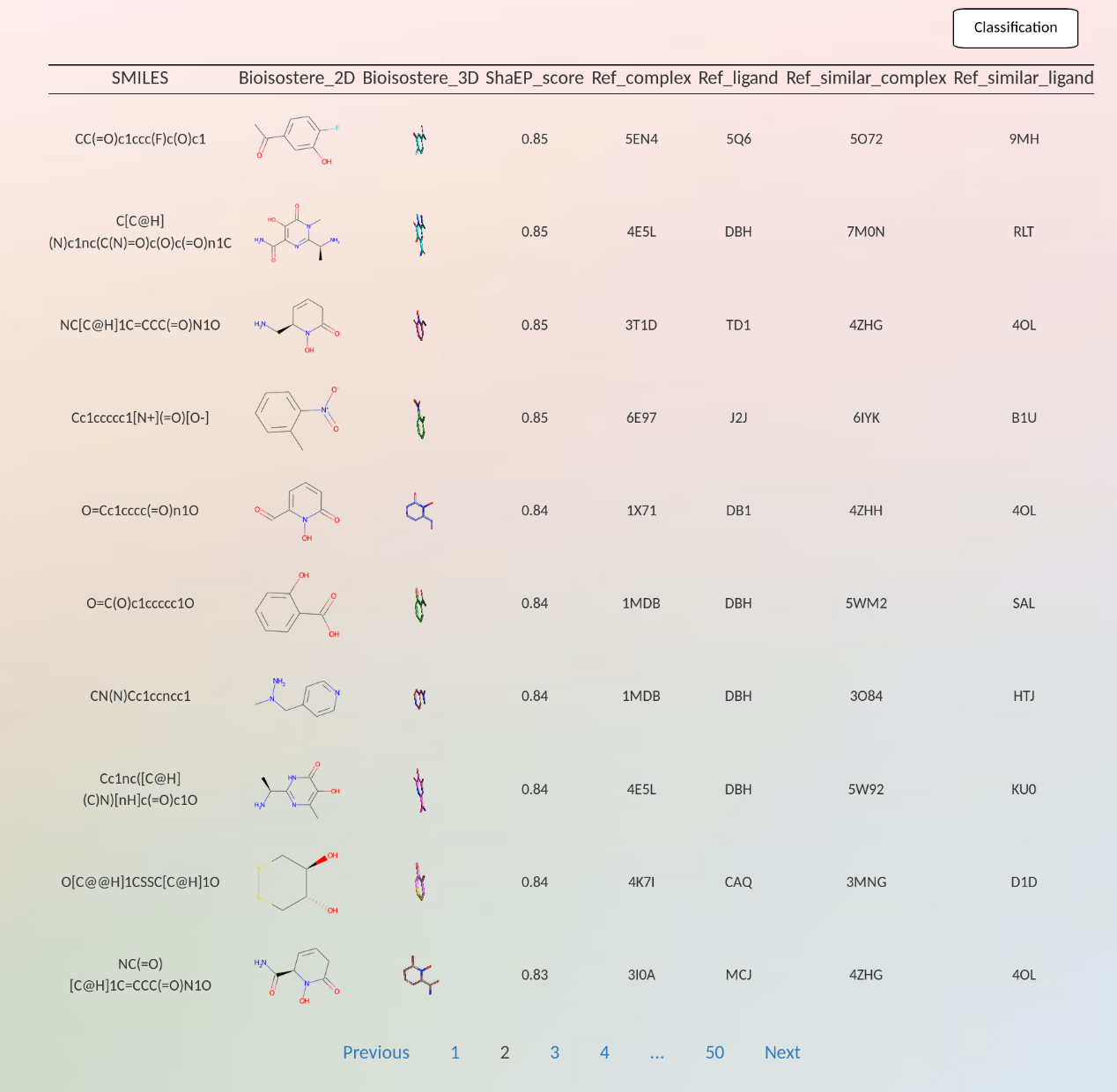

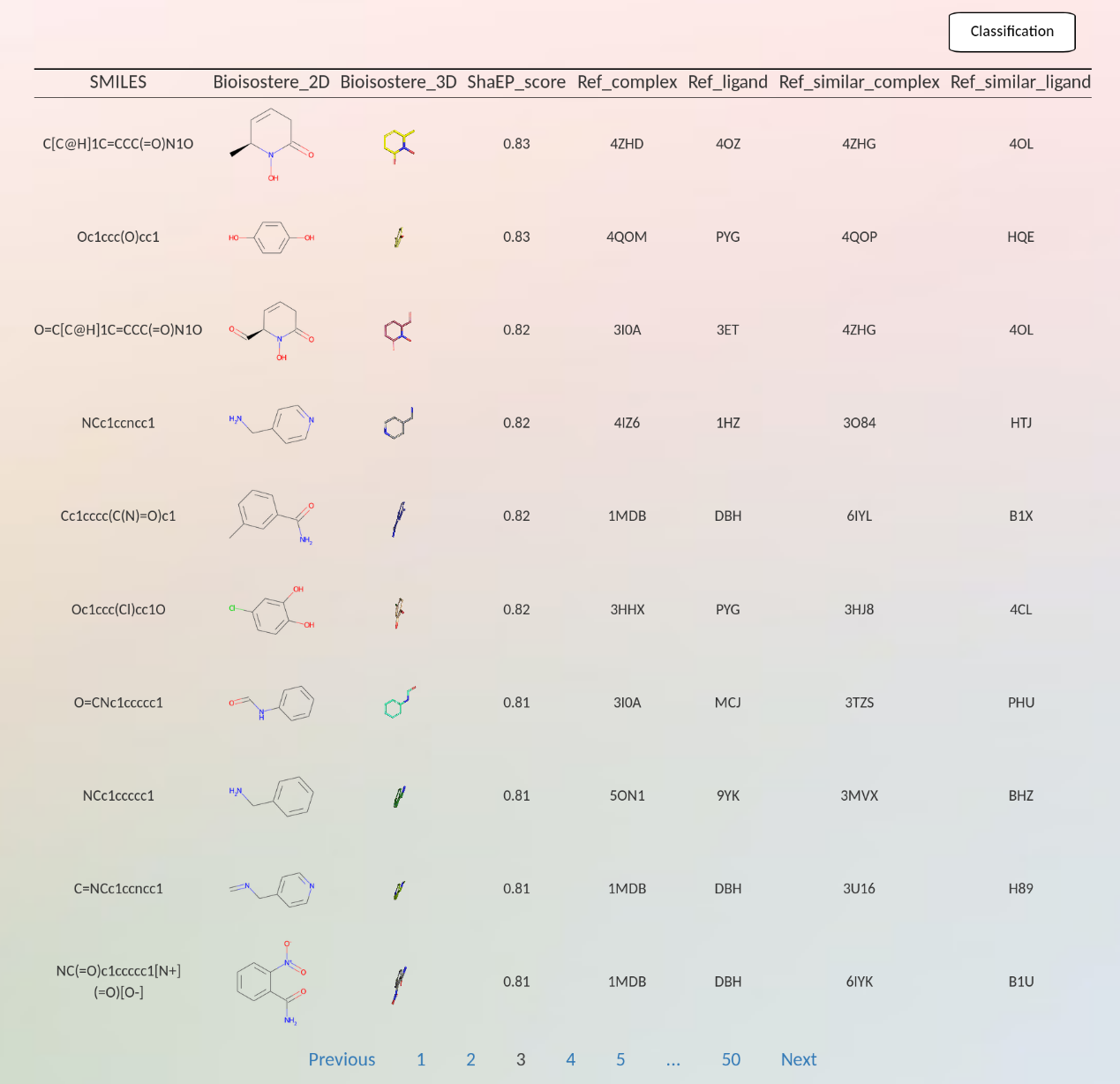

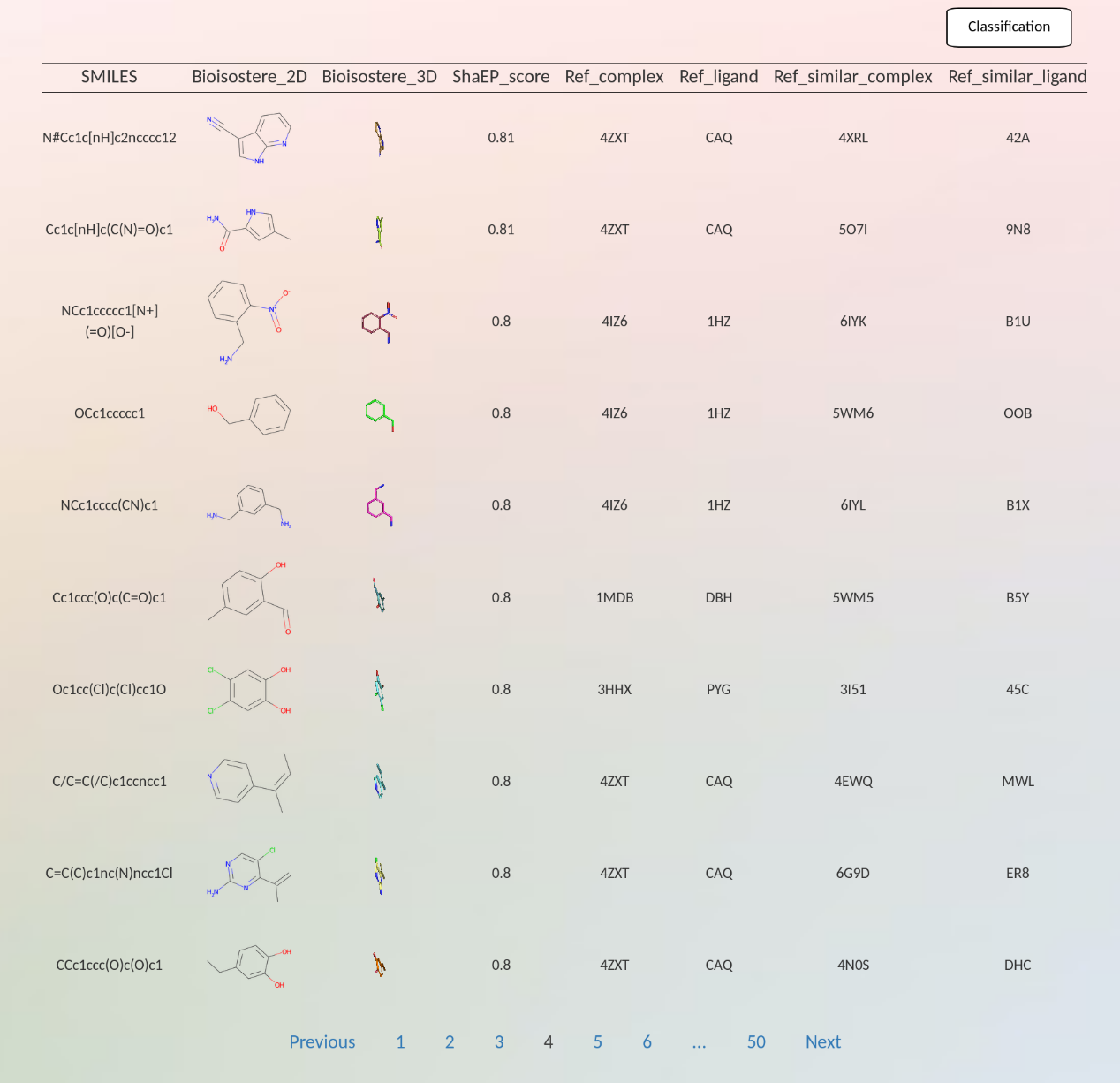

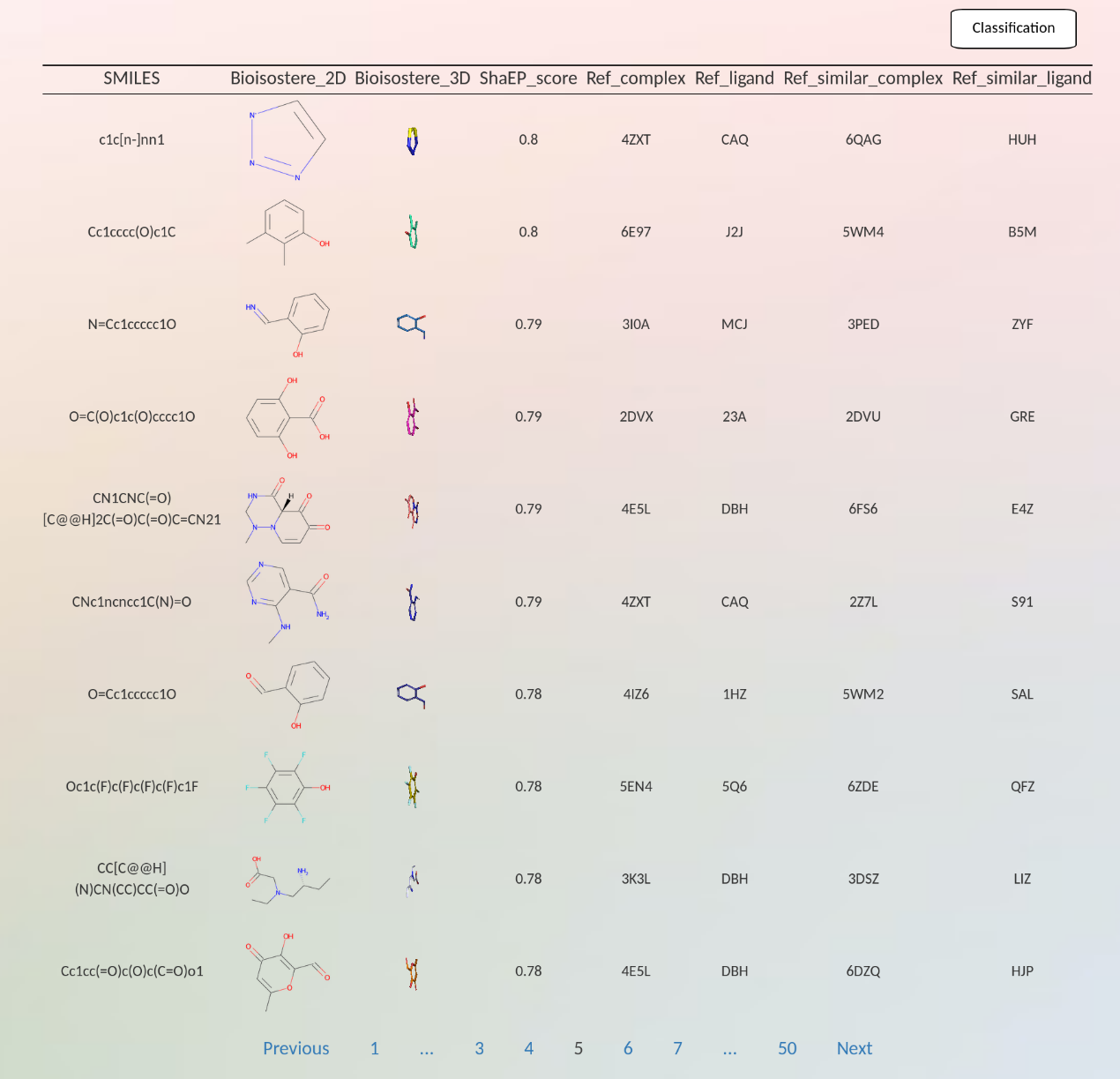

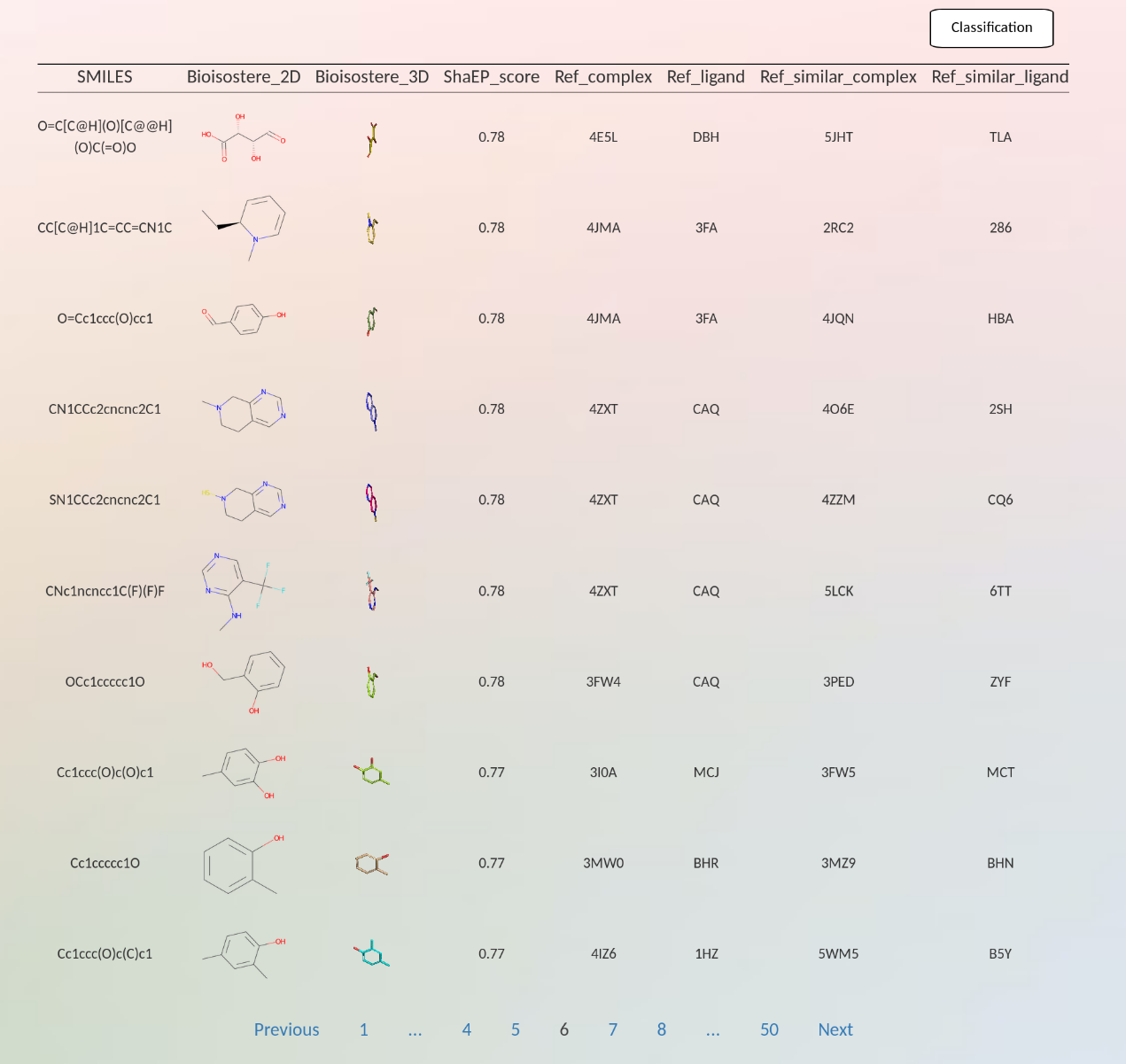

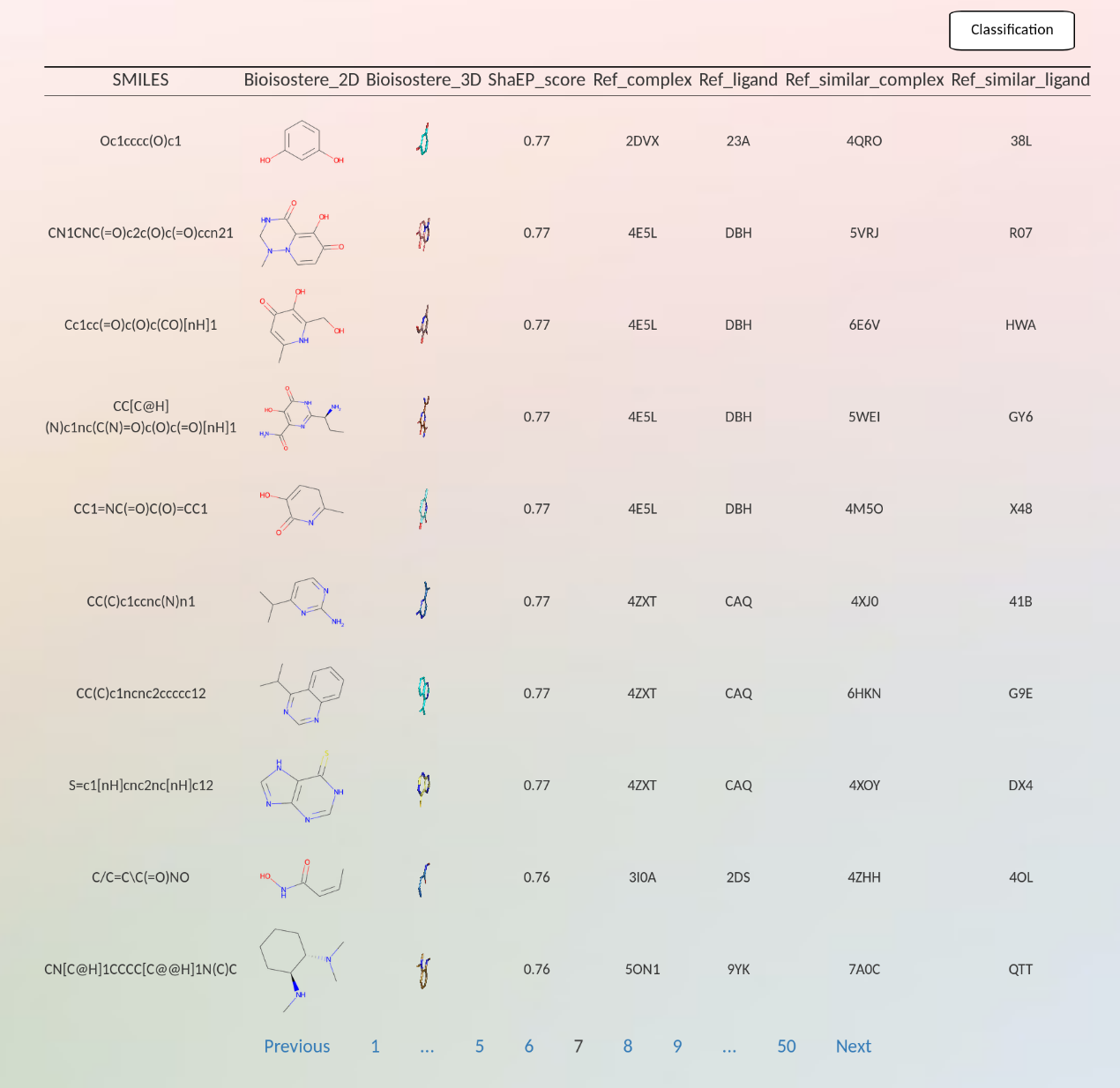

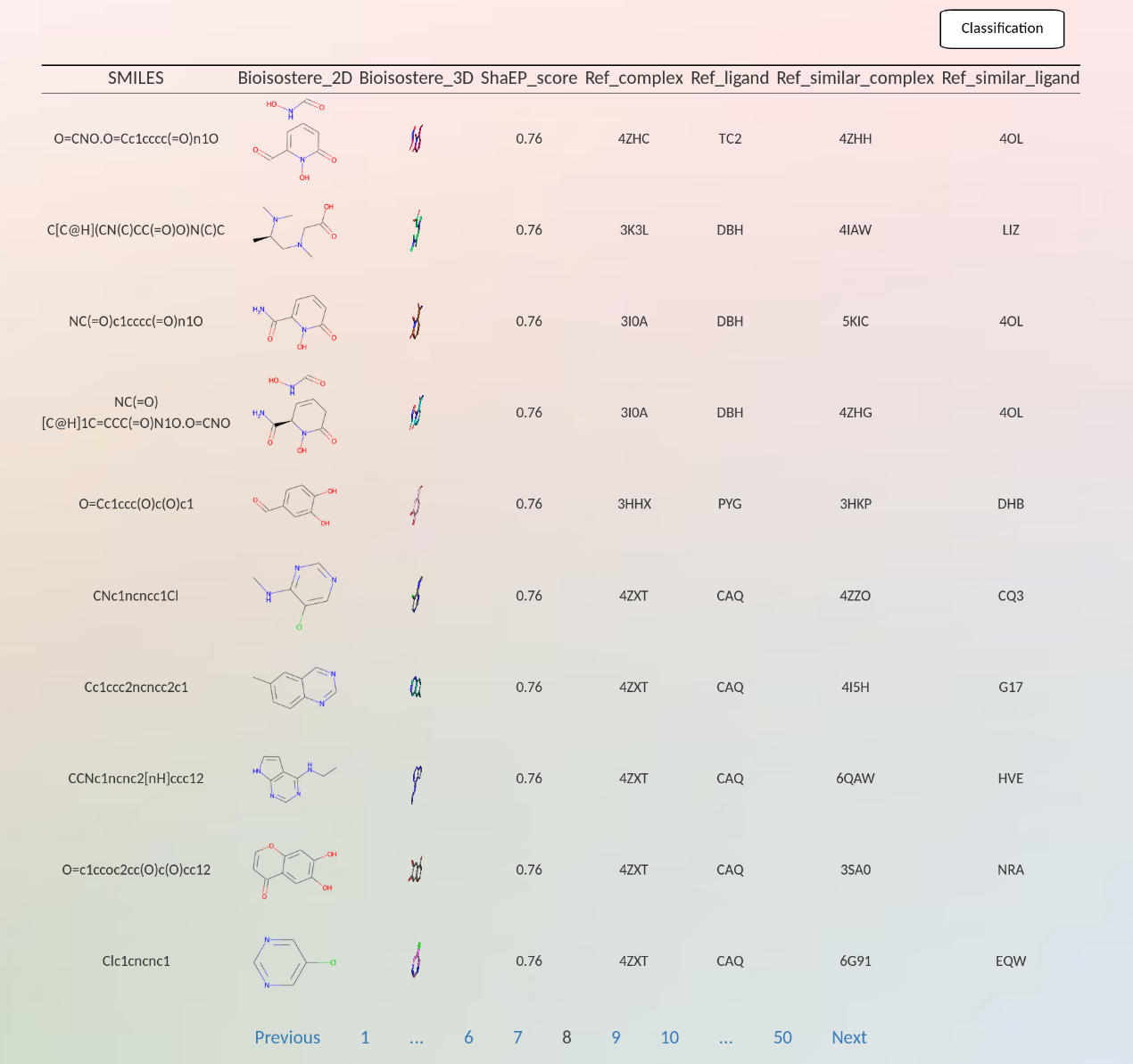

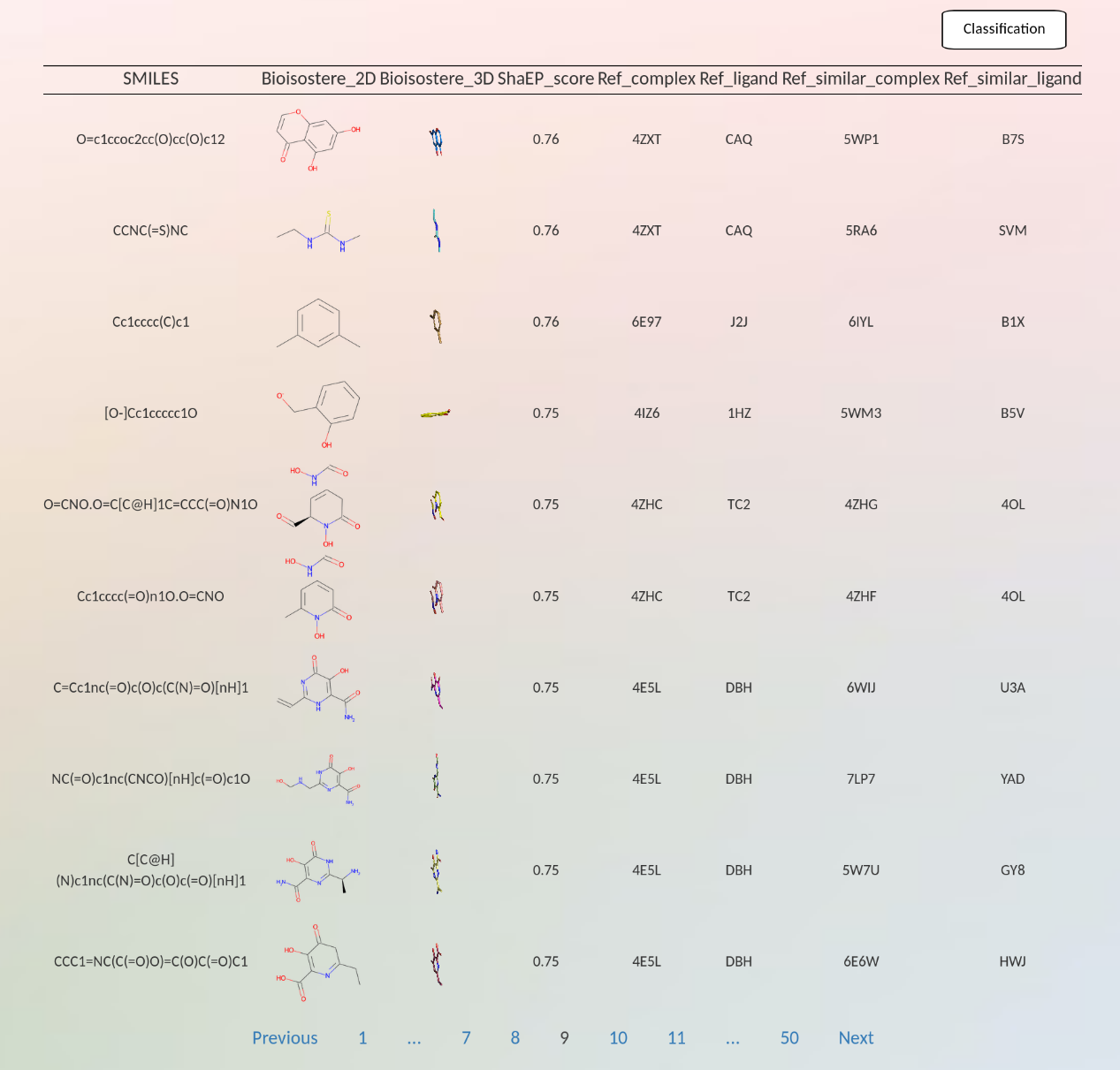

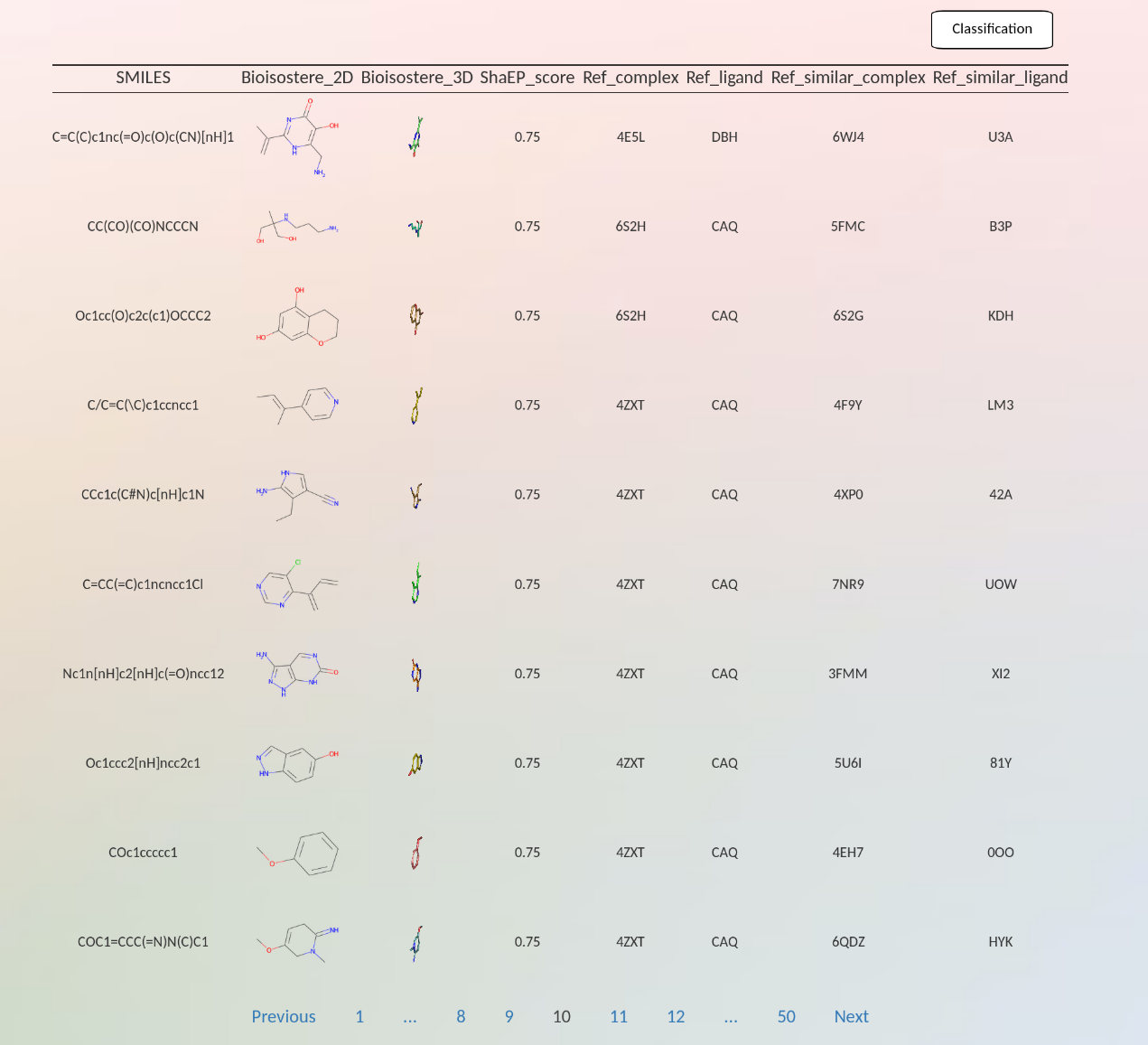

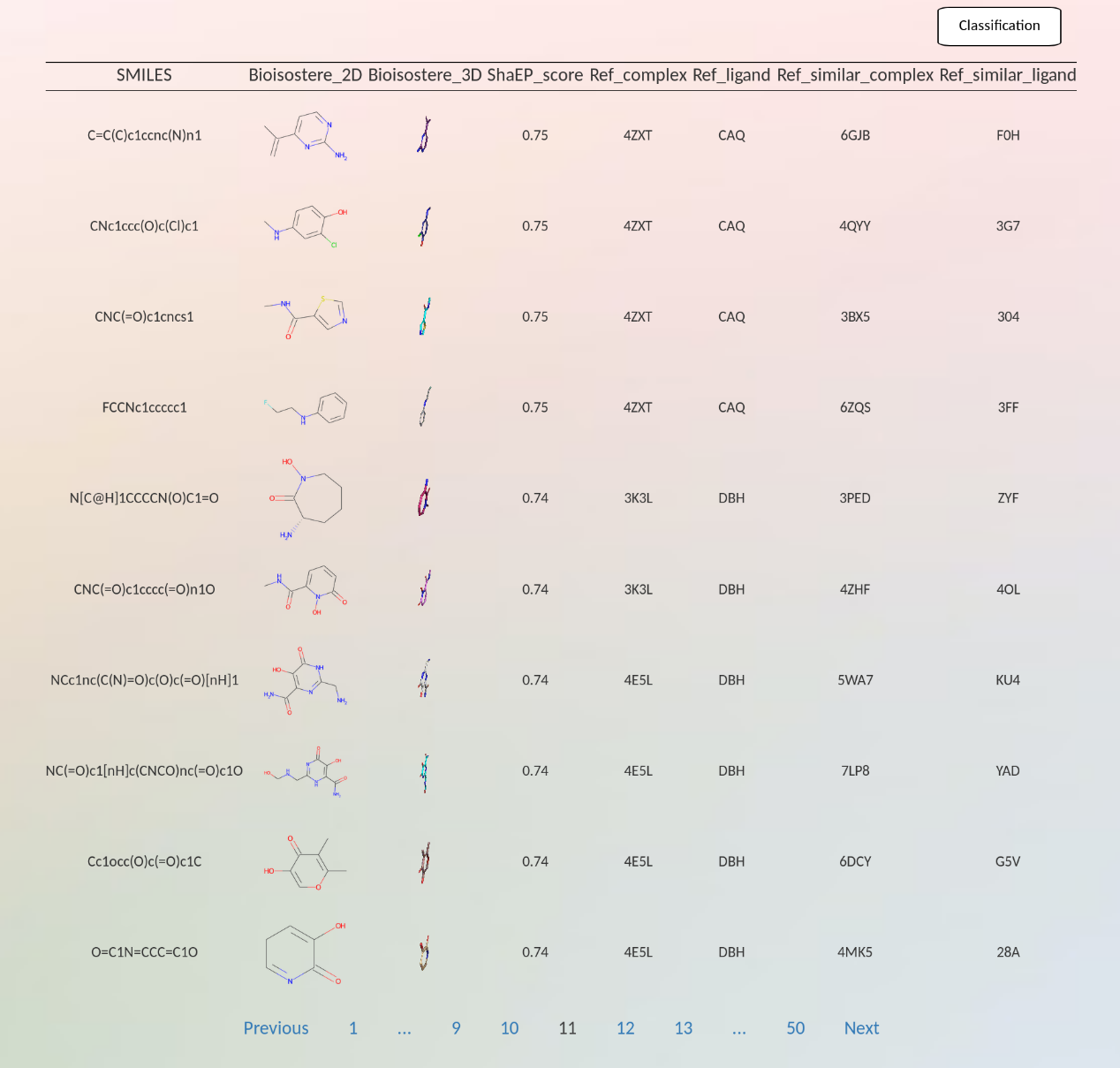

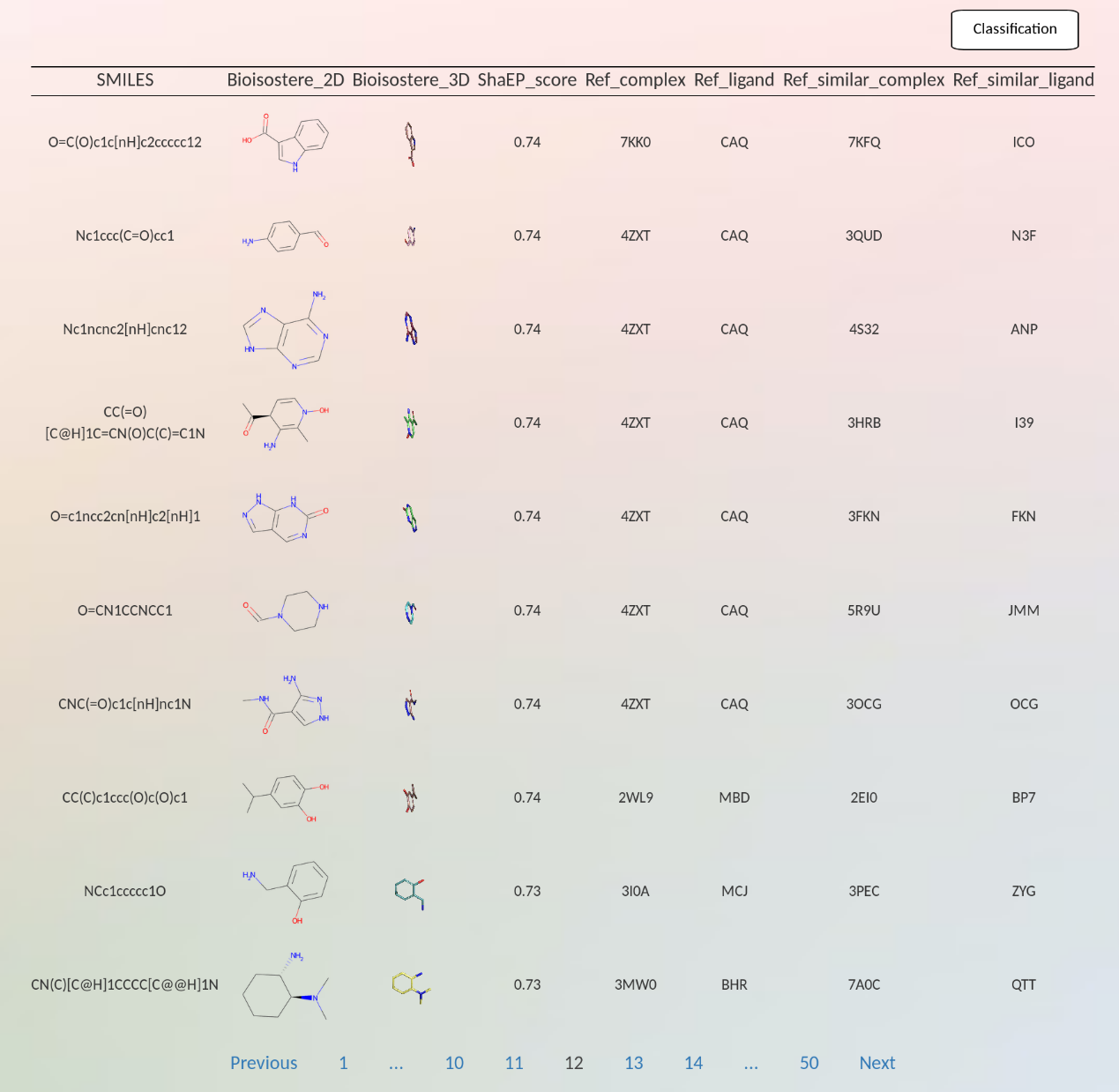

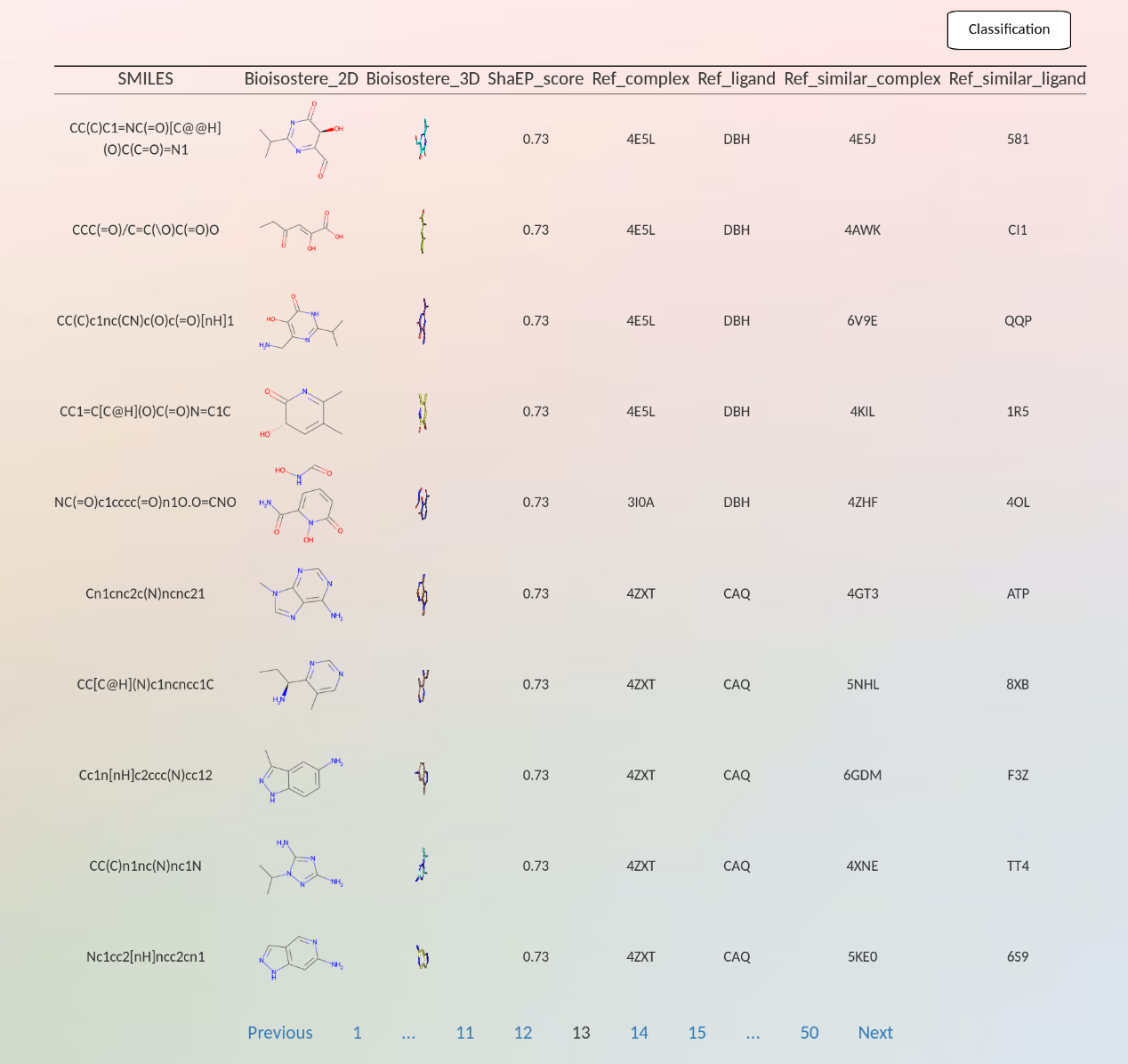

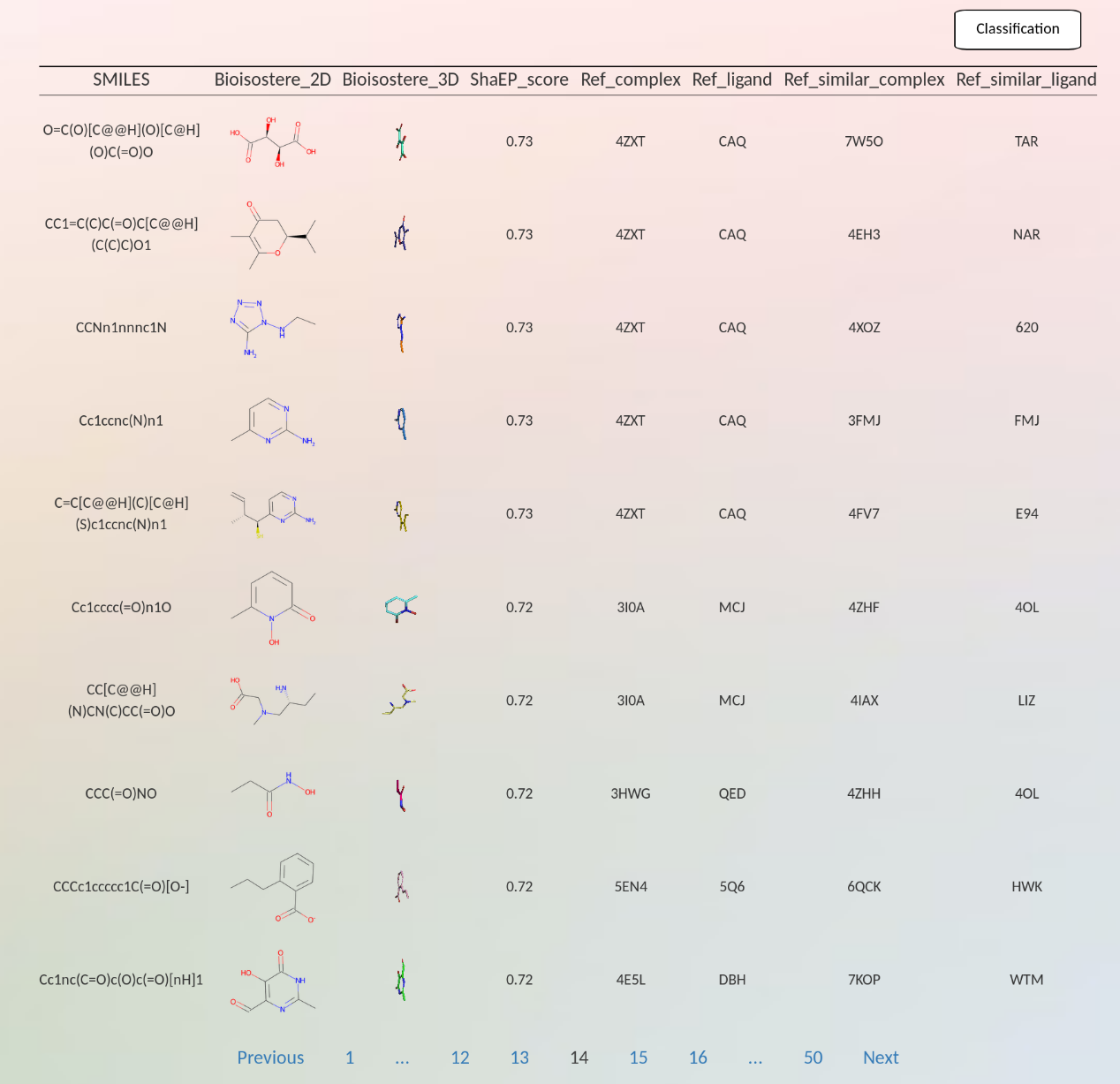

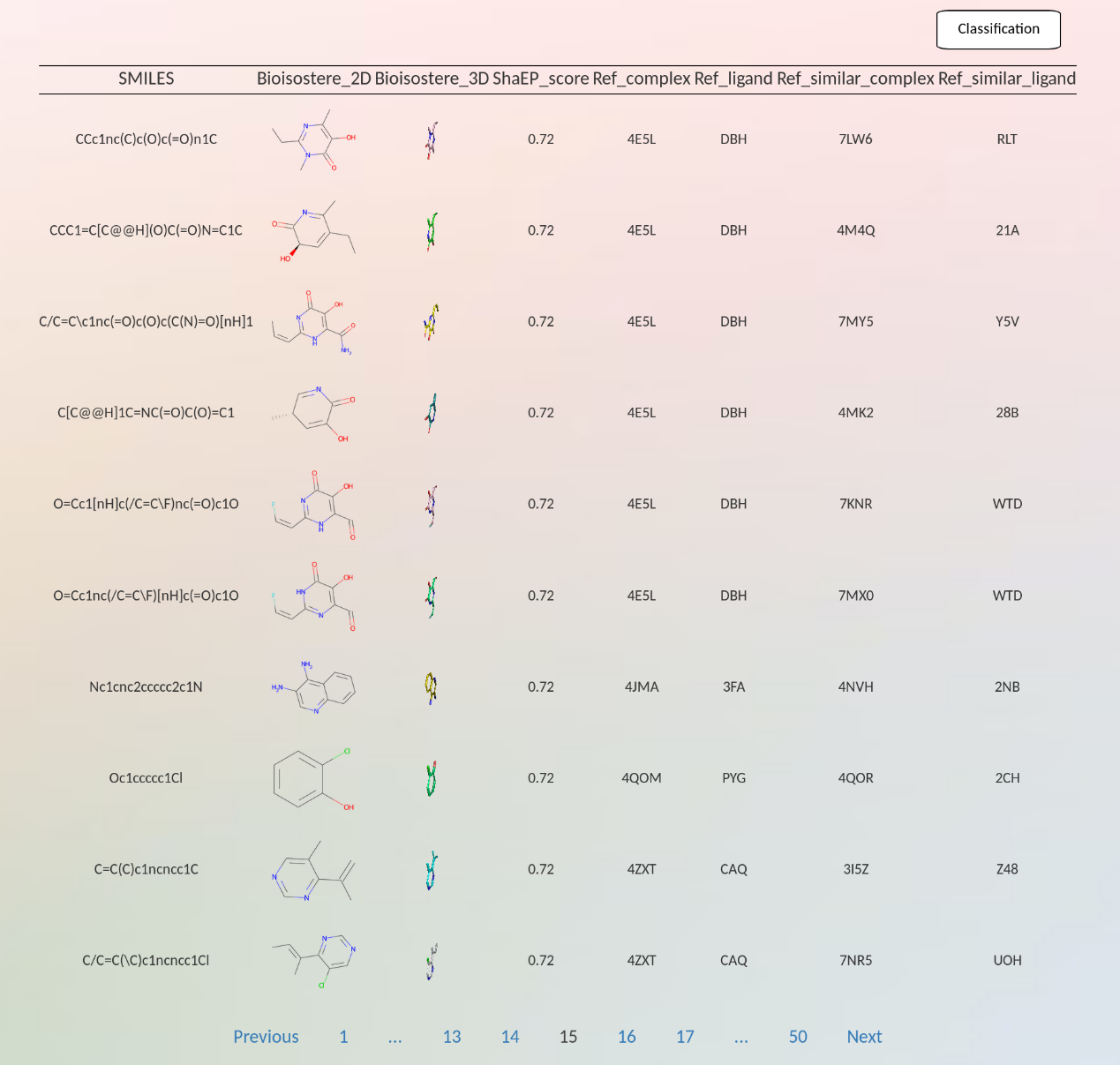

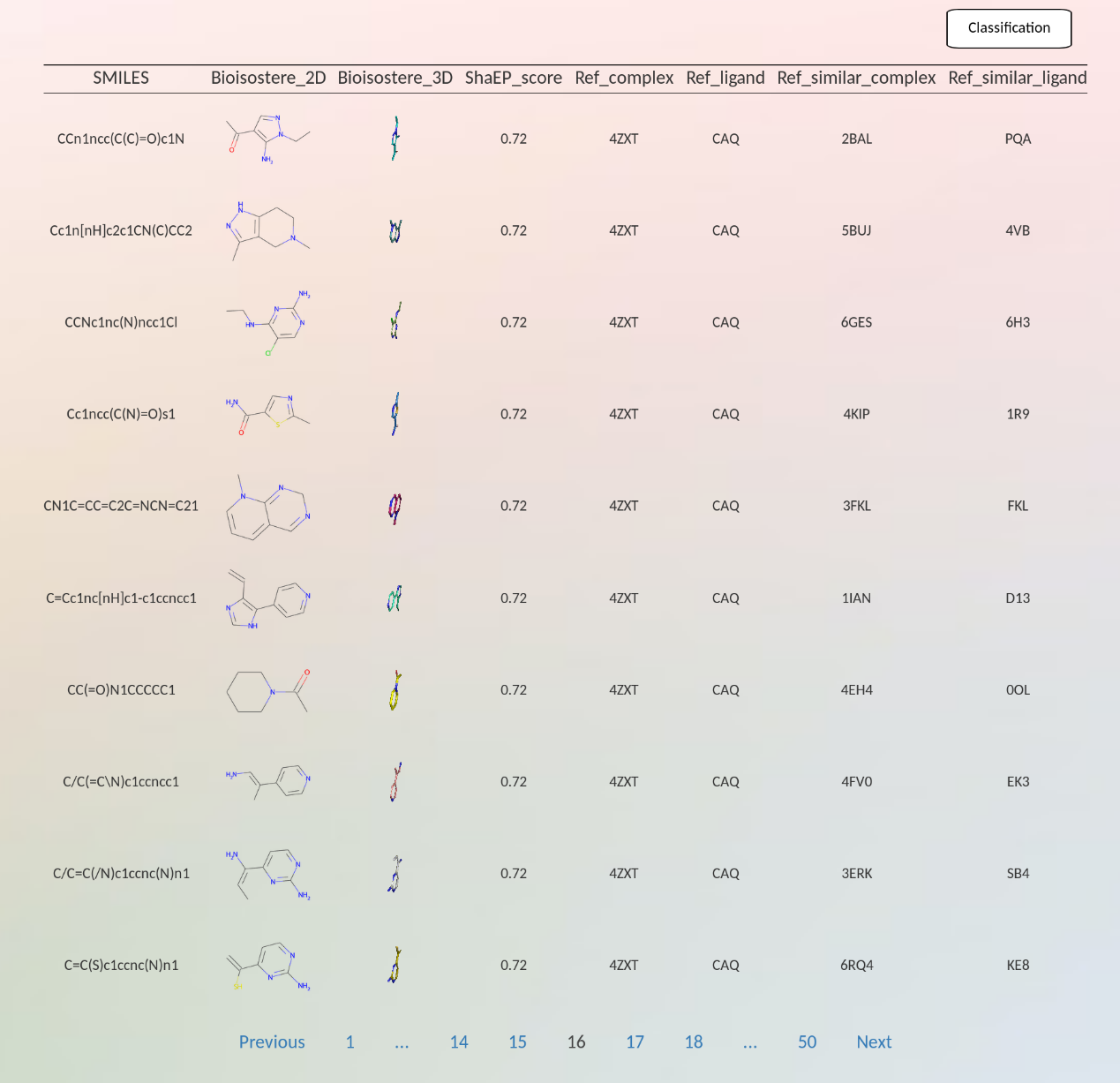

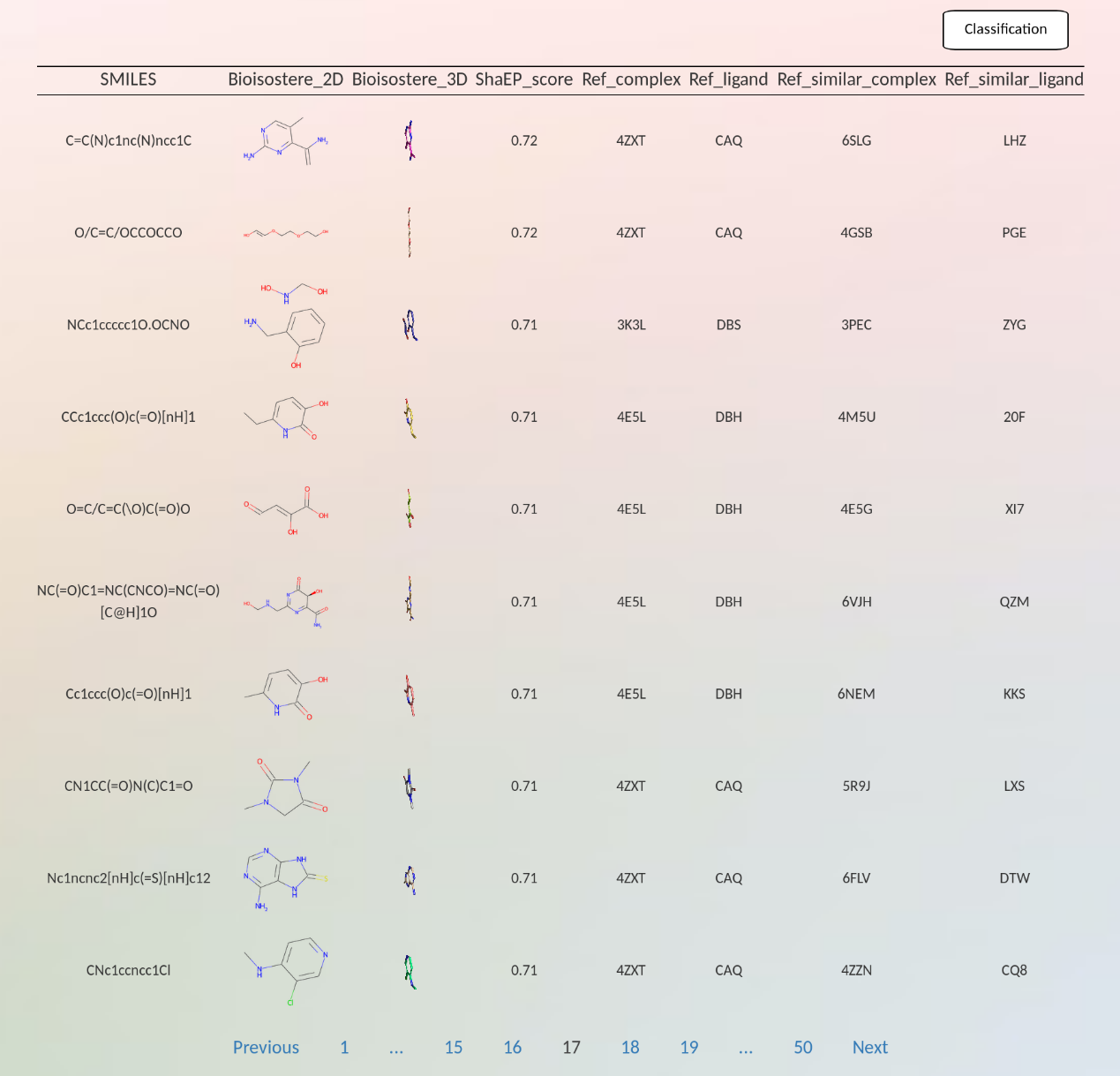

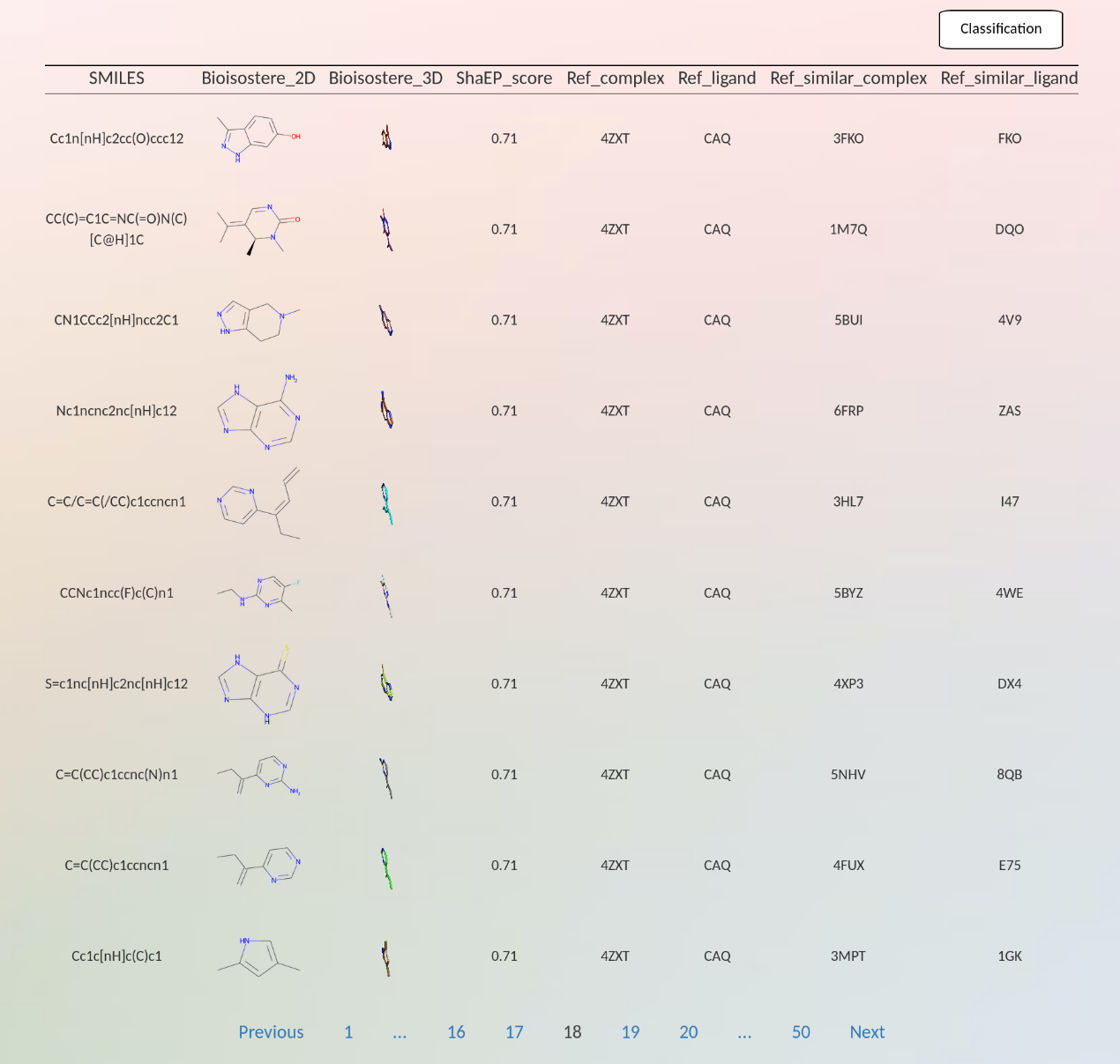

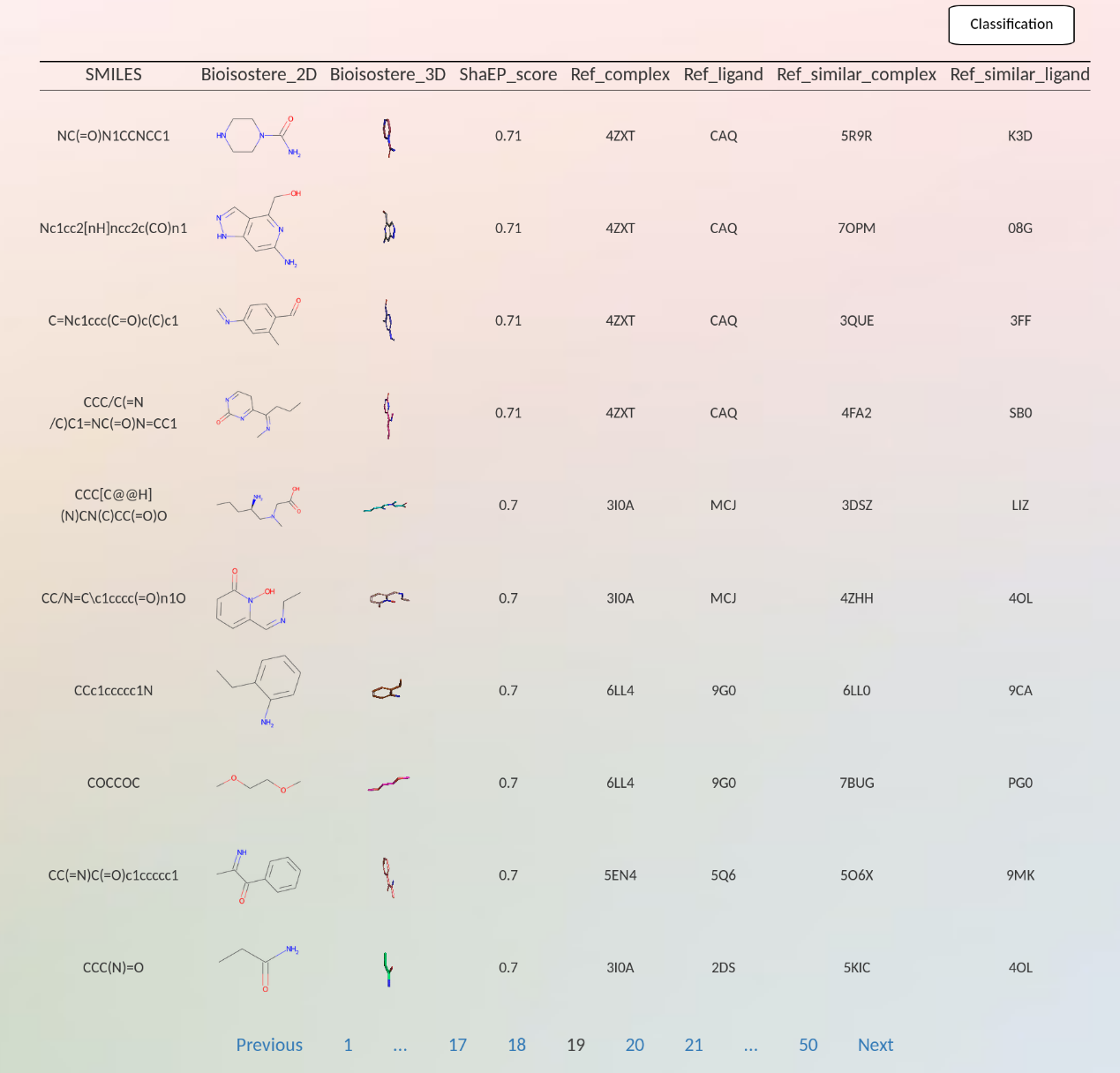

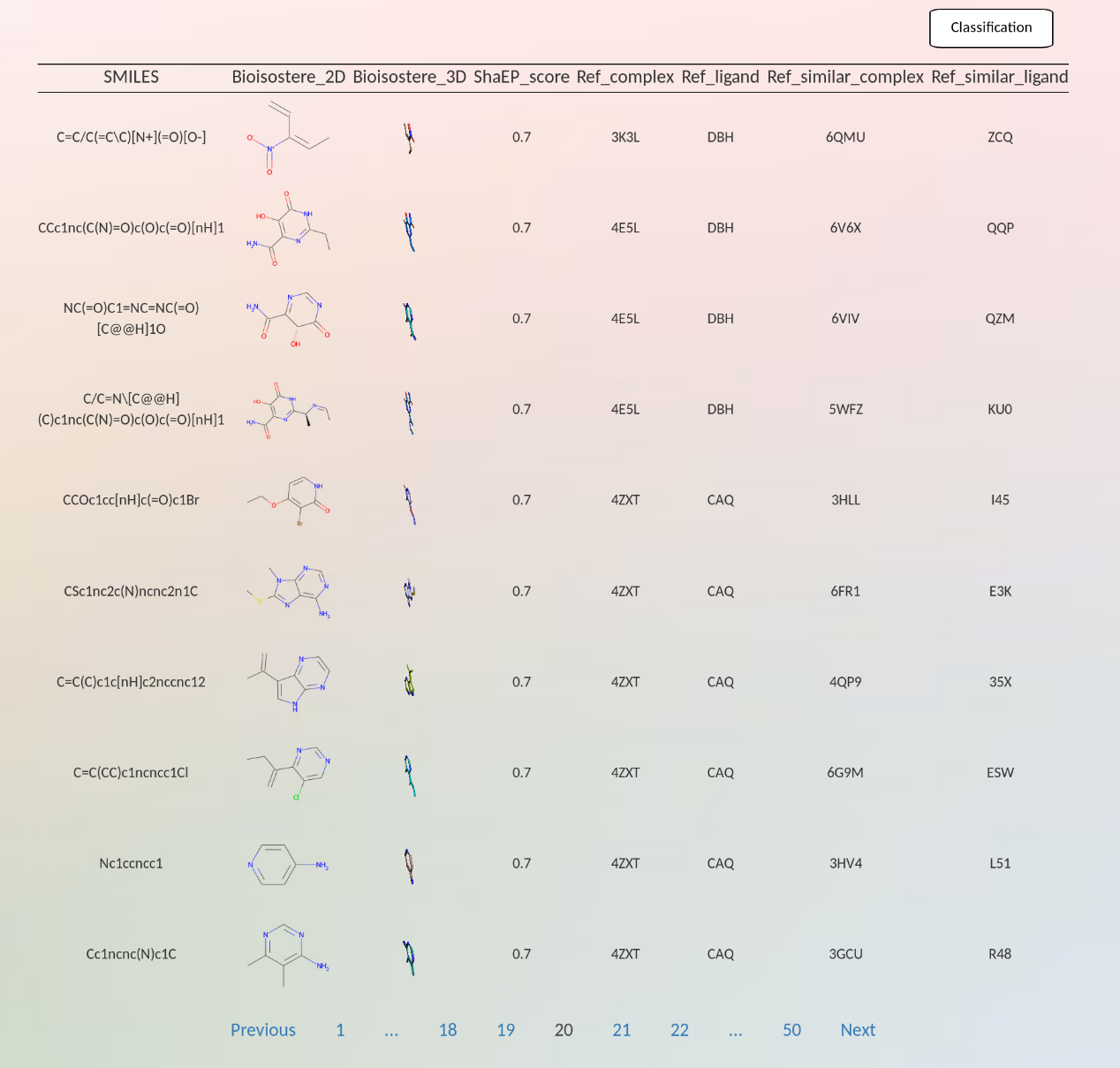

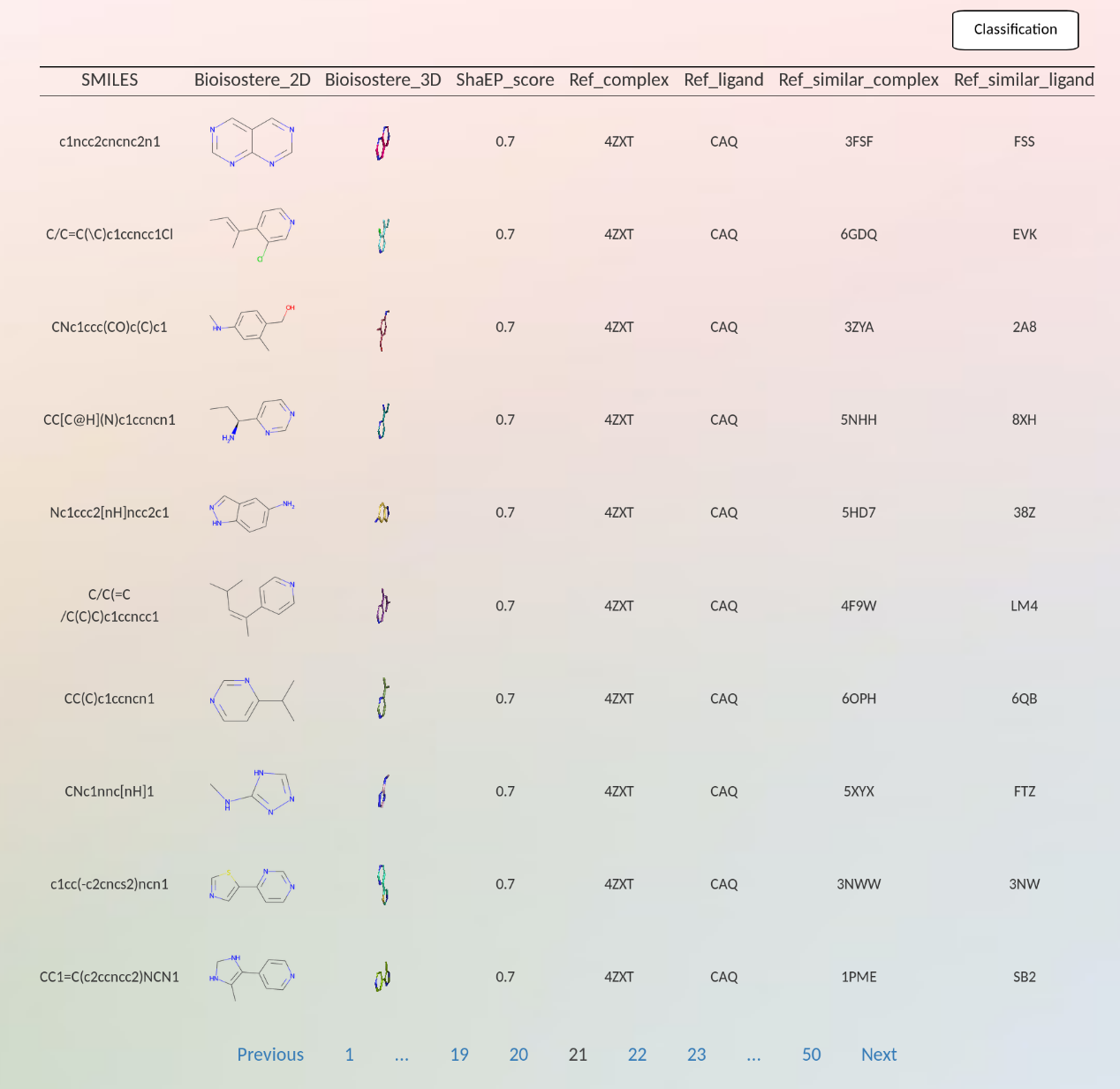

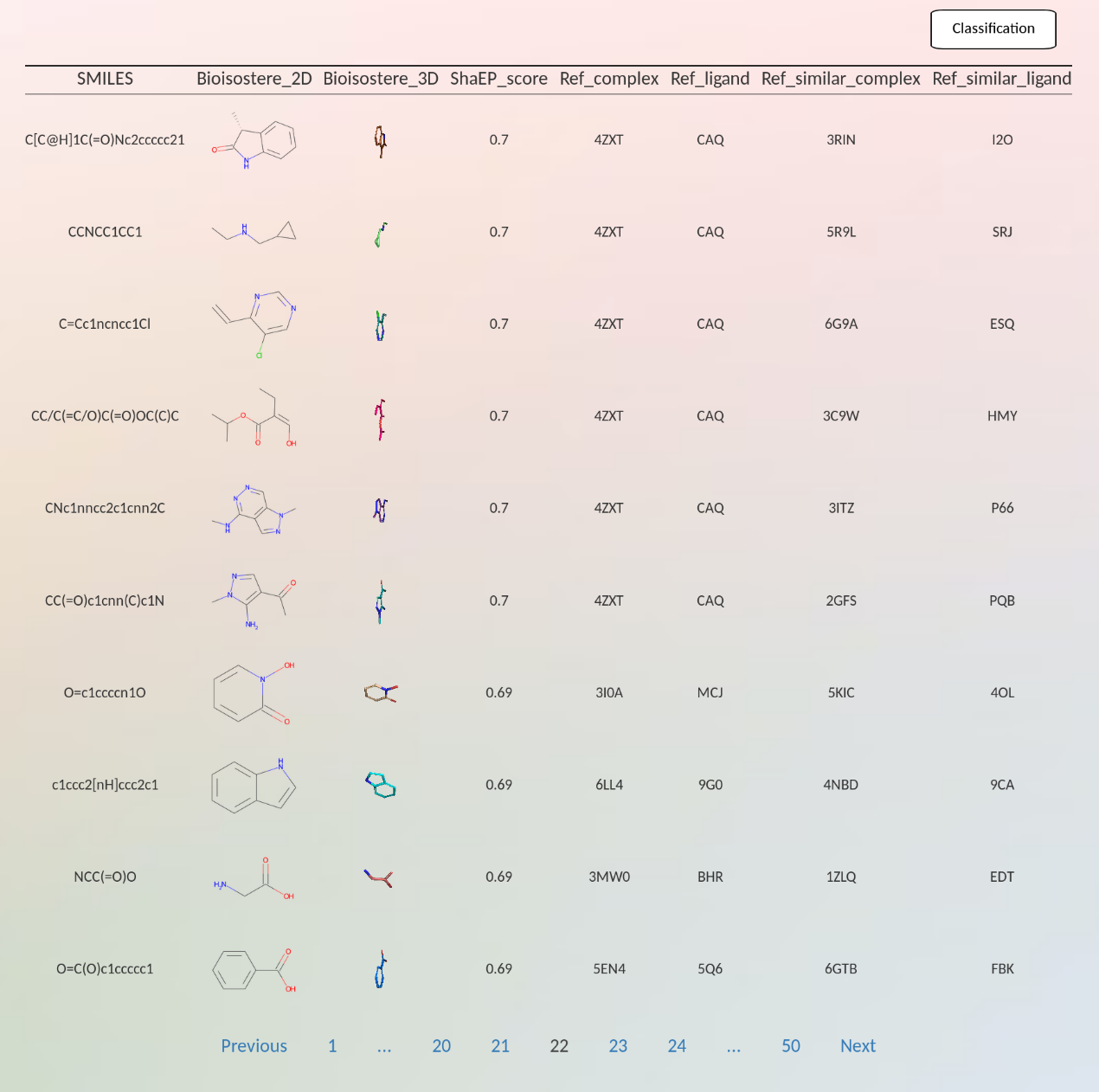

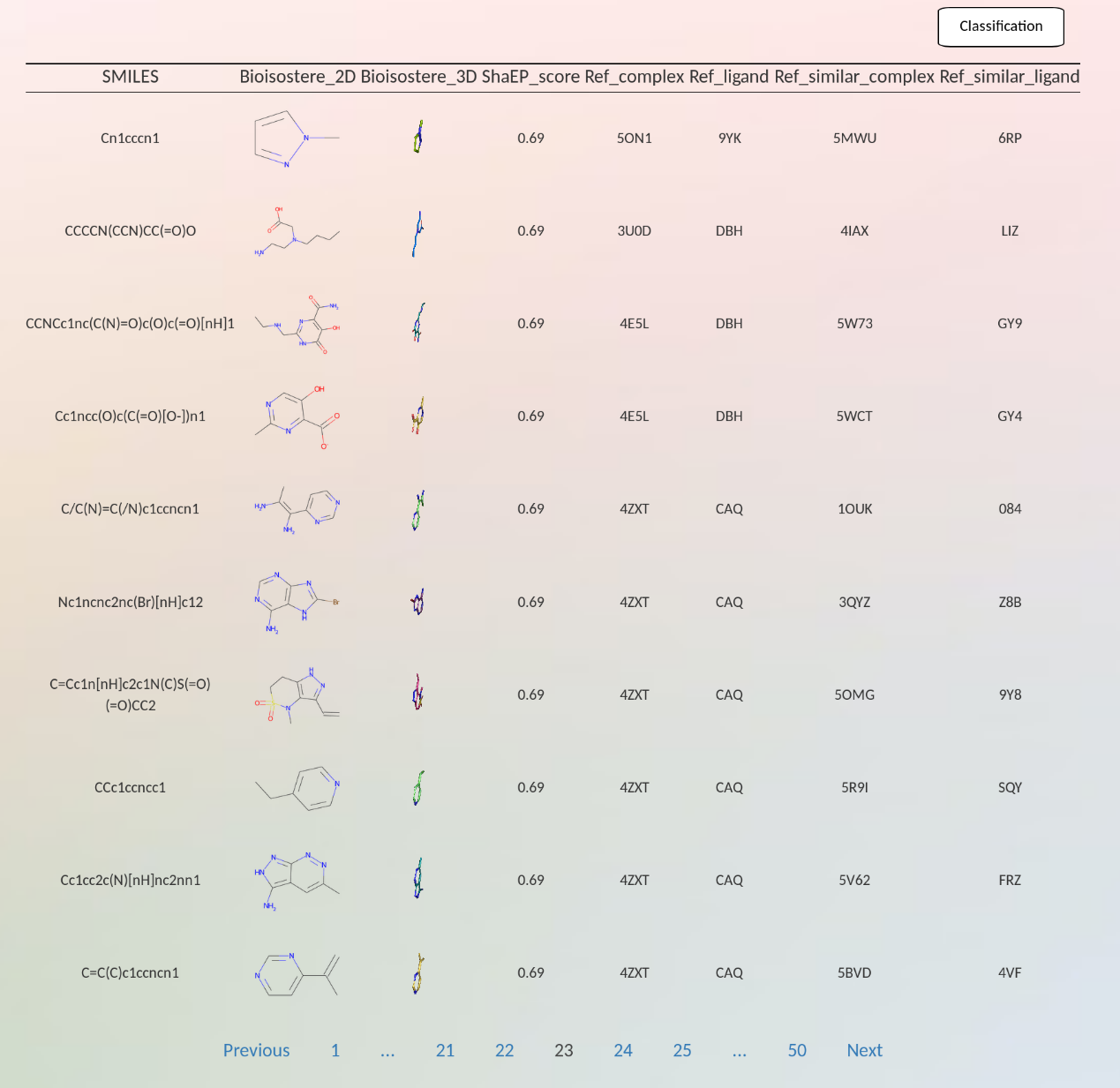

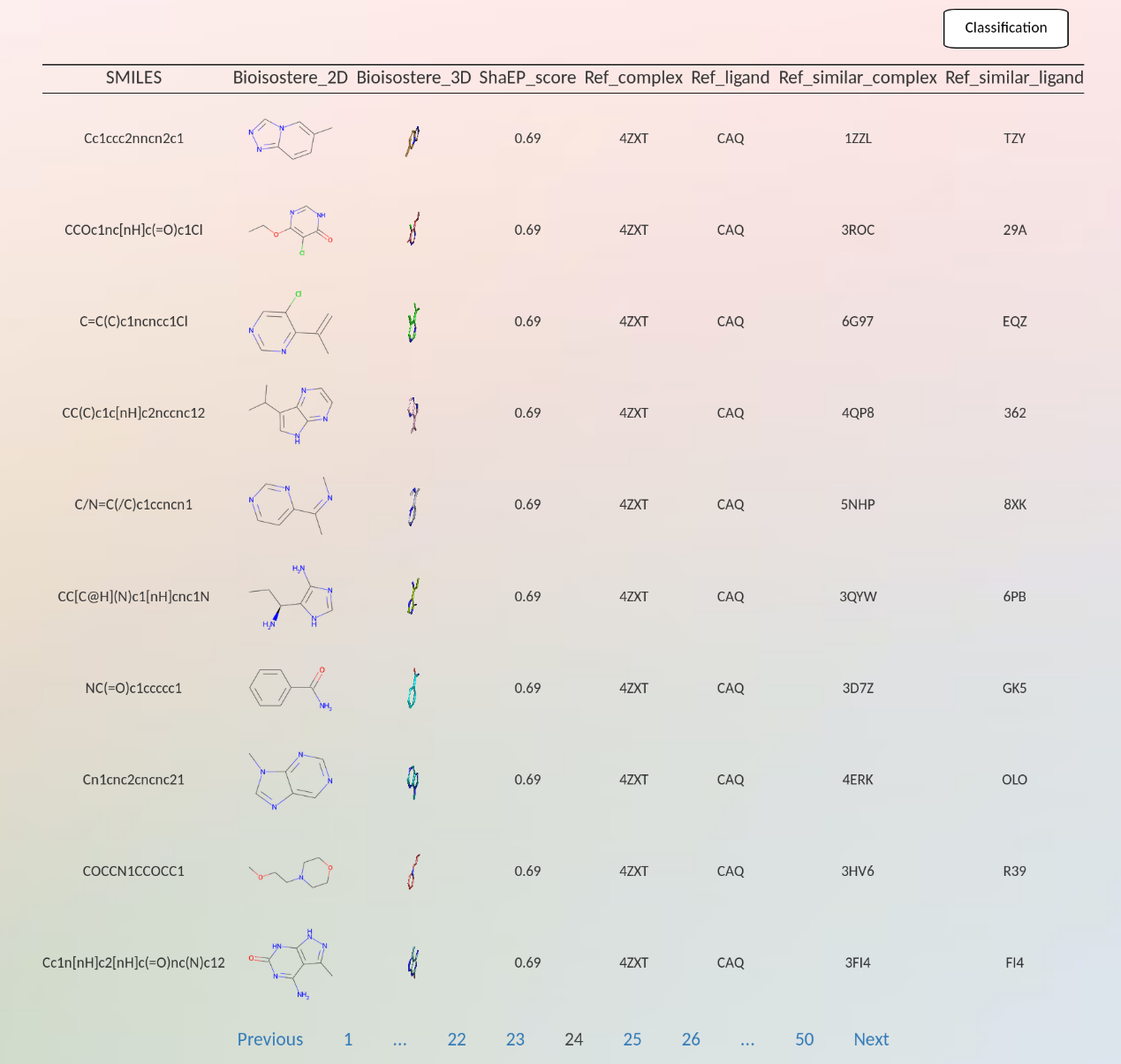

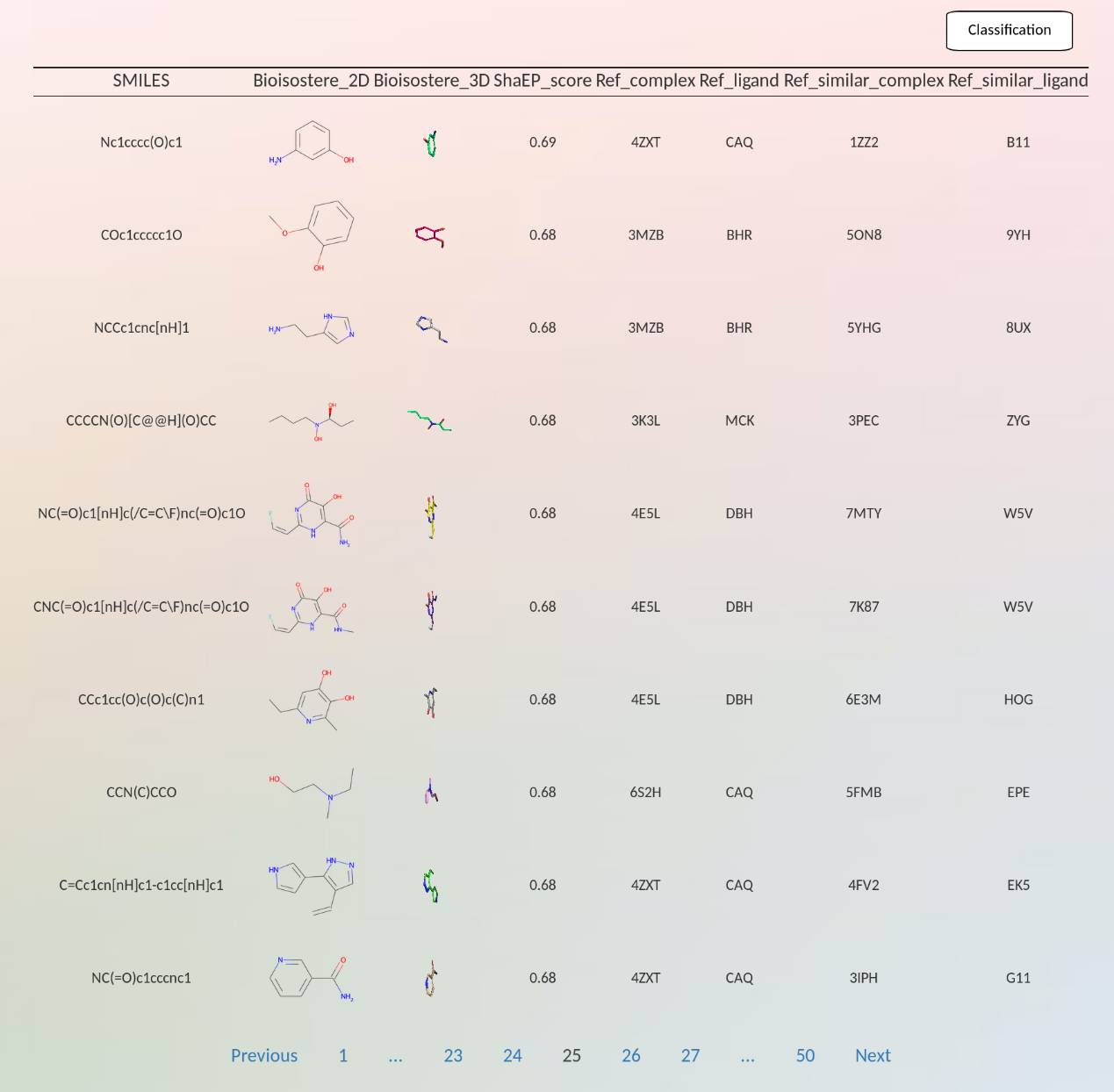

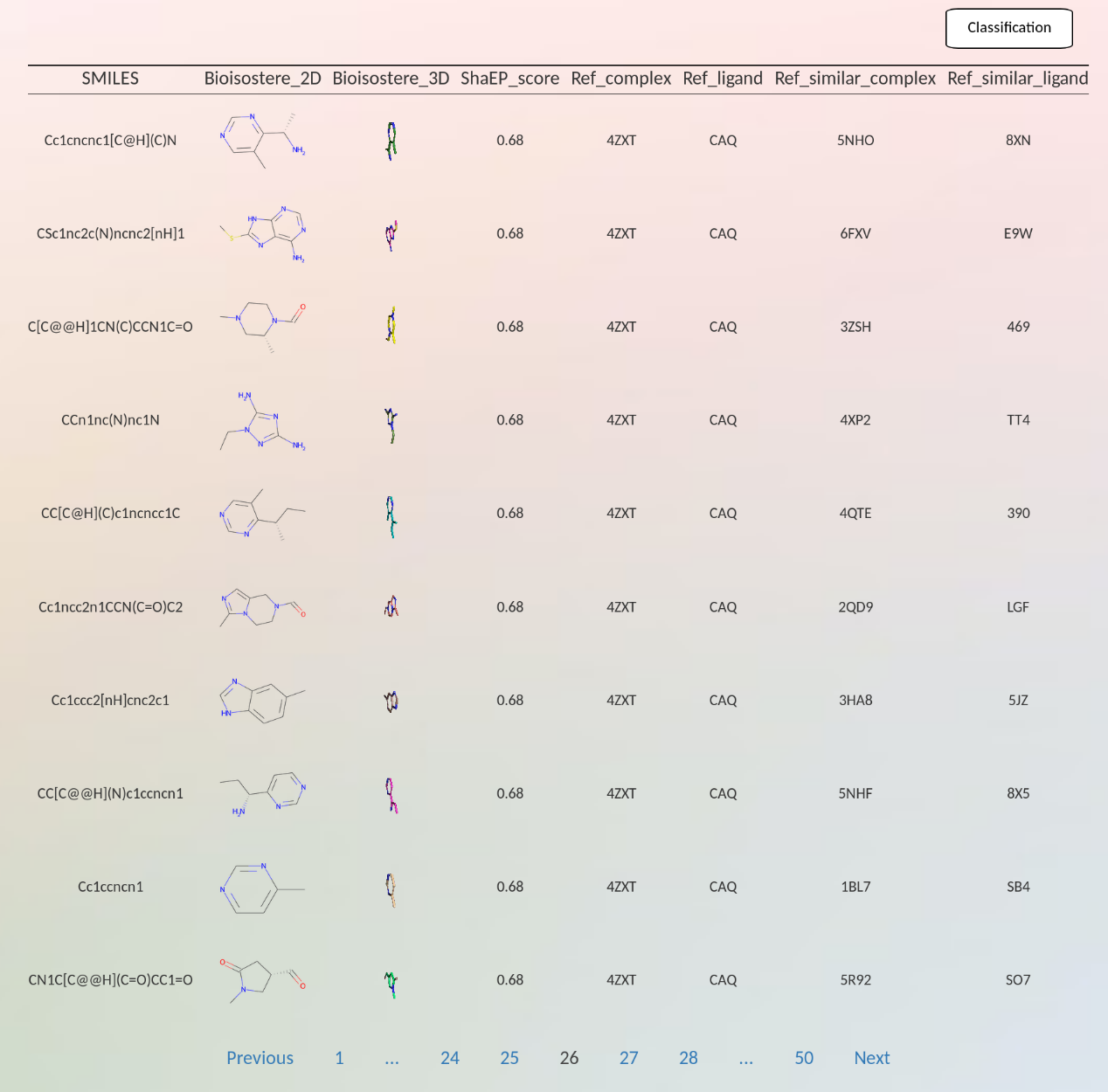

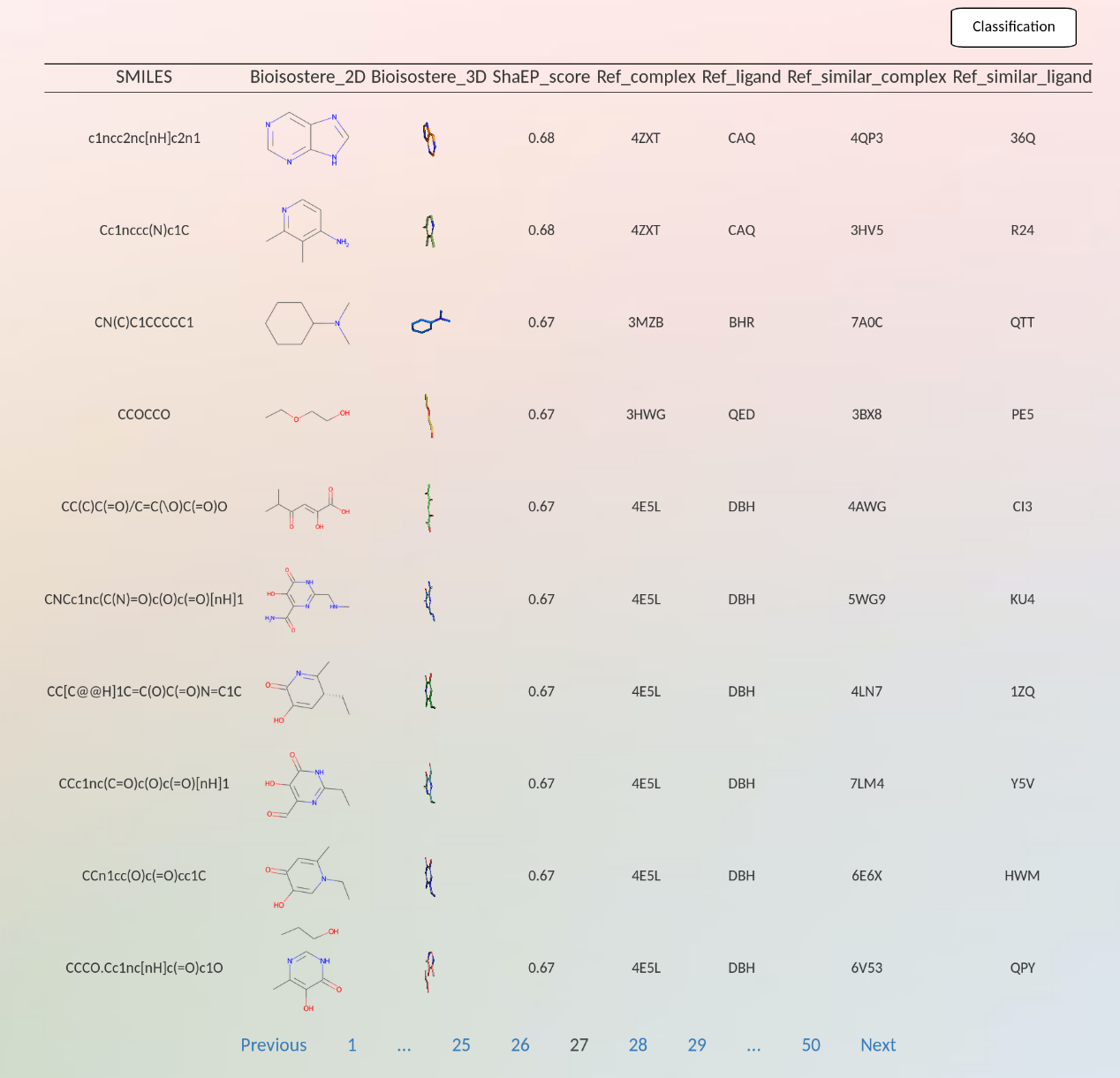

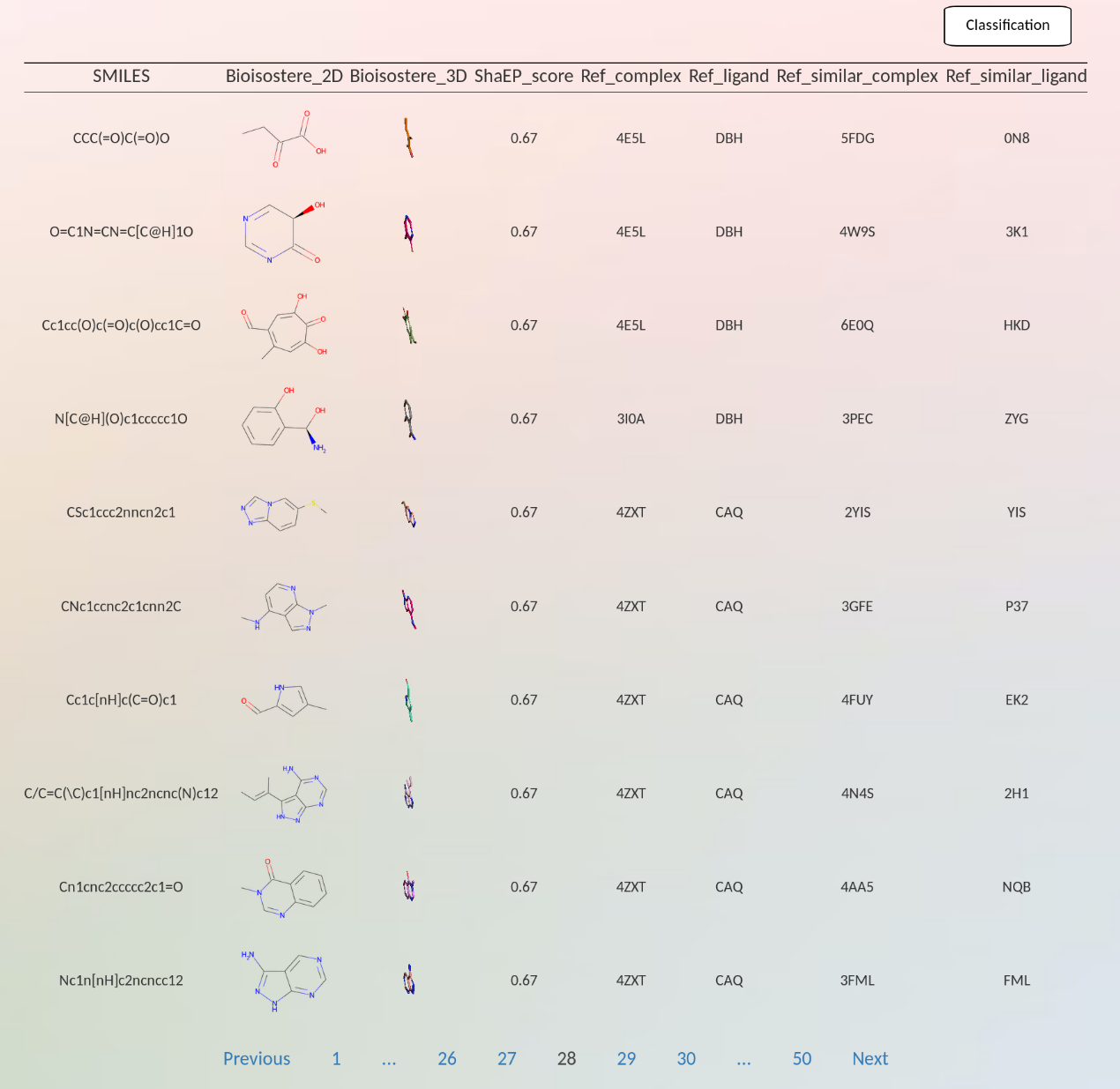

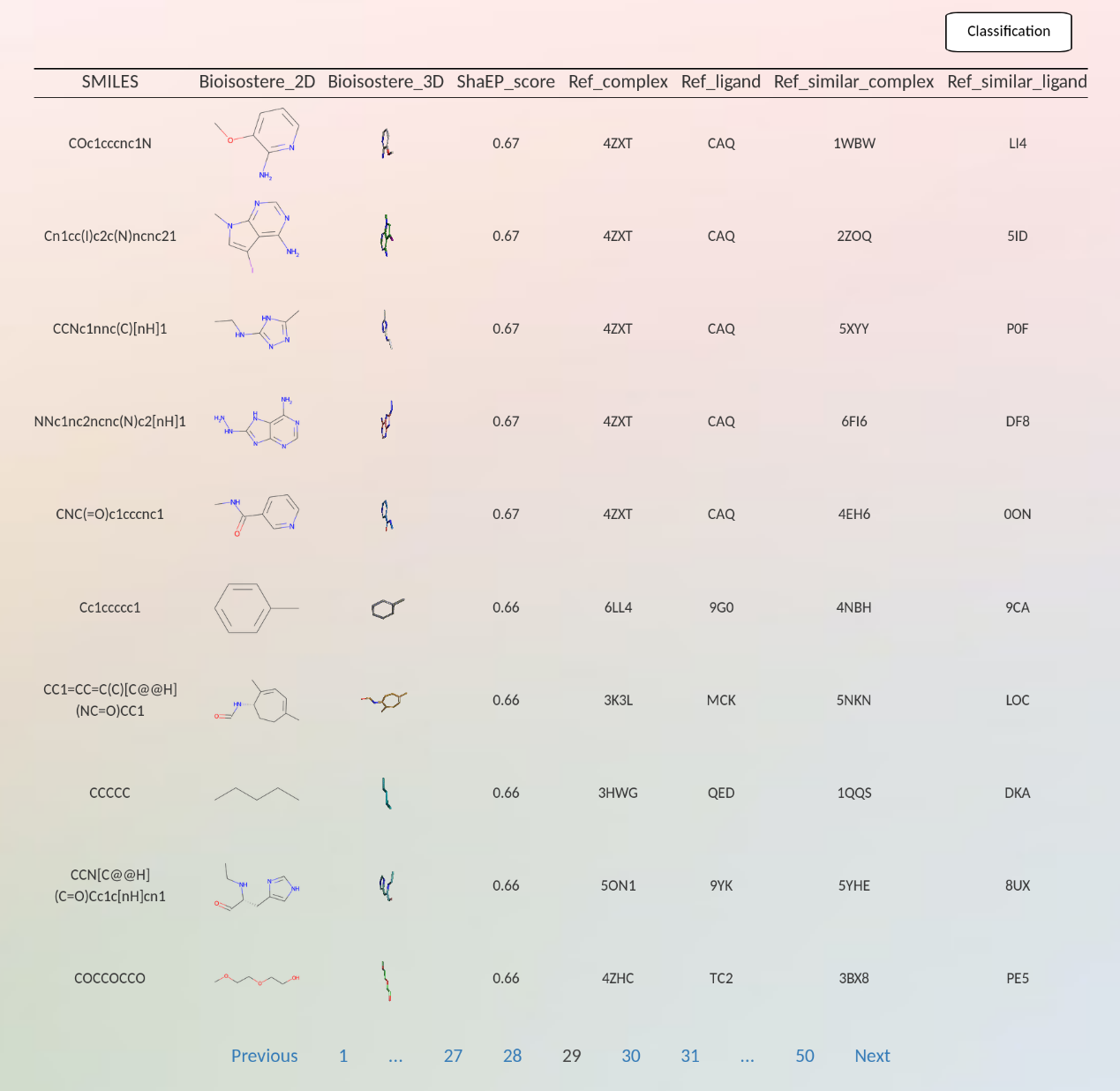

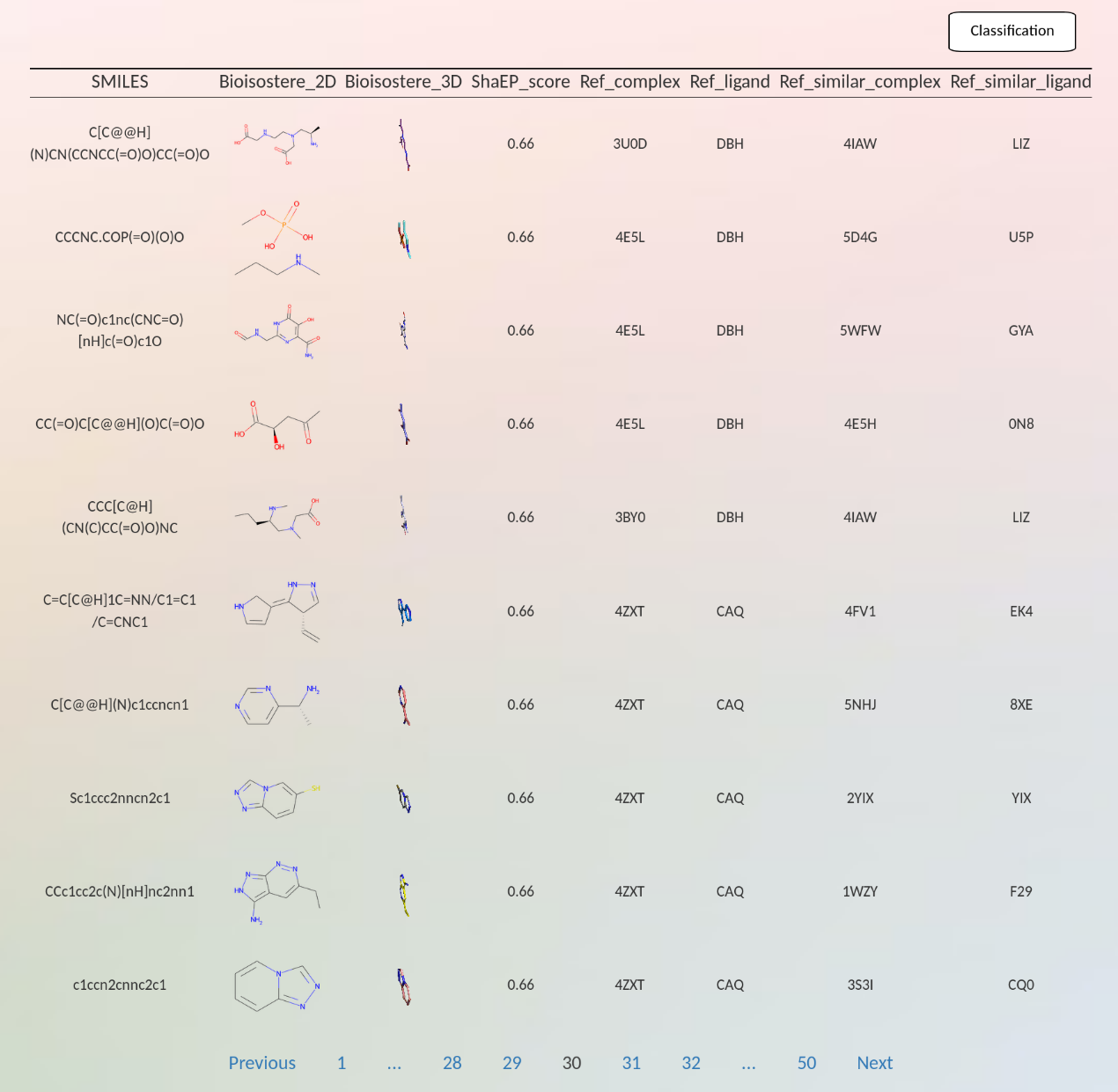

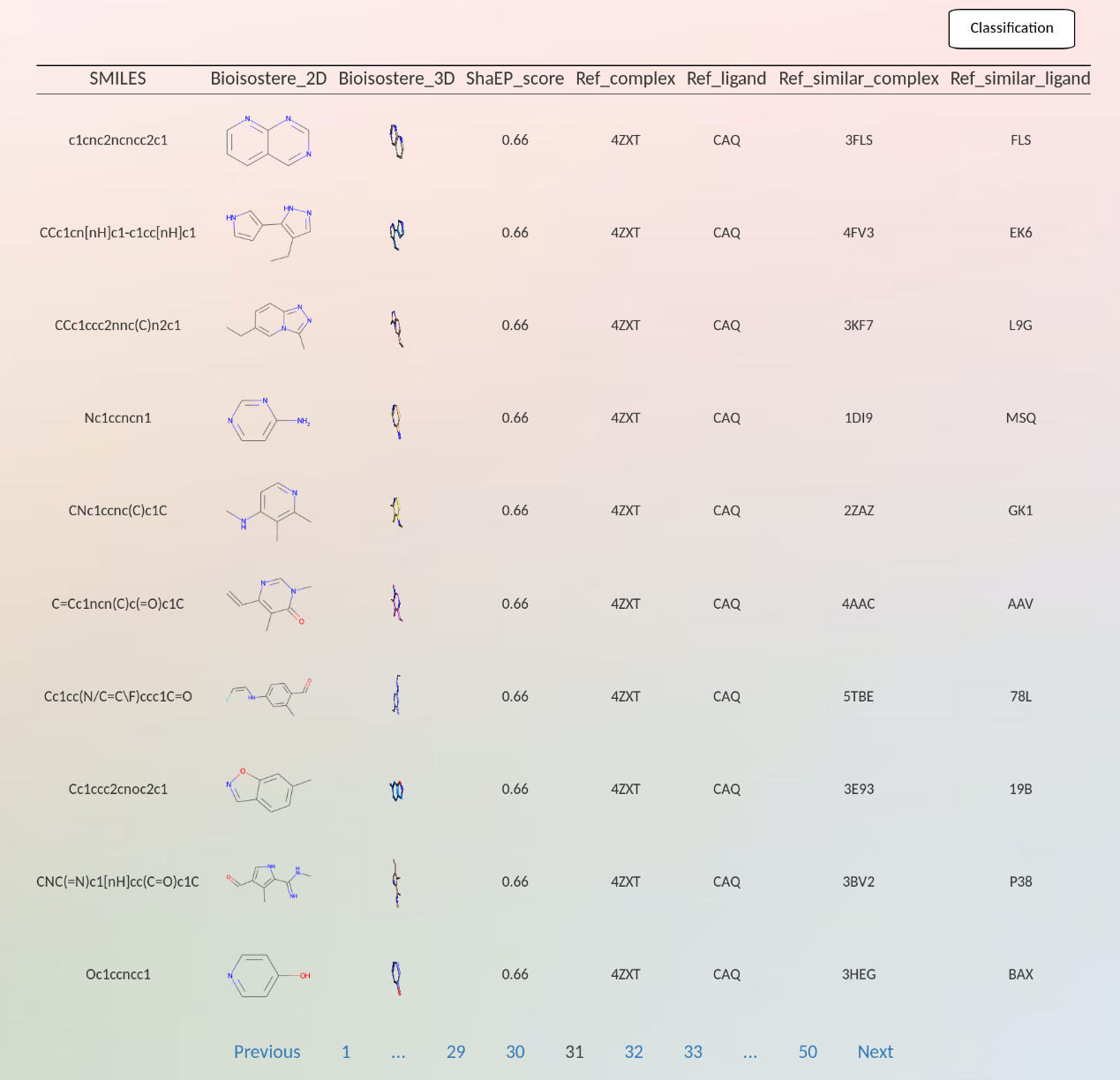

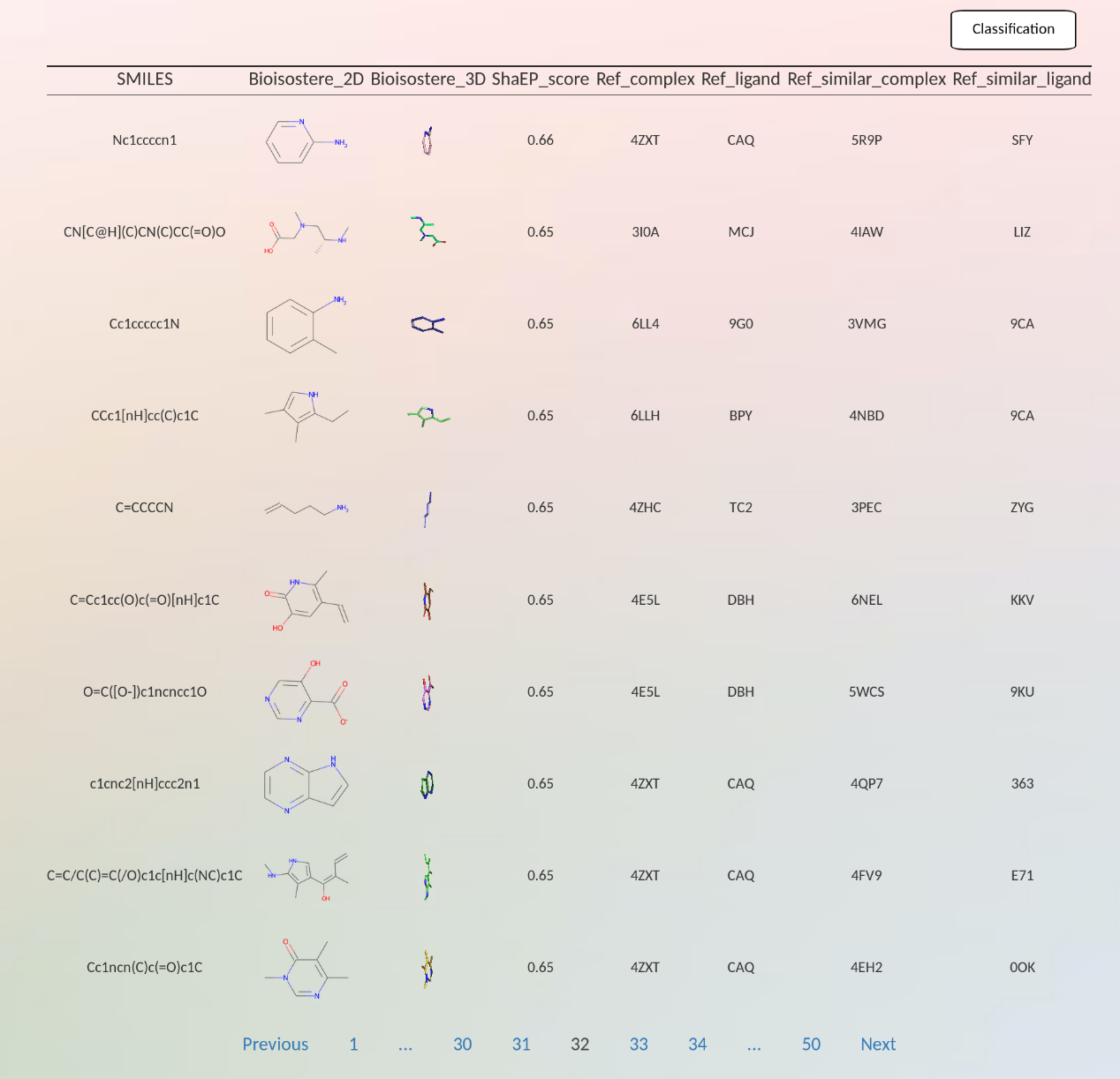

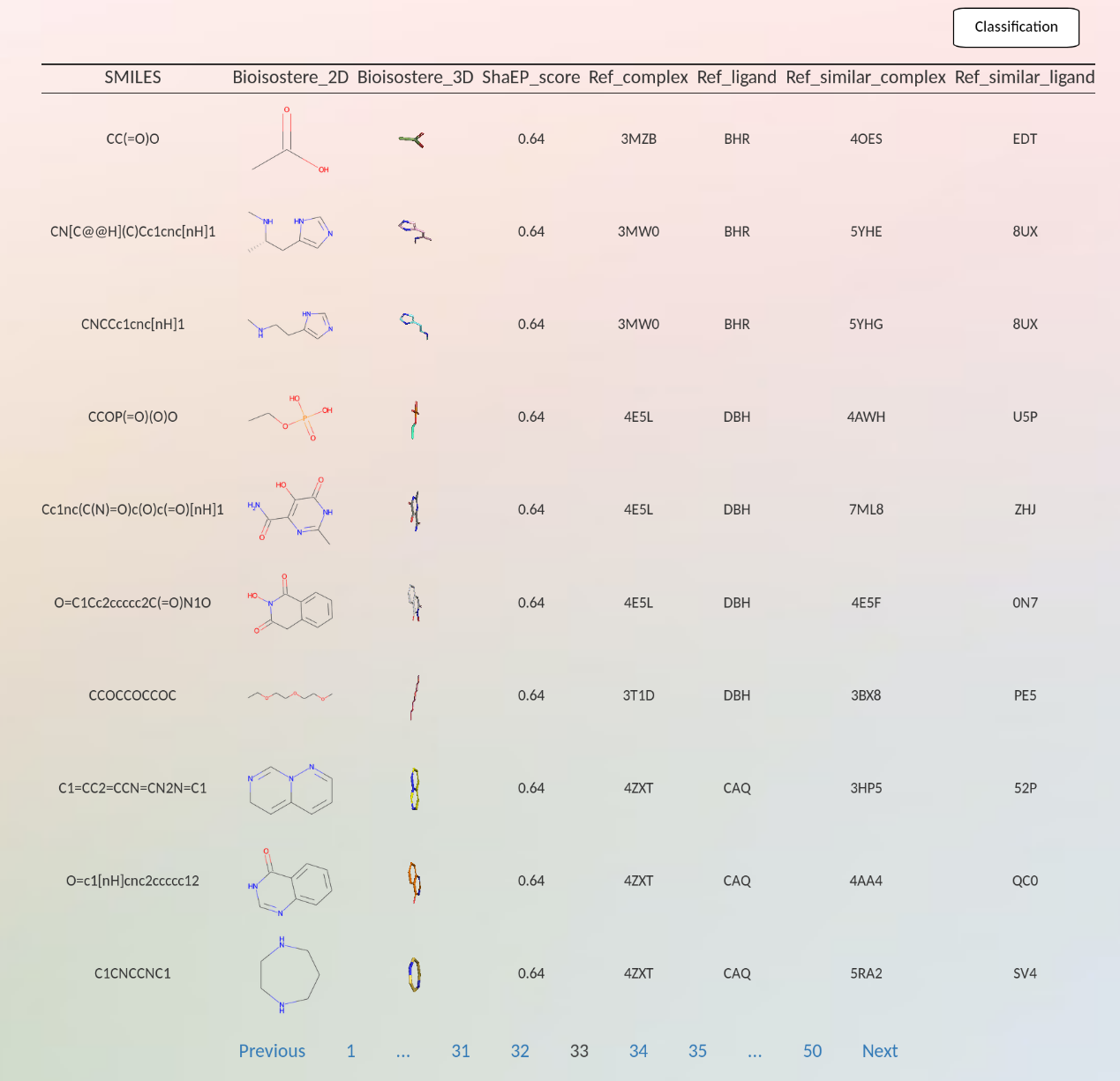

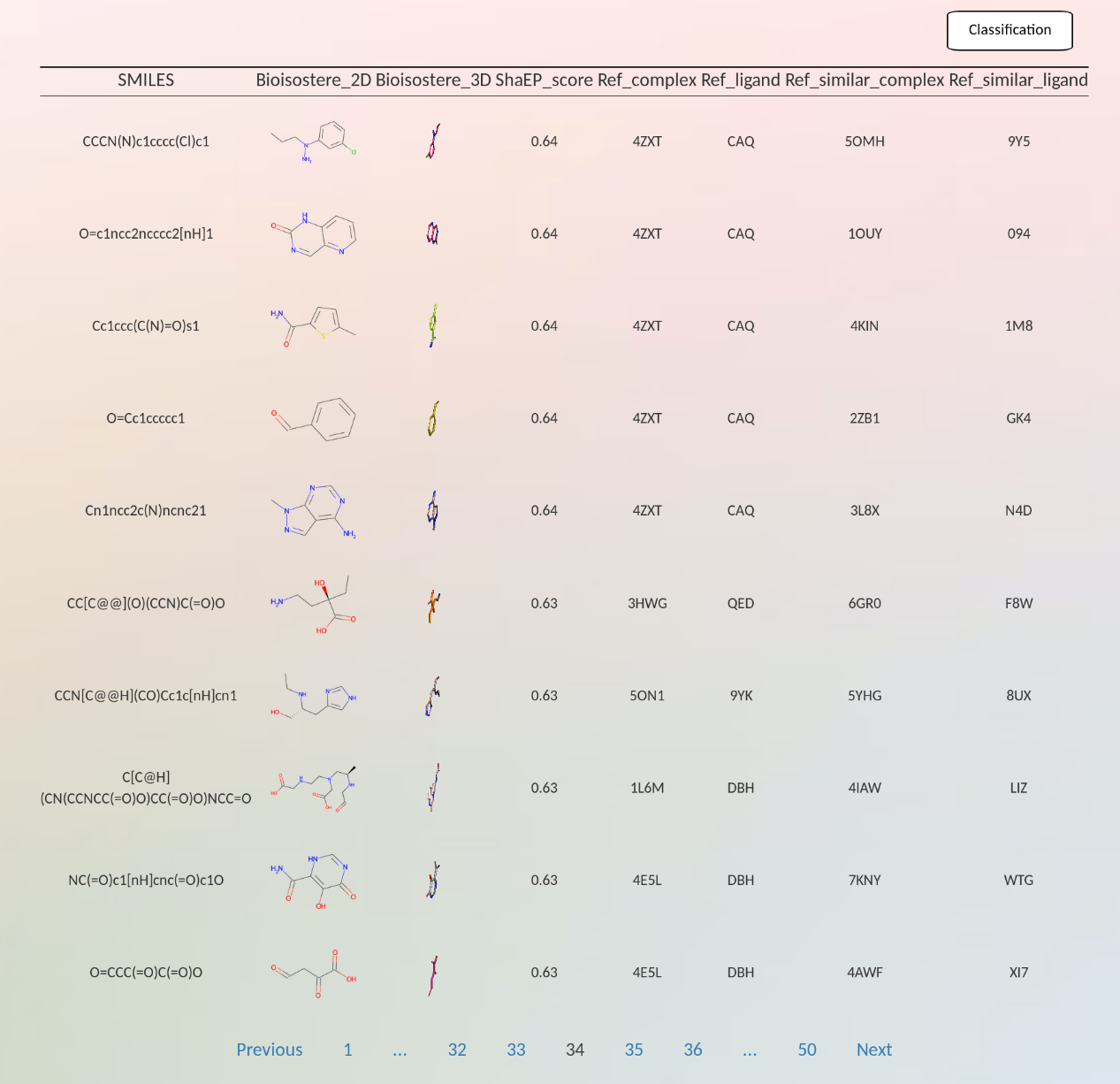

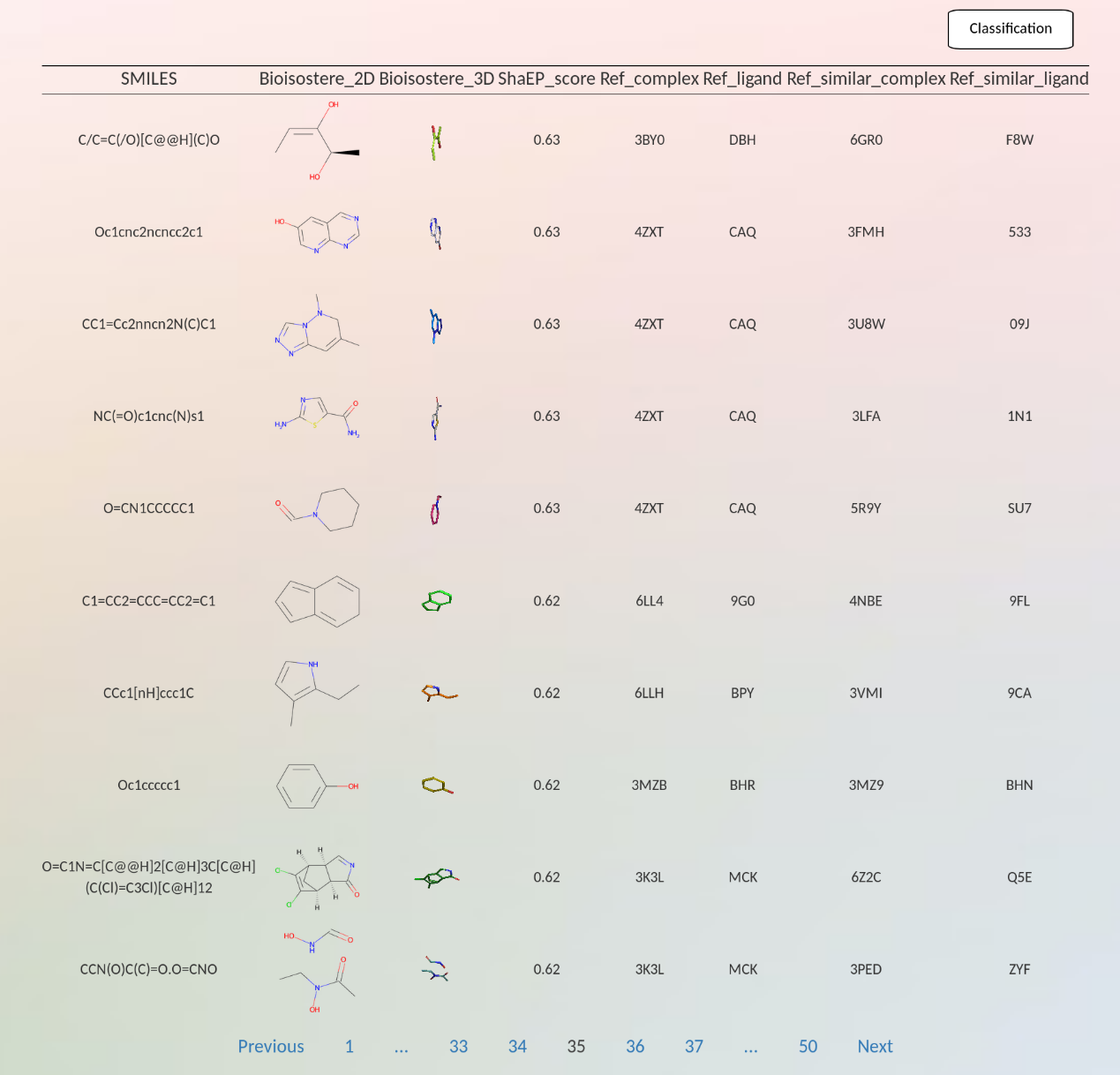

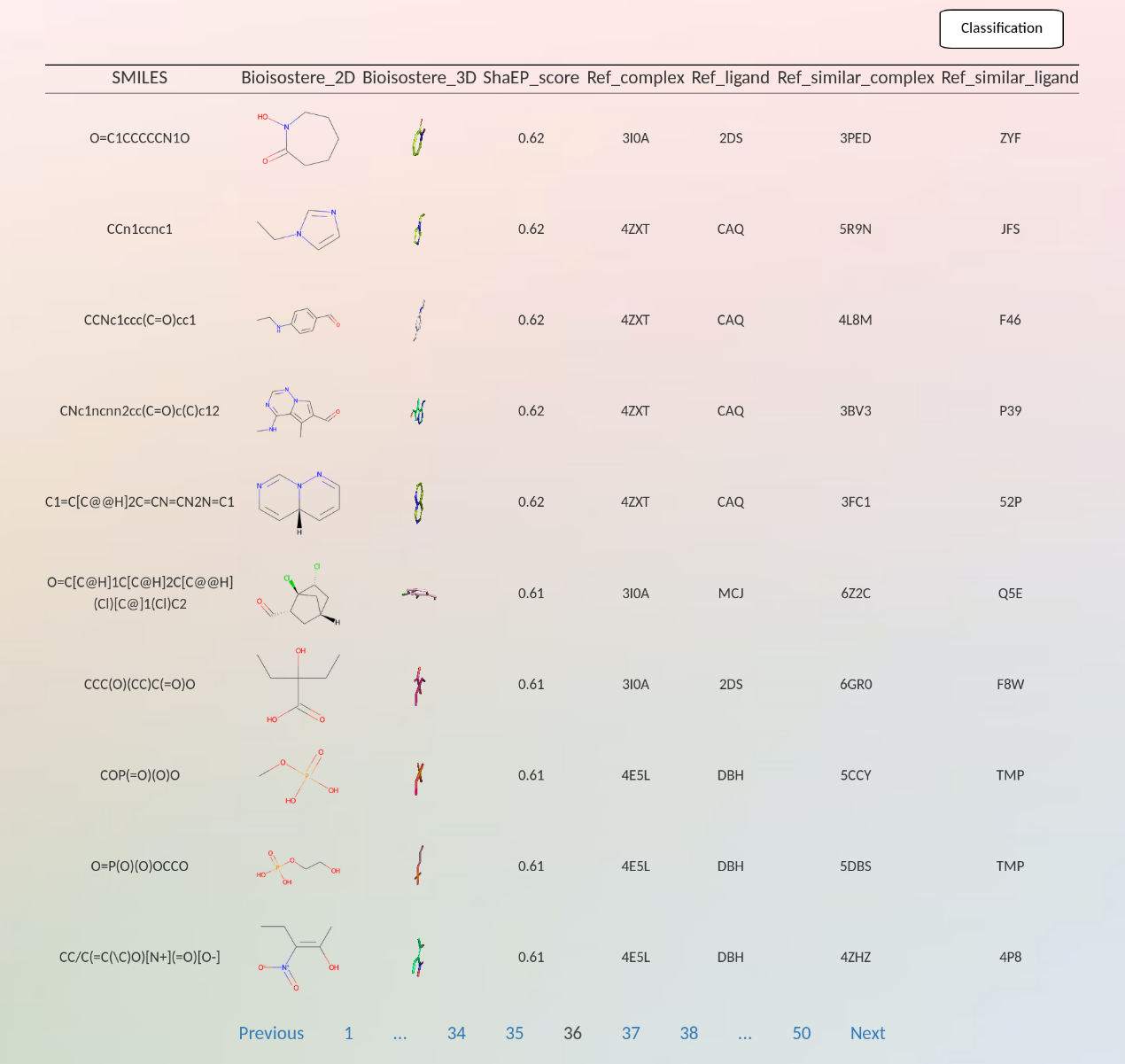

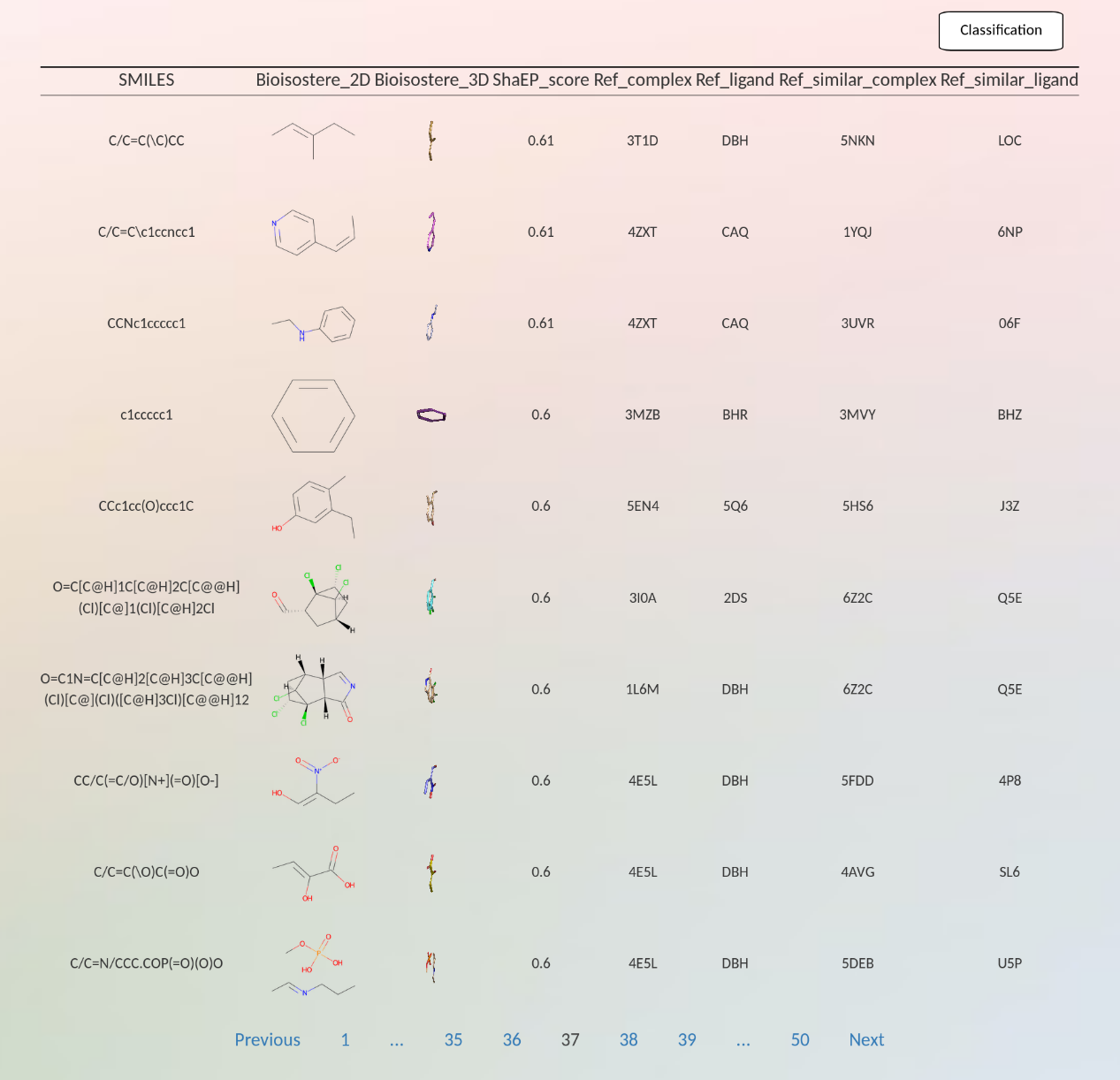

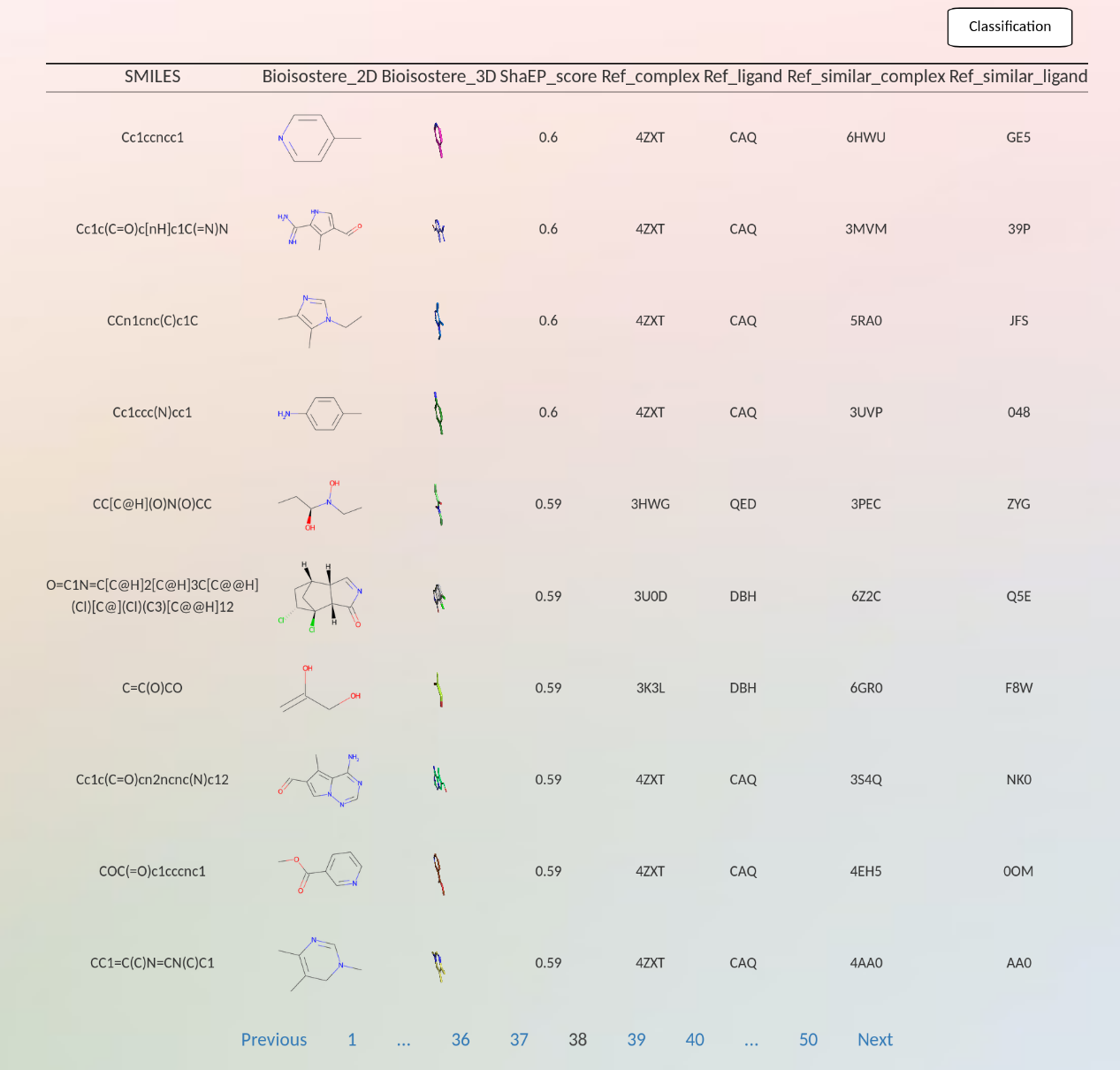

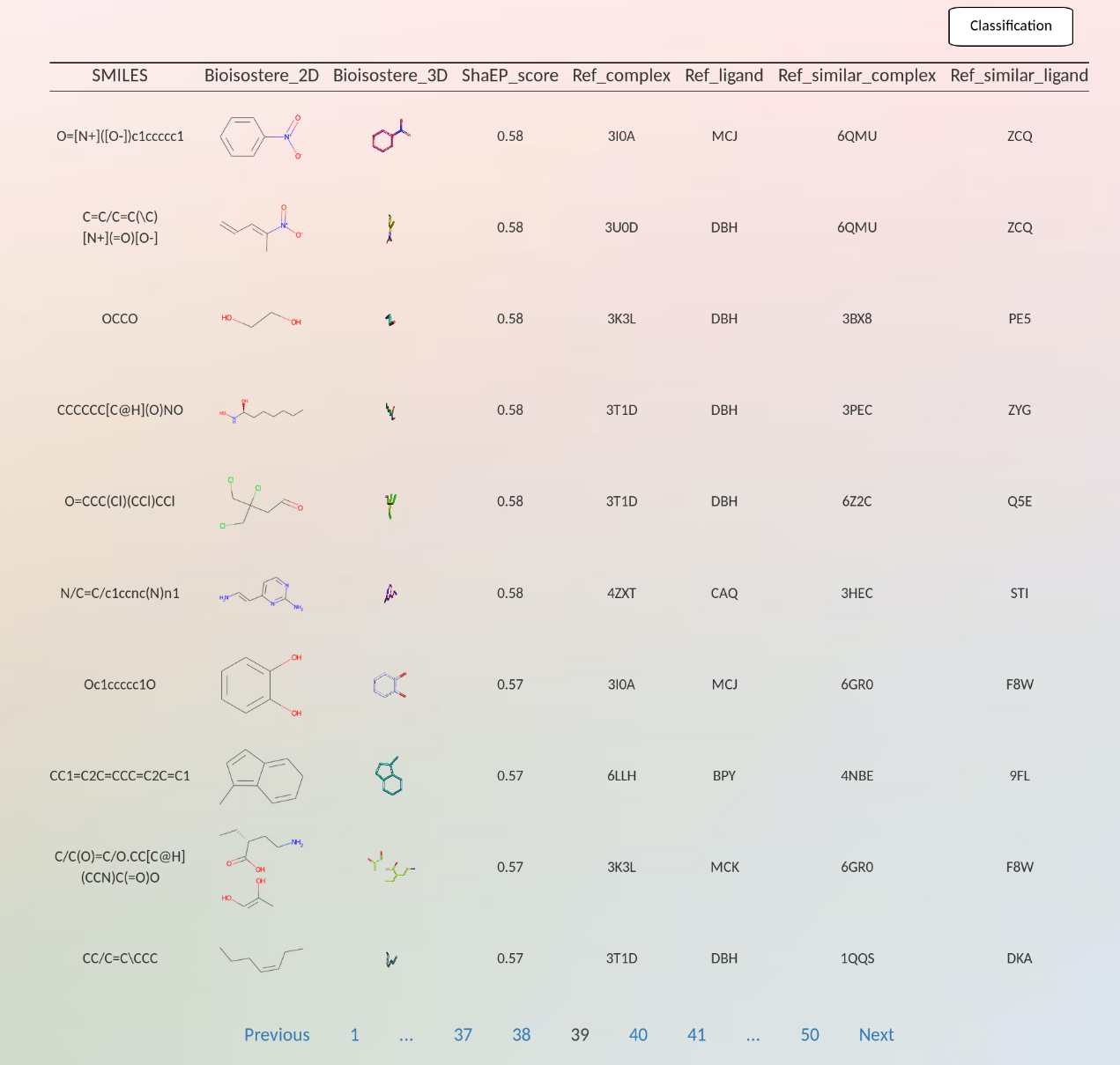

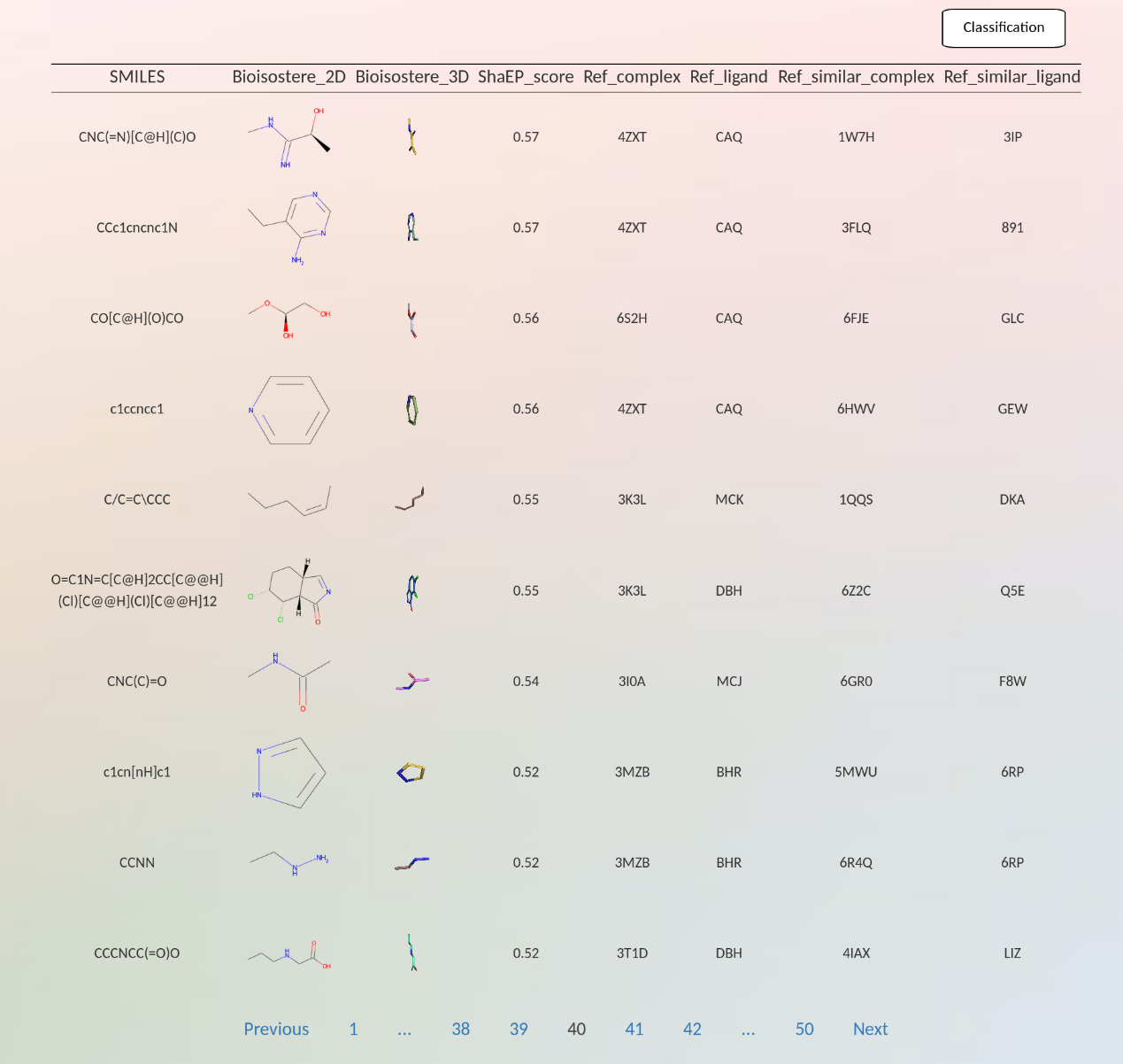

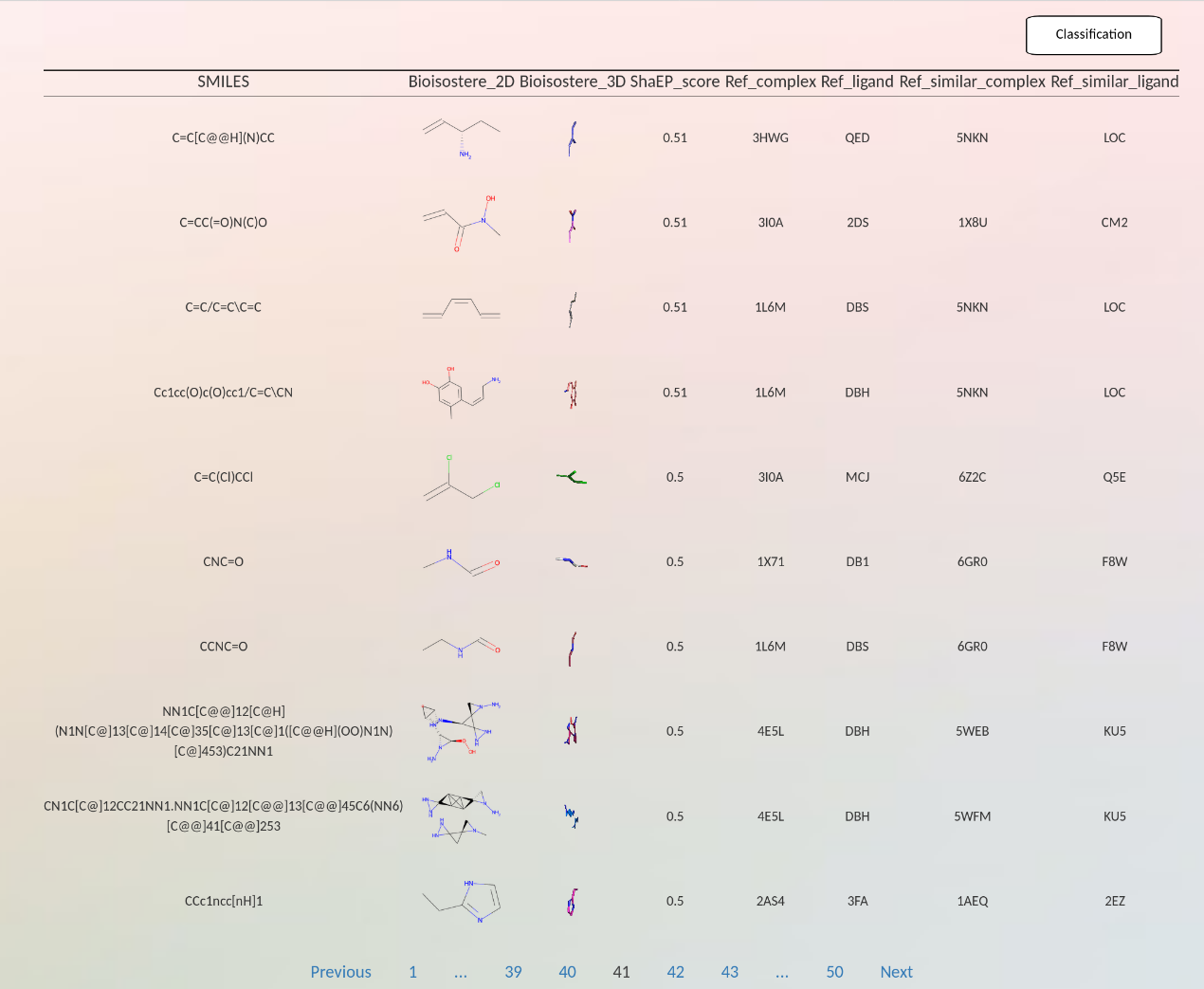

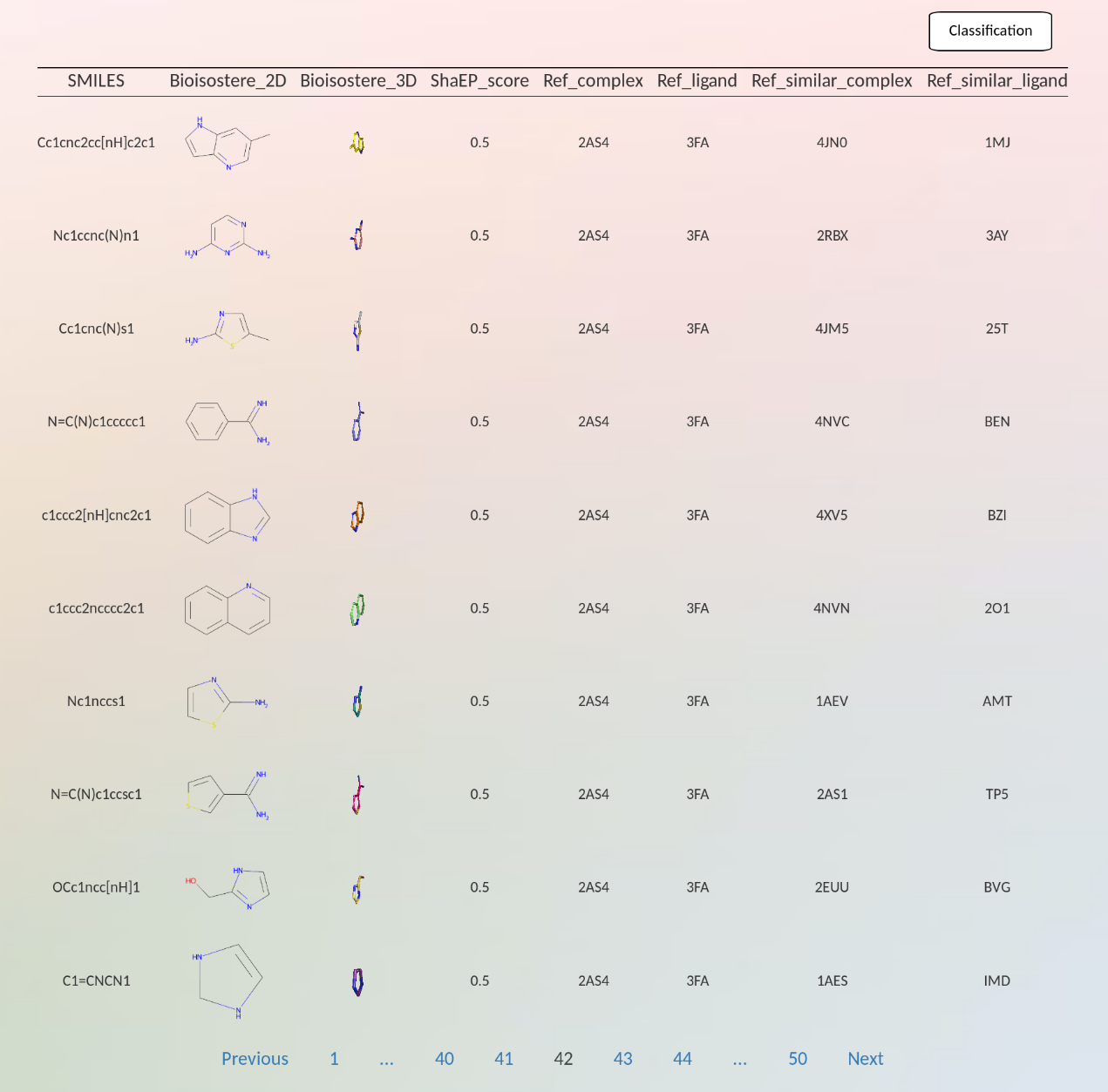

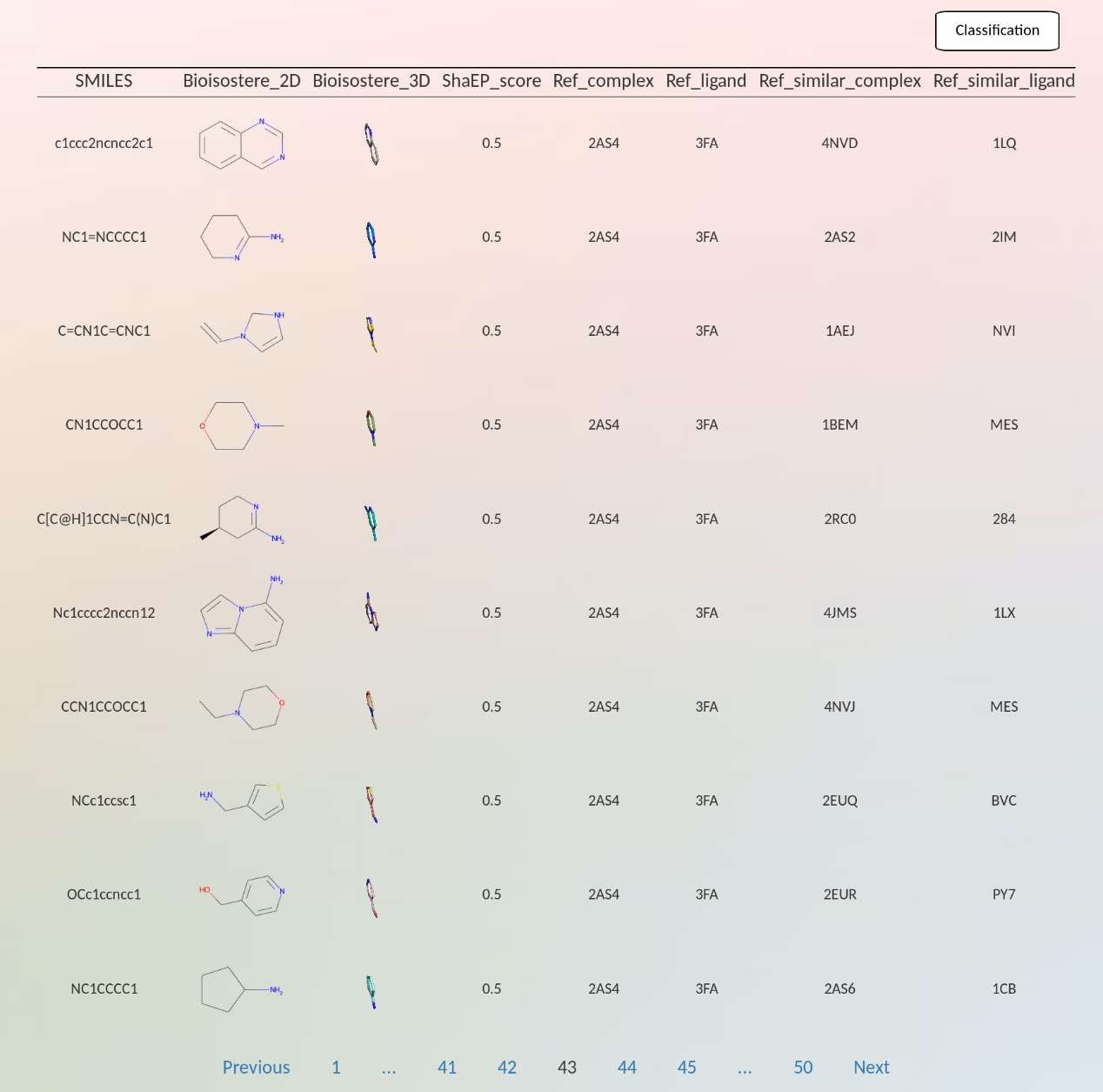

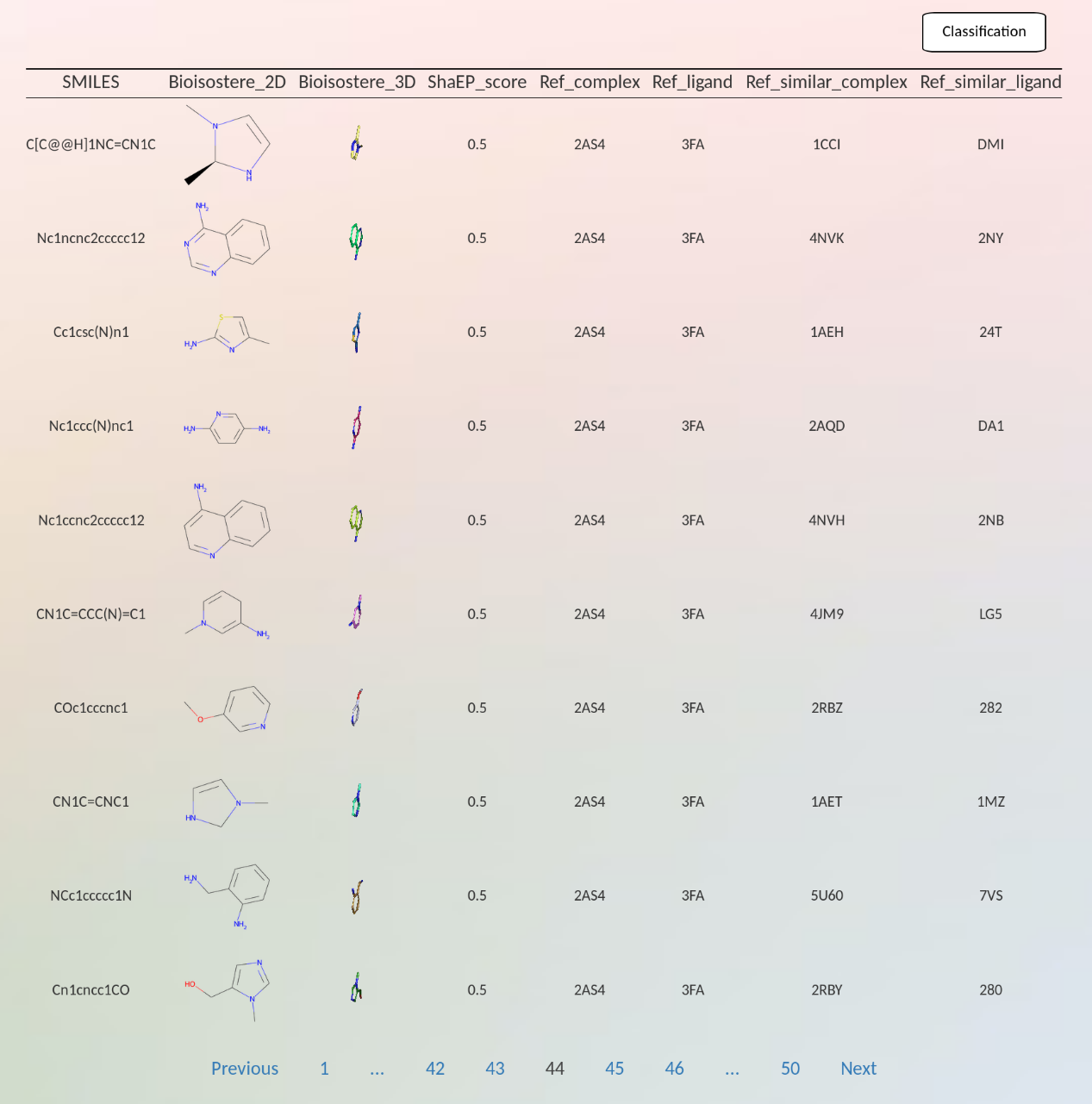

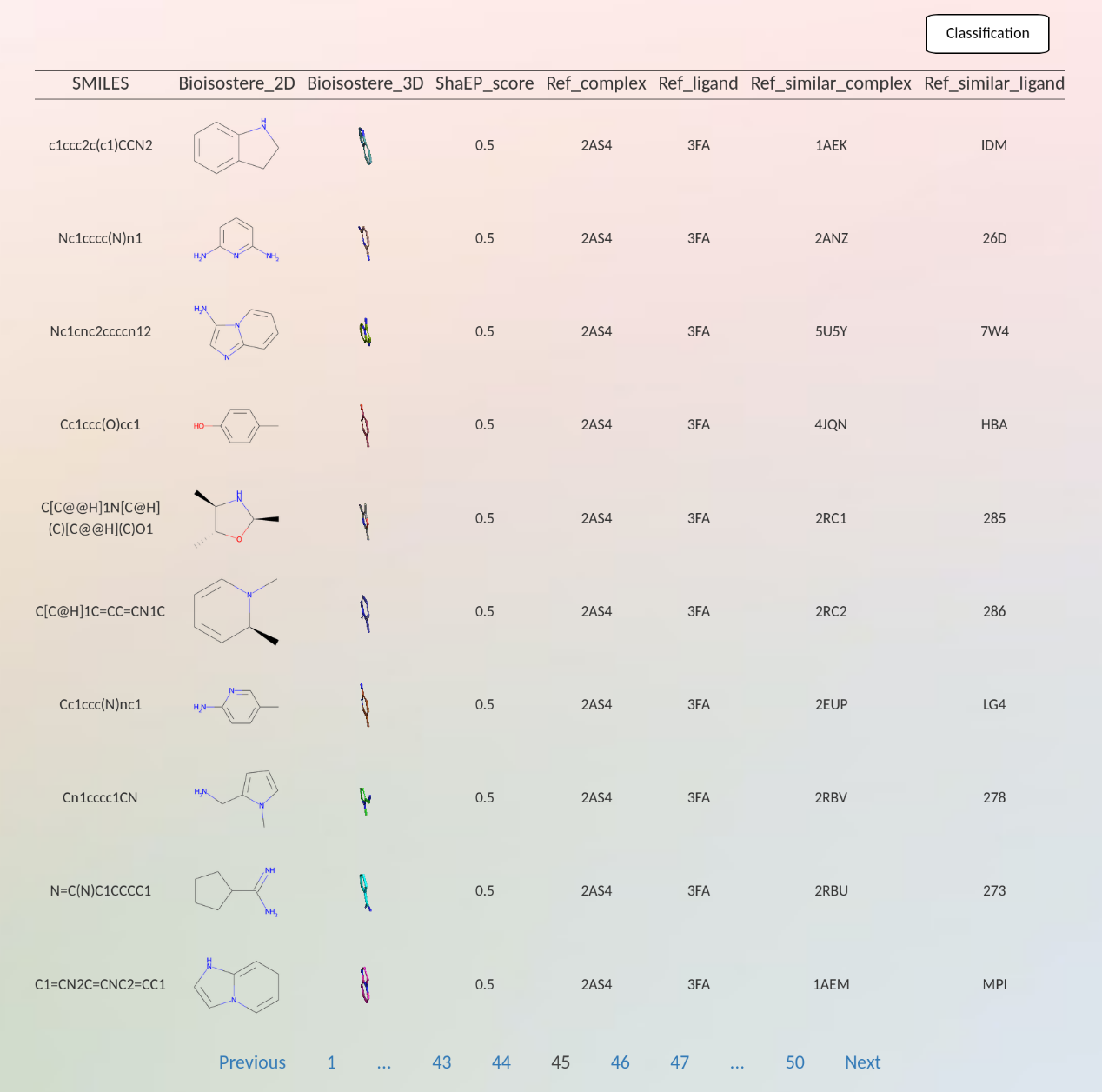

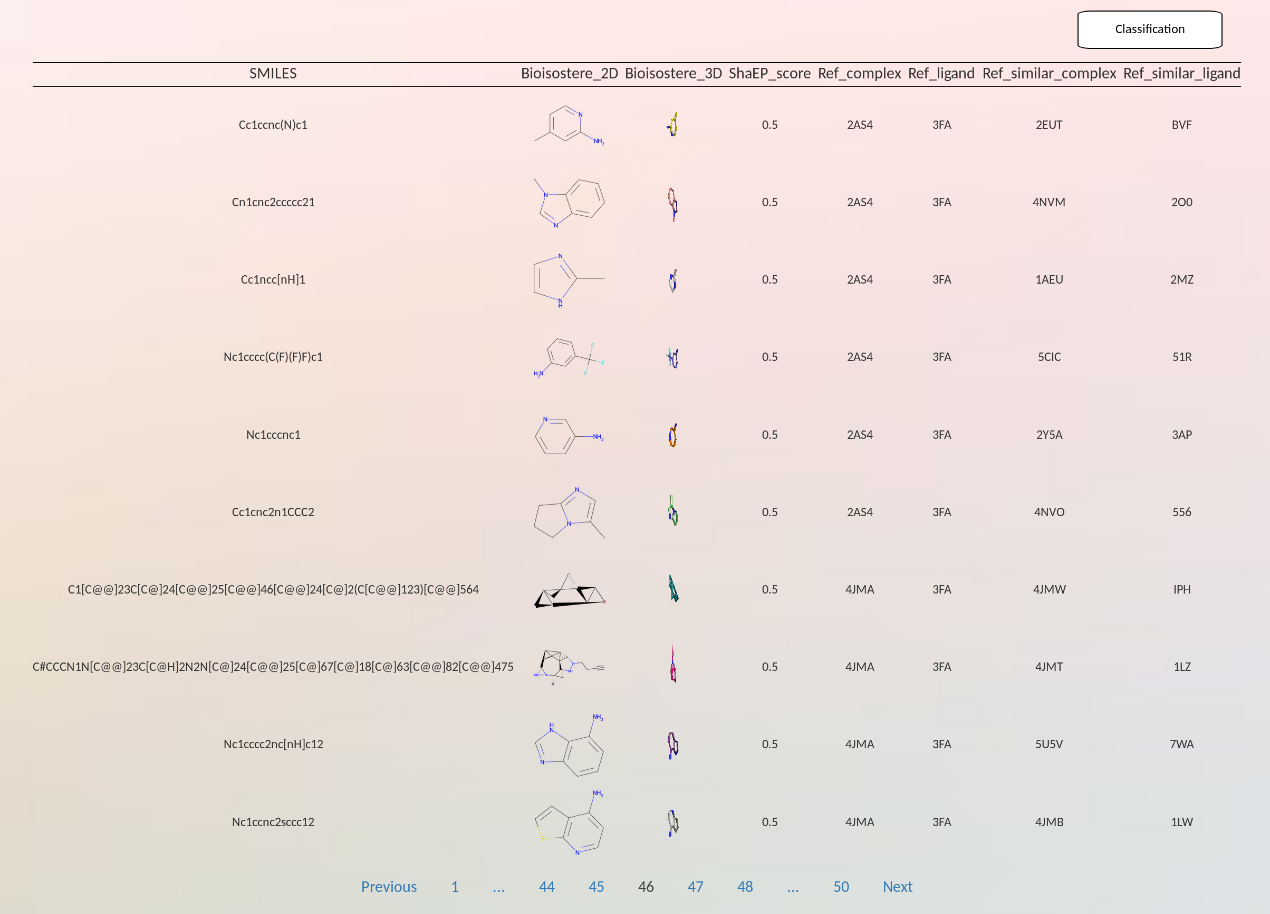


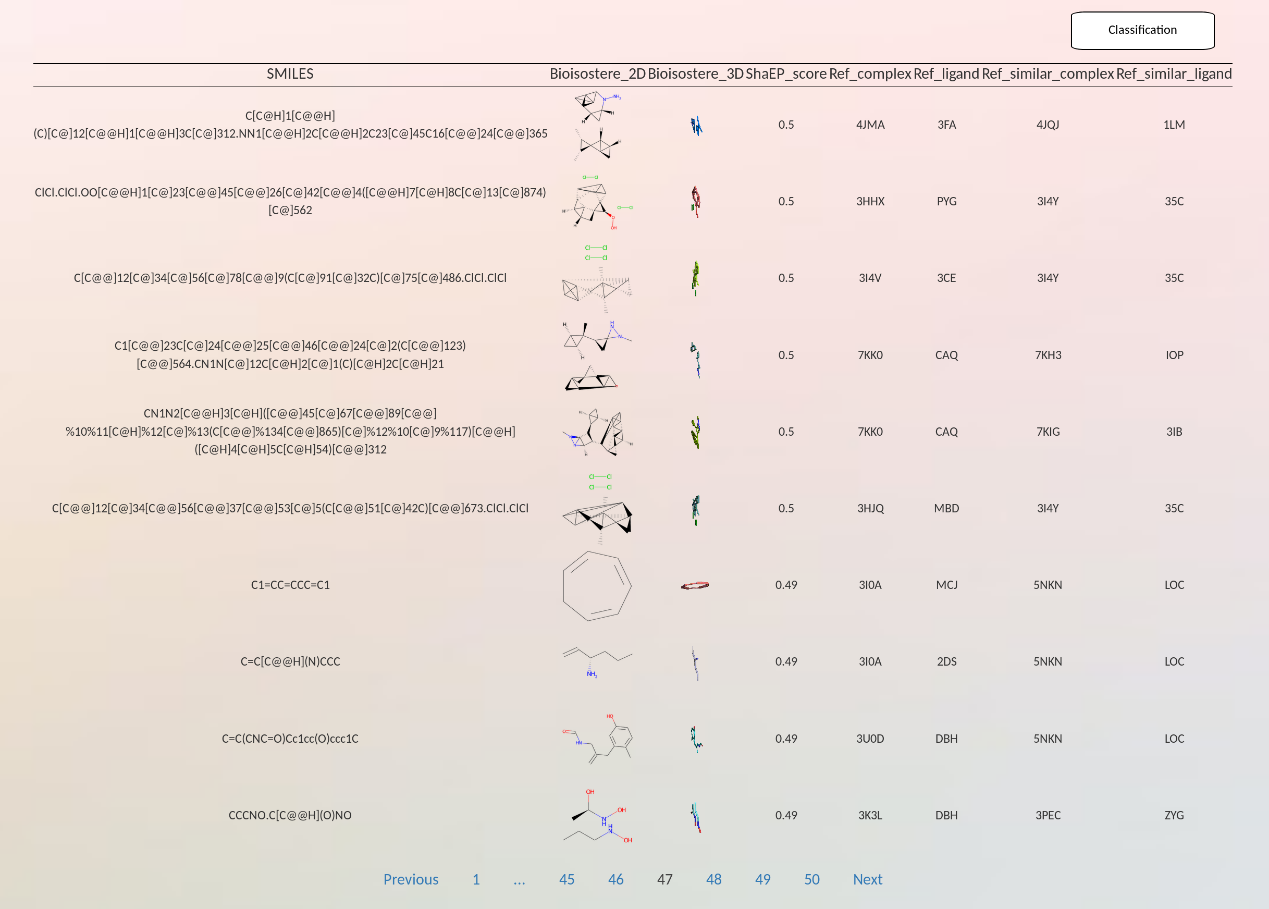


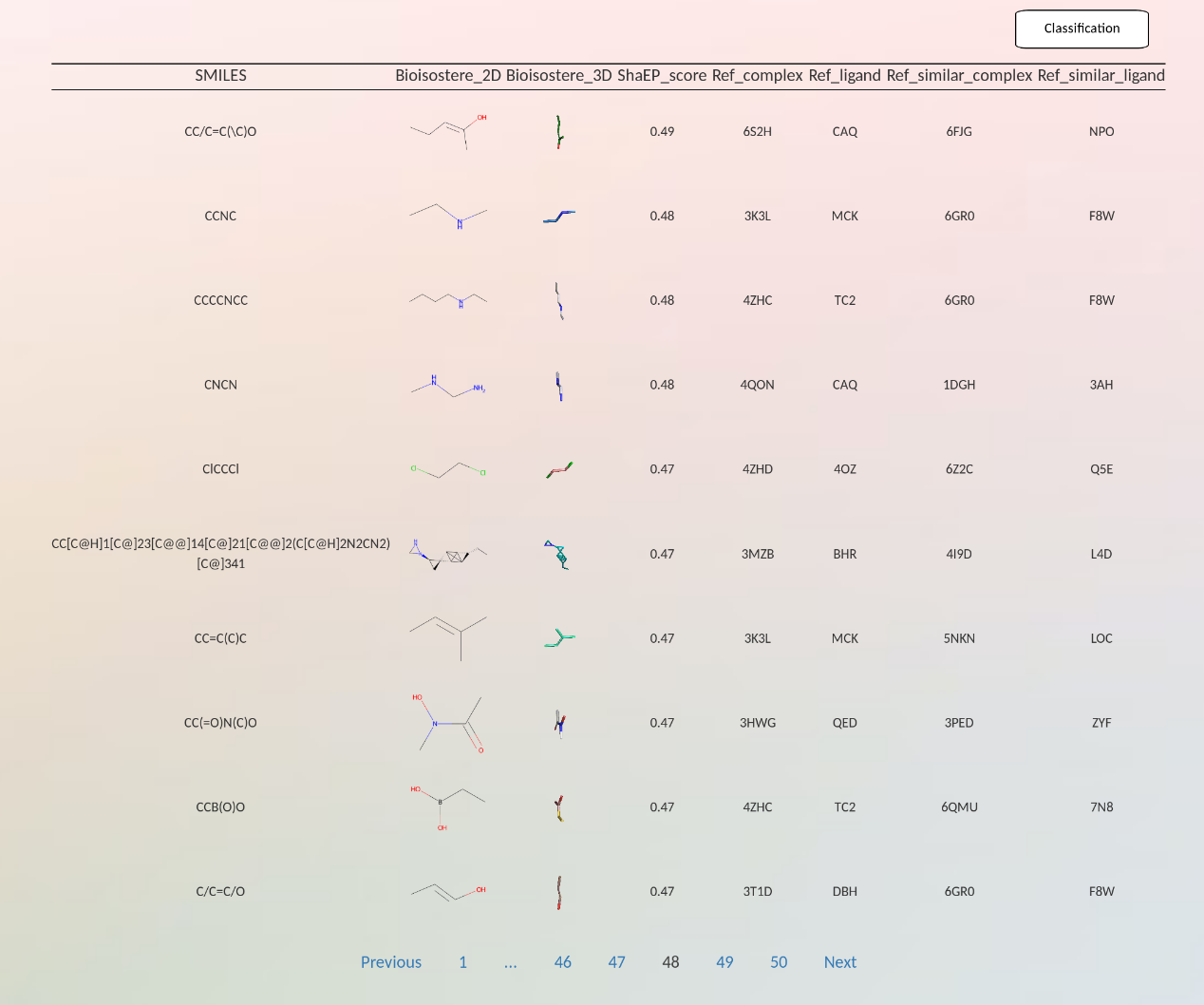

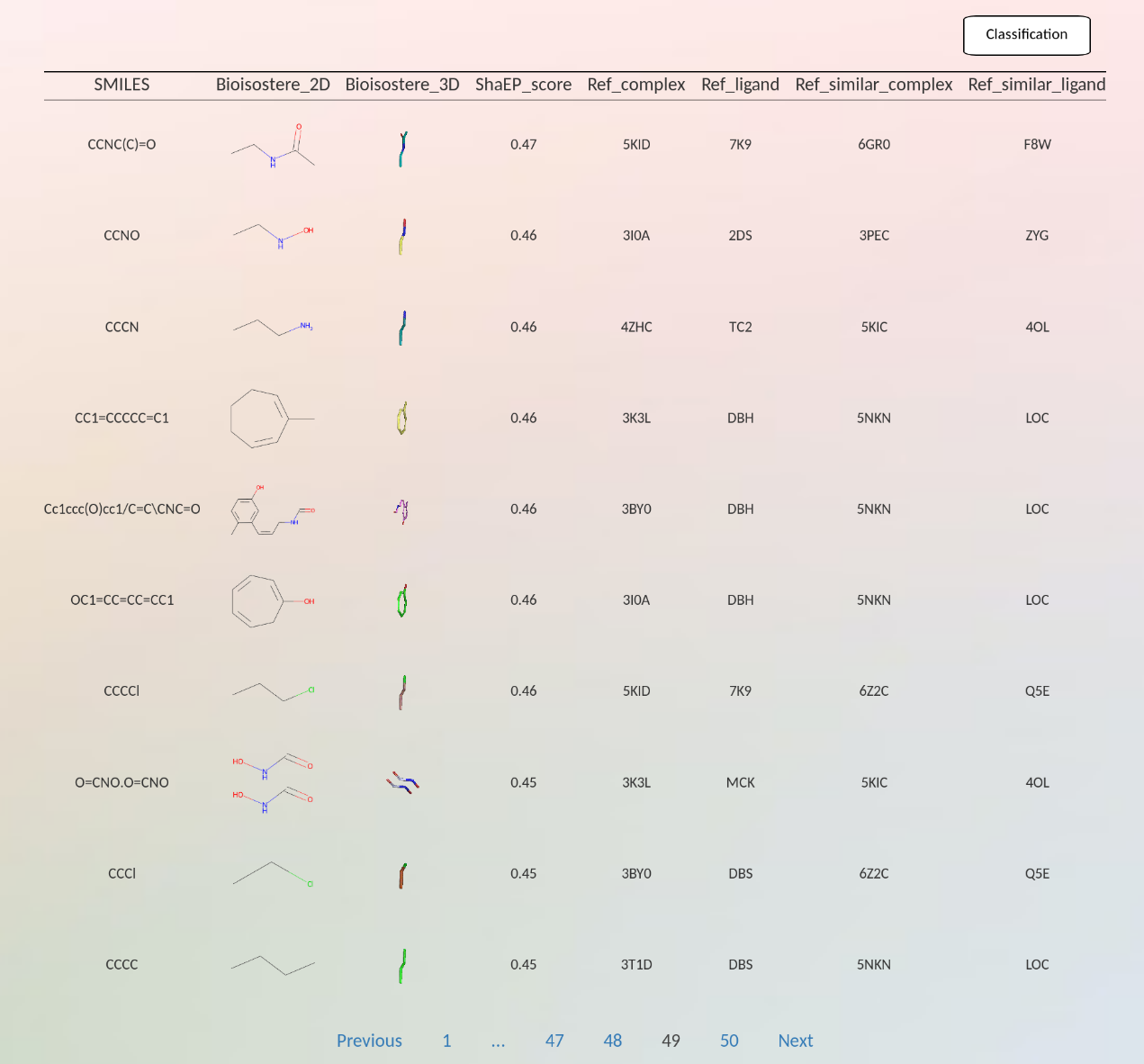


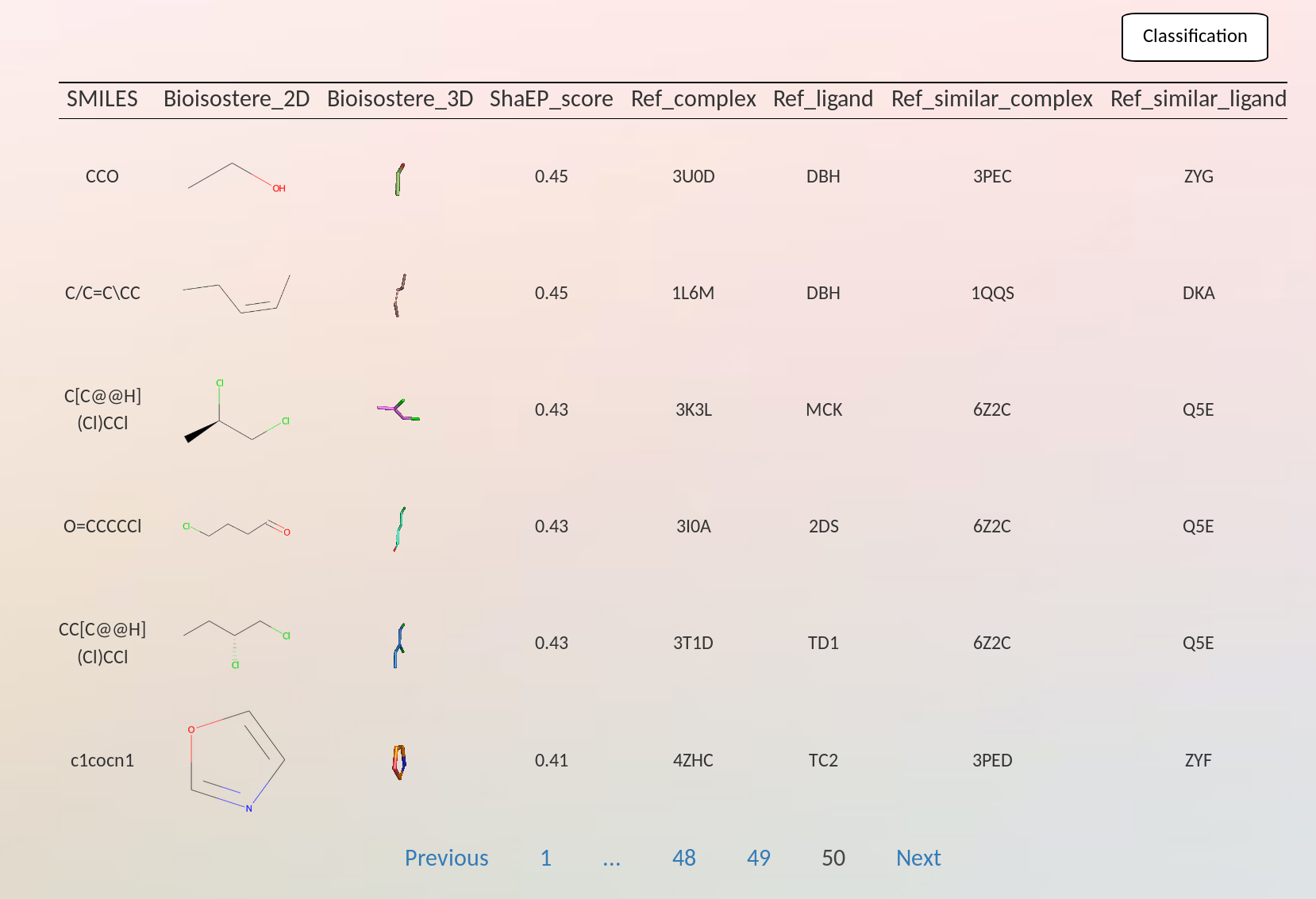


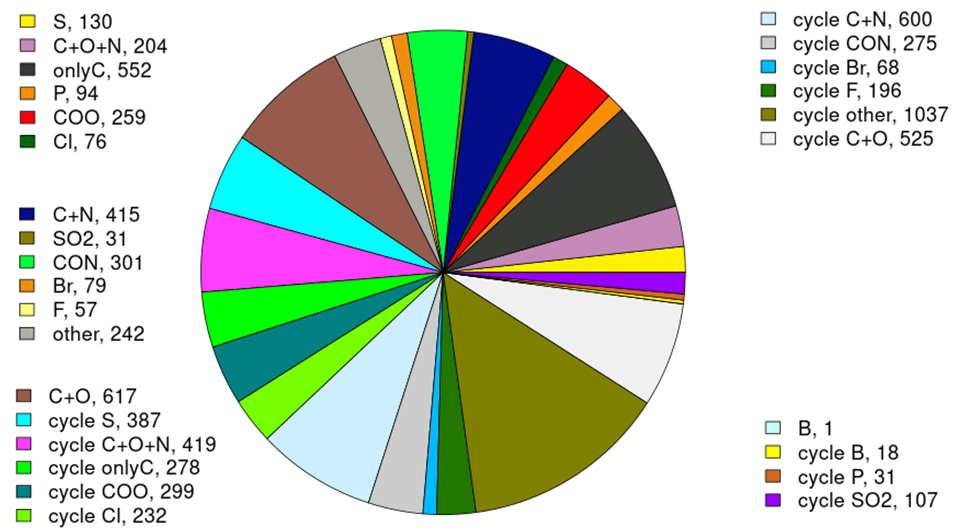


S3. The LSR subgroup of 4-substituent catechol categorized as cycle C+O+N

S4.


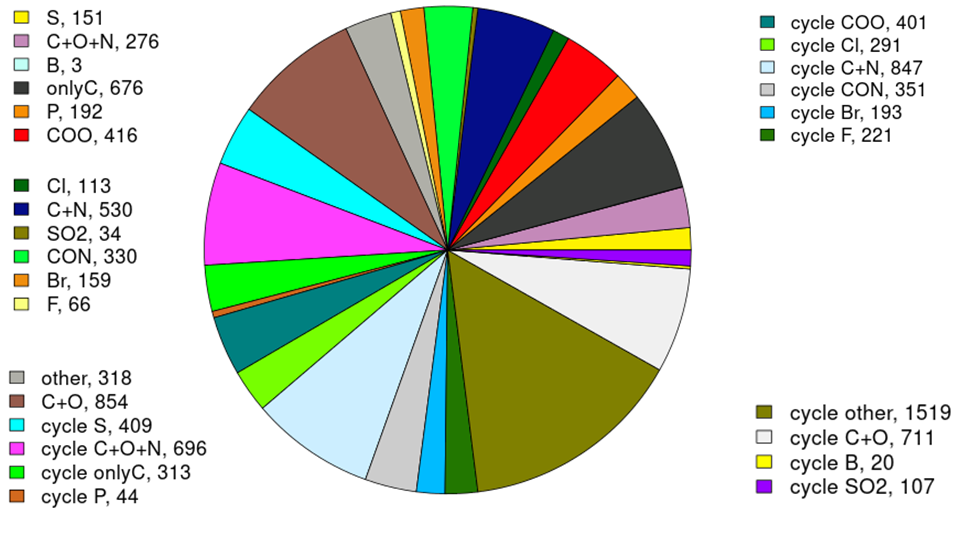


The LSR subgroup of 3,4-substituent catechol categorized as cycle C+O+N

S5. Visualization of the data clusterin


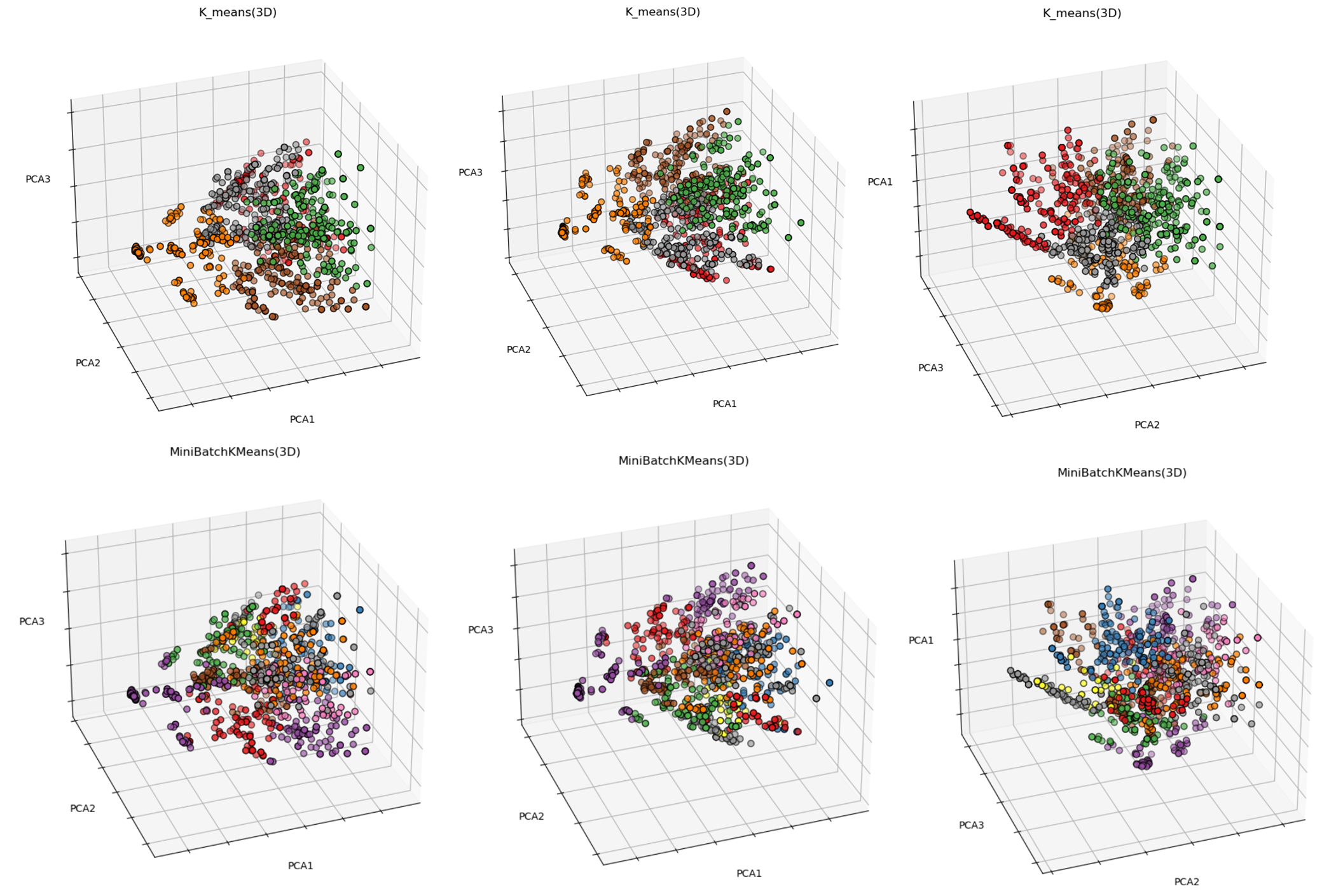


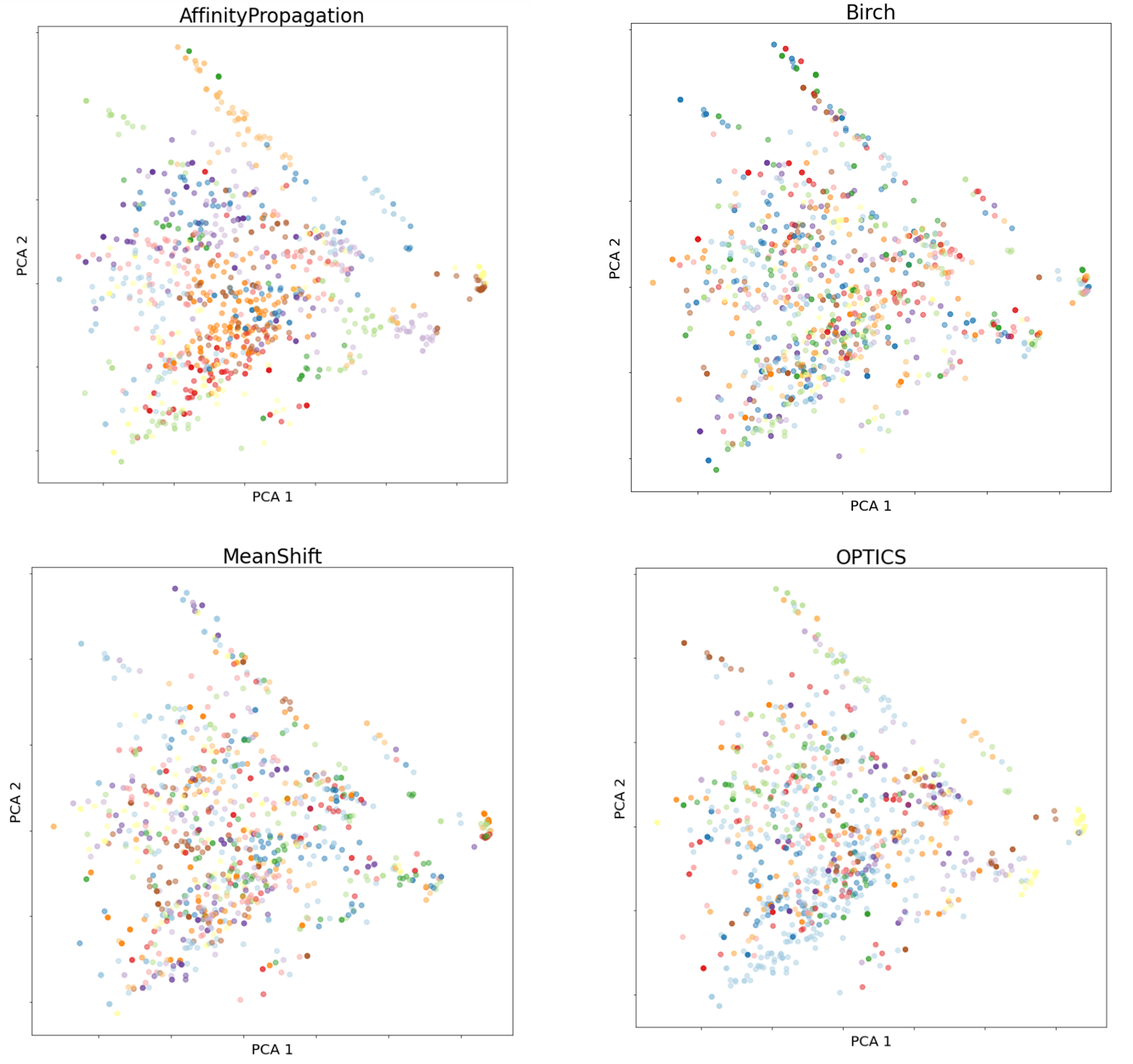


2D and 3D downscaled visualisation of 2048 molecular fingerprints from unsupervised clustering results of K-Means, Affinity Propgation,Birch,Mean Shift and OPTICS using PCA algorithm.
